# Seasonal metabolomics and antibacterial activity of *Abutilon pannosum* reveal positive interaction with the lytic phage CF01 against *Escherichia coli* O157:H7 (strain 161–84)

**DOI:** 10.3389/fmicb.2026.1916998

**Published:** 2026-08-21

**Authors:** Khaloud Mohammed Alarjani, Ohood Sallam, Tareq M. Osaili, Meriem Bekliz, Yousef Alhaj Hamoud, Attiat Elnaggar, Ali El-Keblawy

**Affiliations:** 1 Department of Botany and Microbiology, College of Science, King Saud University, Riyadh, Saudi Arabia; 2 Department of Botany and Microbiology, Faculty of Science, Menoufia University, Shebeen El-Kom, Egypt; 3 Research Institute for Medical and Health Sciences, University of Sharjah, Sharjah, United Arab Emirates; 4 Department of Clinical Nutrition and Dietetics, College of Health Sciences, University of Sharjah, Sharjah, United Arab Emirates; 5 Department of Nutrition and Food Technology, Faculty of Agriculture, Jordan University of Science and Technology, Irbid, Jordan; 6 Department of Applied Biology, College of Sciences, University of Sharjah, Sharjah, United Arab Emirates; 7 School of Energy and Environment, Southeast University, Nanjing, China; 8 Advanced Biotechnology Research Center, Research Institute of Sciences and Engineering, University of Sharjah, Sharjah, United Arab Emirates; 9 Department of Botany and Microbiology, College of Science, Alexandria University, Alexandria, Egypt; 10 Faculty of Pharmacy, Alsalam University, Tanta, Egypt

**Keywords:** *Abutilon pannosum*, antibacterial activity, bacteriophage CF01, desert medicinal plants, *Escherichia coli*, GC–MS, phage–extract interaction, seasonal metabolomics

## Abstract

**Introduction:**

Seasonal variation can influence plant metabolite composition and modify antimicrobial performance; however, the relationship between seasonal metabolomic changes and antibacterial activity remains poorly understood in many arid-land medicinal plants. To our knowledge, this is the first study to integrate GC–MS metabolomics, antibacterial screening, and phage–extract interaction testing in *Abutilon pannosum*.

**Methods:**

Leaf extracts of *Abutilon pannosum* collected during late summer (S1, September 2024), autumn (S2, November 2024), and winter (S3, February 2025) were analyzed using GC–MS. Antibacterial activity was evaluated against selected bacterial isolates using diffusion-based assays and MIC/MBC determination. The interaction between seasonal extracts and phage CF01 was further assessed against *Escherichia coli* O157:H7 under sub-MIC extract concentrations.

**Results:**

GC–MS analysis resolved the curated dataset into 350 putatively annotated parent metabolites, and PCA demonstrated clear season-dependent metabolomic separation, with 16 metabolites differing significantly among collection periods. S2 exhibited the broadest diversity of literature-supported antibacterial candidates, including *α*-linolenic acid, citronellol, dodecanoic acid, farnesol, ferulic acid, and 4-hydroxybenzoic acid, whereas S3 was enriched in caffeic acid, p-coumaric acid, D-limonene, and xylitol. Antibacterial activity varied according to season and bacterial isolate, with *Staphylococcus aureus* showing the highest susceptibility, while *Escherichia coli* exhibited weaker direct sensitivity. Phage–extract interaction assays demonstrated that CF01 enhanced *Escherichia coli* O157:H7 suppression at sub-MIC extract concentrations, particularly when extract-only treatment resulted in incomplete bacterial inhibition, with stronger enhancement patterns observed for S2 and S3 extracts.

**Discussion:**

Integrated metabolomics–bioactivity analysis identified citronellol and *α*-linolenic acid as candidate metabolites potentially associated with extract-mediated *Escherichia coli* reduction, while p-coumaric acid, citronellol, and α-linolenic acid showed positive associations with phage-enhanced antibacterial activity. These findings demonstrate that the antibacterial potential of *Abutilon pannosum* is season-dependent and support combining seasonal metabolomics, plant extract screening, and phage–extract strategies as an approach for identifying bioactive desert-plant metabolites against *Escherichia coli* O157:H7.

## Graphical abstract

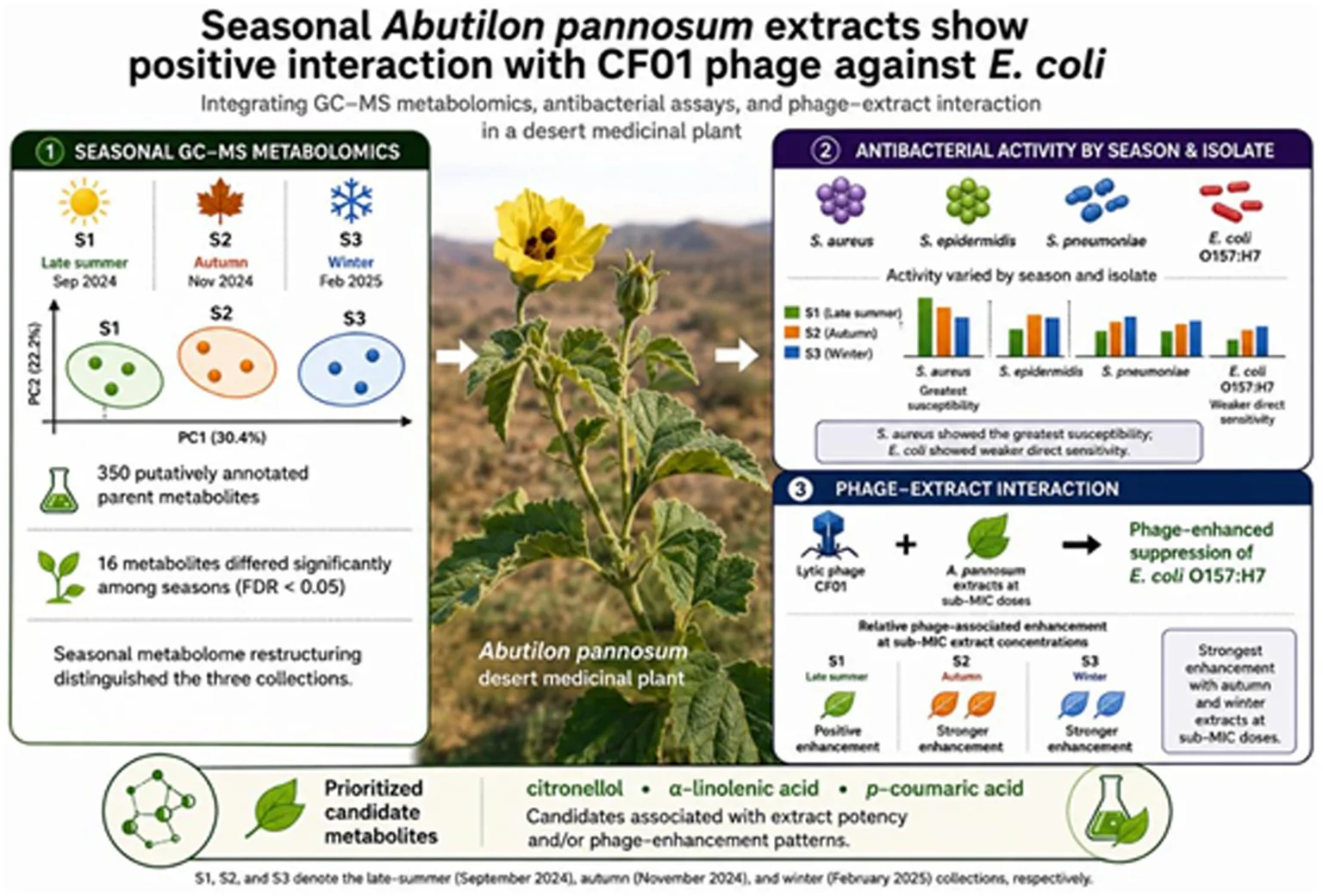

## Highlights

- *Abutilon pannosum* showed marked seasonal metabolome restructuring across three collections.
- The autumn extract contained the broadest set of antimicrobial-relevant metabolites.
- Extract antibacterial activity was season-dependent and isolate-dependent.
- CF01 enhanced *Escherichia coli* suppression when combined with seasonal sub-MIC extracts.
- Integrated analysis prioritized candidate metabolites associated with extract potency and phage enhancement.

## 1 Introduction

Antimicrobial resistance (AMR) continues to reduce the effectiveness of conventional antibacterial therapy and remains one of the most urgent challenges in clinical, food, and environmental microbiology. Global estimates indicate that bacterial AMR was directly responsible for approximately 1.27 million deaths and associated with nearly 5 million deaths in 2019 (Murray et al., 2022). The World Health Organization has also emphasized the continuing public-health importance of priority bacterial pathogens, including resistant strains of *Escherichia coli* and *Staphylococcus aureus*, highlighting the need for alternative or complementary antibacterial strategies (WHO, 2024). These concerns are particularly relevant for bacteria that occur across clinical, food-associated, and environmental interfaces, where new approaches should ideally combine antibacterial efficacy with biological specificity and reduced dependence on conventional antibiotics.

Plant-derived natural products remain an important source of antibacterial leads because they contain chemically diverse metabolites, including phenolic acids, terpenoids, flavonoids, alkaloids, fatty acids, and glycosides. Many of these compounds can inhibit bacteria through mechanisms that differ from those of conventional antibiotics, including membrane disruption, oxidative stress induction, efflux modulation, quorum-sensing interference, and biofilm suppression (Porras et al., 2020; Jubair et al., 2021; Bouyahya et al., 2022; Zouine et al., 2024). However, crude plant extracts are chemically complex mixtures, and their antibacterial performance cannot be interpreted reliably from inhibition assays alone. Metabolomics therefore provides a useful framework for linking extract-level antibacterial effects to candidate metabolite groups and for prioritizing compounds that may contribute to observed biological activity.

The antibacterial potential of medicinal plants is also shaped by seasonal and ecological conditions. Plant metabolite composition can shift in response to temperature, water availability, irradiance, phenological stage, and other environmental pressures, leading to changes in both the abundance of specific metabolites and the biological activity of extracts (Prinsloo and Nogemane, 2018). This is especially relevant in arid-region plants, where chronic exposure to drought, heat, high solar radiation, and oxidative stress may favor the accumulation of protective secondary metabolites. In antibacterial screening, seasonality should therefore not be treated merely as a sampling variable; it may determine whether a plant extract contains the chemical profile required for measurable bacterial inhibition. Accordingly, integrating seasonal metabolomics with antibacterial testing can help identify collection periods that maximize bioactive potential and improve the reproducibility of medicinal-plant screening.

*Abutilon pannosum* (G.Forst.) Schltdl., a member of Malvaceae family, is an arid-land species with emerging pharmacological relevance but limited microbiology-focused characterization. Previous studies have reported antibacterial activity and phytochemical constituents in *A. pannosum* leaf extracts (Survase et al., 2013; Abdulaziz, 2022), while fraction-based analyses have indicated cytotoxic potential and identifiable phytoconstituents in chloroform fractions (Al-Zharani et al., 2023). At the genus level, *Abutilon* has also been reviewed as a source of diverse phytochemicals, traditional medicinal uses, and pharmacological activities (Pandey et al., 2025). Despite this background, the seasonal metabolome of *A. pannosum* remains poorly characterized, and the relationship between collection season, metabolite composition, and antibacterial activity has not been systematically resolved. This gap limits the ability to identify optimal collection windows, interpret extract bioactivity, and prioritize candidate antibacterial metabolites in this species.

Bacteriophages provide an additional microbiological dimension for evaluating plant-derived antibacterial strategies. Lytic phages selectively infect susceptible bacterial hosts and are increasingly investigated as alternatives or adjuncts to conventional antimicrobials, particularly against resistant or persistent bacterial populations (Liu et al., 2020; Qin et al., 2024). While phage–antibiotic combinations have received increasing attention, interactions between phages and plant-derived extracts remain comparatively understudied. Such interactions may be beneficial, neutral, or antagonistic, depending on how extract chemistry affects bacterial physiology, phage adsorption, intracellular replication, or bacterial survival. Recent studies using plant extracts together with lytic phages show that botanical products can modify phage–bacteria interactions, with outcomes ranging from antagonistic to potentially beneficial depending on extract composition, dose, phage, and bacterial host (Stachurska et al., 2023).

A seasonal plant–phage framework is therefore valuable for two reasons. First, seasonally variable extracts may differ in direct antibacterial activity because their chemical composition changes across collection periods. Second, the same seasonal chemical shifts may alter compatibility with phage treatment, either by increasing bacterial vulnerability or by interfering with phage performance. Our recent work on the desert species *Cleome noeana* supports the value of integrating desert-plant metabolomics with antibacterial bioassays to prioritize candidate antimicrobial extracts and metabolites (El-Keblawy, Sallam et al., 2026). In the present study, we extend this concept to *A. pannosum* by evaluating whether seasonal extract chemistry is associated with both antibacterial activity and interactions with the previously characterized lytic *E. coli* phage CF01. CF01 has been described as a lytic Caudoviricetes phage targeting *E. coli*, with defined morphological, genomic, and functional characteristics, making it a suitable biological agent for testing extract–phage compatibility (Shaaban et al., 2026).

Accordingly, this study investigated whether seasonal shifts in the metabolome of *A. pannosum* are associated with changes in antibacterial activity and phage-assisted bacterial suppression. Specifically, we aimed to: (i) characterize the GC–MS-based seasonal metabolite profiles of *A. pannosum* extracts collected in late summer, autumn, and winter; (ii) evaluate extract antibacterial activity against selected bacterial isolates using diffusion- and dilution-based assays; (iii) determine whether CF01 enhances the activity of seasonal extracts against *E. coli* O157:H7; and (iv) integrate metabolomic and microbiological datasets to prioritize candidate metabolites associated with extract potency and phage-enhanced activity. We hypothesized that seasonal restructuring of *A. pannosum* chemistry would generate season-dependent antibacterial effects and that selected seasonal extracts would show enhanced bacterial suppression when combined with CF01. To our knowledge, this is the first study to integrate seasonal GC–MS metabolomics, antibacterial screening, and phage–extract interaction testing in *A. pannosum*.

## 2 Materials and methods

### 2.1 Plant material, seasonal collections, and extract preparation

#### 2.1.1 Plant collection and botanical verification

Leaf material of *Abutilon pannosum* (G. Forst.) Schltdl. was collected from Fujairah, United Arab Emirates (25.097962, 56.311487), during three seasonal collection periods: S1, late summer (September 2024); S2, autumn (November 2024); and S3, winter (February 2025). For each collection, three independent biological replicates were prepared from separately pooled leaf samples collected from different individuals. Each pooled sample was extracted, derivatized, and analyzed independently by GC–MS (n = 3 per season). In total, leaves were collected from at least 20 individuals representing a range of plant sizes to capture within-population variability and minimize bias associated with individual plant condition. The taxon was identified by Prof. Ali El-Keblawy using diagnostic morphological characteristics and the regional floristic treatment of Jongbloed et al. (2003). Representative voucher specimens from the three seasonal collections were deposited in the University of Sharjah Herbarium (UOS), Sharjah, United Arab Emirates, under the accession numbers UOS-APN-2024-09-480 (late-summer collection, September 2024), UOS-APN-2024-11-484 (autumn collection, November 2024), and UOS-APN-2025-02-490 (winter collection, February 2025). Plant material was collected from a publicly accessible site, and no special collection permit was required.

#### 2.1.2 Extract preparation

Collected leaves were washed with tap water, rinsed with distilled water to remove surface debris, and air-dried at room temperature in darkness. The dried material was ground into a fine powder using a laboratory grinder. Extraction was performed with 70% ethanol at a 1:6 (w/v) ratio, corresponding to 1 g of powdered material per 6 mL of solvent. Samples were incubated for 24 h on a rotary shaker at room temperature and then sonicated in a water bath for 1 h. The extracts were filtered through Whatman No. 1 filter paper, and the residues were re-extracted three times with fresh solvent to maximize metabolite recovery. The combined filtrates were concentrated under reduced pressure using a rotary evaporator at 30 °C, frozen at −80 °C, and lyophilized. The extraction yields of the seasonal *A. pannosum* extracts were 9.00, 7.00, and 6.67% (w/w) for S1 (September 2024), S2 (November 2024), and S3 (February 2025), respectively, calculated based on the dry weight of the plant material. The resulting dried extracts were stored at 4 °C until GC–MS profiling and antimicrobial testing.

### 2.2 GC–MS metabolomic profiling

Metabolomics analysis of plant extracts was conducted using gas chromatography–mass spectrometry (GC–MS) following the method described by El-Keblawy, Hamdy et al. (2026). Dried extracts were derivatized with 20 μL methoxyamine in pyridine (20 mg/mL) and 50 μL hexane, followed by vortexing and incubation in a 37 °C water bath for 90 min with vortexing every 30 min. Subsequently, 90 μL of N-trimethylsilyl-N-methyl trifluoroacetamide containing 1% trimethyl chlorosilane (MSTFA + 1% TMCS) was added. The samples were then incubated at 37 °C for 60 min. The derivatized mixture was filtered through a 0.45 μm nylon syringe filter (Membrane Solutions, Auburn, WA, USA) and analyzed using a GC-2010 gas chromatograph coupled to a QP-2010 Ultra mass spectrometer equipped with an AOC-20i + s autosampler (Shimadzu, Tokyo, Japan). Separation was achieved on an Rtx-5 ms capillary column (30 m × 0.25 mm i.d. × 0.25 μm film thickness; Restek, Bellefonte, PA, USA) using helium (99.9% purity) at 1.0 mL/min. The oven temperature program was 35 °C for 2 min, increased at 10 °C/min to 250 °C, then ramped at 20 °C/min to 320 °C and held for 23 min. Samples (1 μL) were injected in splitless mode at 250 °C. A solvent delay of 3 min was applied to exclude the solvent front, which eluted during approximately the first 2 min; mass-spectral acquisition therefore began at 3.0 min. The mass spectrometer operated in electron impact mode (70 eV) with the ion source and interface temperatures set to 240 °C and 250 °C, respectively. Data were acquired in scan mode over m/z 35–450 at a scan speed of 1,428 u s^−1^ (Al-Nablsi et al., 2022). Data acquisition and processing were performed using the instrument-associated GC–MS data-processing software, and electron-ionization mass spectra were compared with entries in the NIST 14 Mass Spectral Library. A numerical library-match threshold could not be verified from the available analytical records. Retention-index confirmation and authentic reference standards were not used. Consequently, the reported compound assignments were classified as putative library-based annotations rather than confirmed metabolite identifications, in accordance with metabolomics reporting recommendations (Sumner et al., 2007). Compounds potentially affected by isomeric overlap, derivatization, or ambiguous spectral matching should therefore be interpreted cautiously.

### 2.3 GC–MS data curation and parent-metabolite matrix construction

Raw GC–MS data were curated prior to statistical analysis to distinguish analytical features from biologically interpretable metabolites. Initial feature lists were inspected to remove entries consistent with derivatization reagents, siloxane/background contamination, residual solvent-related contaminants, or preparation-related artifacts. Retained derivatized entries, including oxime, methyloxime, ethyloxime, and trimethylsilyl forms, were not interpreted as independent biological metabolites when they represented the same likely parent compound. Instead, derivatized, duplicate, and isomeric entries were consolidated under parent-metabolite names for downstream interpretation.

This workflow generated two complementary data layers: (i) a retained feature-level matrix used for analytical transparency and (ii) a parent-metabolite matrix used for seasonal comparison, antimicrobial interpretation, and metabolite–bioactivity integration. After curation and consolidation, the final parent-metabolite dataset comprised 350 parent metabolites. The complete processed parent-metabolite dataset is provided in Supplementary Table S3. The table presents the 350 consolidated parent metabolites used for seasonal analysis, including the contributing analytical features, seasonal mean peak areas, standard deviations, replicate detection frequencies, and the collection period showing the highest mean abundance. Replicate-level peak areas for the 363 retained analytical features and the corresponding derivatized-feature-to-parent-metabolite mapping are provided in Supplementary Data S1. Because authentic reference standards were not used, all compound assignments are reported as putative library-based annotations rather than confirmed metabolite identifications.

### 2.4 Bacterial isolates, culture conditions, and bacteriophage CF01

All bacterial isolates were cultured on Mueller–Hinton medium under aerobic incubation at 37 °C for 18–24 h. Although enriched media are commonly recommended for optimal growth of fastidious bacteria such as *Streptococcus pneumoniae*, the tested *S. pneumoniae* ATCC 6301 strain showed sufficient growth on Mueller–Hinton medium under the applied laboratory conditions, allowing its use in the antibacterial screening assays.

The bacteriophage used in the *E. coli* interaction assay was CF01, a previously characterized lytic *E. coli* phage. CF01 was selected because its morphological, genomic, and functional properties had been previously described, including its classification within Caudoviricetes, lytic behavior, host-targeting characteristics, and absence of undesirable genetic features, such as lysogeny-associated or antimicrobial-resistance determinants (Shaaban et al., 2026). Therefore, CF01 was not re-characterized in the present study and was used as a defined biological agent to evaluate the phage–extract interaction.

CF01 working stocks were propagated on *E. coli* O157:H7 strain 161–84 (Canadian Science Center for Human and Animal Health, Ottawa, Canada) using the double-layer agar method (Kutter and Sulakvelidze, 2004). Plaque-purified lysates were clarified by centrifugation, filtered through a 0.22 μm membrane, titrated as PFU/mL, and stored at 4 °C until use.

For phage–extract interaction assays, overnight *E. coli* O157:H7 cultures were adjusted to approximately 1 × 10^6^ CFU/mL prior to treatment. CF01 was applied at a final concentration of approximately 1 × 10^5^ PFU/mL, corresponding to an MOI of 0.1.

#### 2.4.1 Phage-extract compatibility assay

To evaluate whether the seasonal *Abutilon pannosum* extracts affected CF01 infectivity, CF01 (3 × 10^8^ PFU/mL) was incubated separately with each seasonal extract (100 mg/mL) for 24 h at 37 °C under the same conditions used in the phage–extract interaction assay. A phage suspension incubated under identical conditions in the absence of extract served as the control. After incubation, residual phage infectivity was determined using the double-layer agar plaque assay and expressed as plaque-forming units (PFU)/mL. The compatibility assay was performed in three independent replicates (*n* = 3).

### 2.5 Extract-only antibacterial assays

Bacterial inocula were standardized to approximately 0.5 McFarland prior to antibacterial testing. All antibacterial assays, including disc diffusion, well diffusion, and MIC/MBC determinations, were performed using three independent biological replicates, each analyzed in triplicate (technical replicates). Results are presented as mean ± standard deviation (SD).

#### 2.5.1 Disc diffusion assay

Antibacterial activity of the seasonal *A. pannosum* extracts was screened using the disc diffusion method, following the general principles of standardized agar diffusion testing (Bauer et al., 1966). Sterile 6-mm-diameter paper discs were impregnated with 30 μL of plant extract prepared from a 100 mg/mL stock solution, dried under sterile conditions, and placed on Mueller–Hinton agar plates previously seeded with standardized bacterial inocula. Plates were incubated at 37 °C for 18–24 h, and inhibition zones were measured in millimeters.

Chloramphenicol (30 μg/disc) was used as the positive reference antibiotic for *E. coli* O157:H7, whereas tetracycline (30 μg/disc) was used for the Gram-positive isolates, including *S. aureus* ATCC 29213, *Staphylococcus epidermidis* ATCC 14990, and *S. pneumoniae* ATCC 6301. Solvent-treated discs containing dimethyl sulfoxide (DMSO, 1% v/v; the same concentration used for extract preparation) were included as negative controls and showed no inhibition zones against any tested isolate.

#### 2.5.2 Well diffusion assay

A complementary agar well-diffusion assay was performed to assess extract activity in a second diffusion-based format (Balouiri et al., 2016). Wells (6 mm diameter) were prepared in Mueller–Hinton agar previously inoculated with the bacterial suspension, and 30 μL of the extract solution was added to each well. Plates were allowed to stand briefly at room temperature to facilitate diffusion, then incubated at 37 °C for 18–24 h. Zones of inhibition were measured in millimeters. The same positive and negative controls used in the disc diffusion assay were applied in the well diffusion assay.

#### 2.5.3 MIC and MBC determination

Minimum inhibitory concentration (MIC) values were determined using broth microdilution according to established antimicrobial susceptibility testing principles (Andrews, 2001; CLSI, 2012). Crude extracts were prepared at 250 mg/mL and two-fold serially diluted in Mueller–Hinton broth to obtain final concentrations ranging from 125 to 1.95 mg/mL (125, 62.5, 31.25, 15.60, 7.80, 3.90, and 1.95 mg/mL). Wells were inoculated with standardized bacterial suspensions and incubated at 37 °C for 18–24 h. The plant extracts were dissolved in dimethyl sulfoxide (DMSO), and the final concentration of DMSO in all MIC assay wells was 1% (v/v). A solvent control containing 1% (v/v) DMSO was included and showed no inhibitory effect on bacterial growth. MIC was defined as the lowest extract concentration at which no visible bacterial growth was observed.

For minimum bactericidal concentration (MBC), aliquots from wells showing no visible growth were subcultured onto agar plates and incubated under the same conditions. MBC was defined as the lowest concentration yielding no recoverable colony growth. When both MIC and MBC were measurable, the MBC/MIC ratio was used to classify the activity profile; MBC/MIC ≤ 4 was used as an operational indicator consistent with bactericidal activity. MIC and MBC values were considered reproducible only when consistent across the three independent biological replicates.

Isolate–season combinations recorded as 0 in the raw MIC/MBC sheets were interpreted as “no detectable activity within the tested concentration range,” not as true zero MIC or MBC values.

### 2.6 Phage–extract interaction assay against *Escherichia coli* O157:H7

To evaluate whether CF01 enhanced the antibacterial activity of seasonal *A. pannosum* extracts, *E. coli* O157:H7 cultures were adjusted to approximately 1 × 10^6^ CFU/mL and exposed to extract alone, CF01 alone, or extract–phage combinations. For each seasonal extract, interaction assays were conducted at sub-MIC extract levels corresponding to 1/2 MIC, 1/4 MIC, and 1/8 MIC. Because the MIC values of the S1, S2, and S3 extracts against *E. coli* O157:H7 were 125 mg/mL, the actual concentrations used were 62.5, 31.25, and 15.63 mg/mL, respectively. The 1 x MIC condition (125 mg/mL) was retained as a reference where applicable but was interpreted cautiously because complete killing by the extract alone can create a floor effect that prevents formal interpretation of synergy.

CF01 was added to the bacterial suspension to achieve a final phage concentration of approximately 1 × 10^5^ plaque-forming units (PFU)/mL in the assay mixture. The initial bacterial density was approximately 1 × 10^6^ colony-forming units (CFU)/mL, corresponding to a multiplicity of infection (MOI) of 0.1. Untreated bacterial cultures served as positive growth controls. Cultures treated with CF01 alone were included to distinguish phage-specific antibacterial effects from extract-mediated activity.

Cultures were incubated at 37 °C, and viable bacterial counts were determined at 0 h and 24 h by ten-fold serial dilution (10^−1^–10^−7^) and plating on nutrient agar. Aliquots of 100 μL from appropriate dilutions were spread-plated in duplicate, and plates containing 30–300 colonies were used for enumeration. The assay was performed using three independent biological replicates, while duplicate plating of each dilution served as technical replication for bacterial enumeration. The lower detection limit was approximately 10 CFU/mL. Results were expressed as CFU/mL for microbiological interpretation and transformed to log10(CFU/mL + 1) for statistical analysis. Samples with no detectable (ND) colonies were assigned a value of 0 CFU/mL prior to transformation using log10(CFU/mL + 1).

The design and interpretation followed established approaches for phage–antibacterial combination and time-kill analyses (Nikolic et al., 2022; Kunz Coyne et al., 2024). Interaction effects were interpreted using a conservative time-kill framework. No checkerboard assay, fractional inhibitory concentration index, Bliss-independence model, or other formal additivity–synergy method was applied; therefore, the combinations were not classified as definitively synergistic or additive. Combinations producing markedly greater bacterial reduction than the corresponding single treatments were interpreted as positive phage–extract interactions, and the term “enhanced activity” was used to describe effects exceeding those of the corresponding single treatments. Bactericidal activity was defined as a reduction of at least 3 log10 CFU/mL relative to baseline bacterial counts.

### 2.7 Literature-based antimicrobial annotation of selected metabolites

Two complementary metabolite-selection approaches were used. First, all 350 parent metabolites were evaluated for differential abundance, and metabolites with Benjamini–Hochberg-adjusted FDR values < 0.05 were classified as statistically significant seasonal metabolites. Separately, selected parent metabolites detected in *A. pannosum* extracts were annotated for antimicrobial relevance using experimentally supported literature evidence. Evidence was considered supportive when a metabolite or closely corresponding parent compound had been tested against bacterial or fungal organisms using measurable endpoints such as MIC, MBC, MFC, MBIC, inhibition percentage, or log10 CFU reduction. This targeted literature-based assessment was applied to the complete set of 350 parent metabolites and was conducted independently of metabolite abundance, fold change, or FDR significance. It yielded 14 literature-supported antimicrobial candidates. These annotations were used to classify highlighted metabolites as antibacterial, antibacterial/antifungal, limited or conditional antimicrobial evidence, or weak antifungal evidence.

The statistically significant seasonal set and the literature-supported antimicrobial set therefore served different but complementary purposes and were not expected to overlap completely. Palmitic acid met both criteria because it differed significantly among seasons and has reported antimicrobial and antibiofilm activity. The antimicrobial annotations were used only for candidate prioritization and biological interpretation. They were not treated as proof that individual metabolites were solely responsible for the activity of the crude extracts.

### 2.8 Statistical and integrative analysis

#### 2.8.1 Seasonal metabolomics

The curated parent-metabolite matrix was normalized and log-transformed before statistical analysis. Principal component analysis (PCA) was used as an unsupervised method to visualize seasonal clustering among samples. Supervised multivariate analyses, including PLS-DA, OPLS-DA, and VIP-score analysis, were not performed in the present study. Differential abundance of parent metabolites across S1, S2, and S3 was evaluated using the curated parent-metabolite matrix, and multiple testing correction was performed using the Benjamini–Hochberg false discovery rate procedure (Benjamini and Hochberg, 1995). Metabolites with FDR < 0.05 were considered significantly seasonal.

#### 2.8.2 Extract-only antibacterial assays

All experiments were performed in triplicate, and results are expressed as mean ± standard deviation (SD). For disc diffusion and well diffusion assays, inhibition zone data were analyzed using a two-way ANOVA with bacterial isolate and seasonal collection as fixed factors. The isolate × season interaction was used to determine whether seasonal effects differed among bacterial targets. Where significant interactions were detected, isolate-level *post hoc* comparisons were used to interpret the seasonal response patterns. Datasets showing no activity, zero variance, or non-detectable values were excluded from inferential statistical analysis because they did not satisfy the assumptions required for ANOVA. MIC and MBC data were summarized descriptively because several isolate–season combinations showed no detectable activity within the tested concentration range, making inferential comparison inappropriate.

#### 2.8.3 Phage–extract interaction analysis

For the phage–extract interaction assay, the primary response variable was log10(CFU/mL + 1) at 24 h. Separate factorial models were fitted for each seasonal extract, with phage presence (absent/present) and extract dose as fixed factors. The phage × dose interaction was used to assess whether the effect of CF01 depended on extract concentration. Extract-only and extract + phage treatments at matched doses were further compared using adjusted pairwise tests. Additional descriptive analyses of log10 reductions from baseline and relative to the untreated control were used to support the interpretation. For each assay, experiments were performed in triplicate (*n* = 3), and results are expressed as mean ± SD. ANOVA assumptions, including normality and homogeneity of variance, were assessed using the Shapiro–Wilk test and Levene’s test, respectively. When significant differences were detected, Tukey’s *post hoc* test was applied for multiple comparisons. Non-detectable CFU values were handled as described above using log10(CFU/mL + 1) transformation.

#### 2.8.4 Metabolite–bioactivity integration

To relate seasonal chemistry to antibacterial performance, selected metabolites with literature-supported antimicrobial relevance were integrated with aligned seasonal bioactivity metrics, including extract-only antibacterial activity, *E. coli* extract-only reduction, *E. coli* combination reduction, and phage-enhancement magnitude. Candidate prioritization was based on the convergence of three criteria: literature-supported antimicrobial relevance, seasonal enrichment, and positive alignment with extract-only *E. coli* reduction and/or phage-enhancement magnitude. Metabolite abundance alone was not used as a prioritization criterion. Because only three seasonal groups were available at the season-summary level, this integration was treated as descriptive and hypothesis-generating rather than causal. Exploratory matched-replicate associations were summarized using Spearman correlation coefficients, with raw and multiple-testing-adjusted significance values reported as supportive evidence where appropriate. All statistical analyses and visualizations were performed in Python 3.13 using standard scientific libraries for data handling, statistics, and figure generation.

## 3 Results

### 3.1 Seasonal restructuring of the *Abutilon pannosum* metabolome

**Figure 1:**
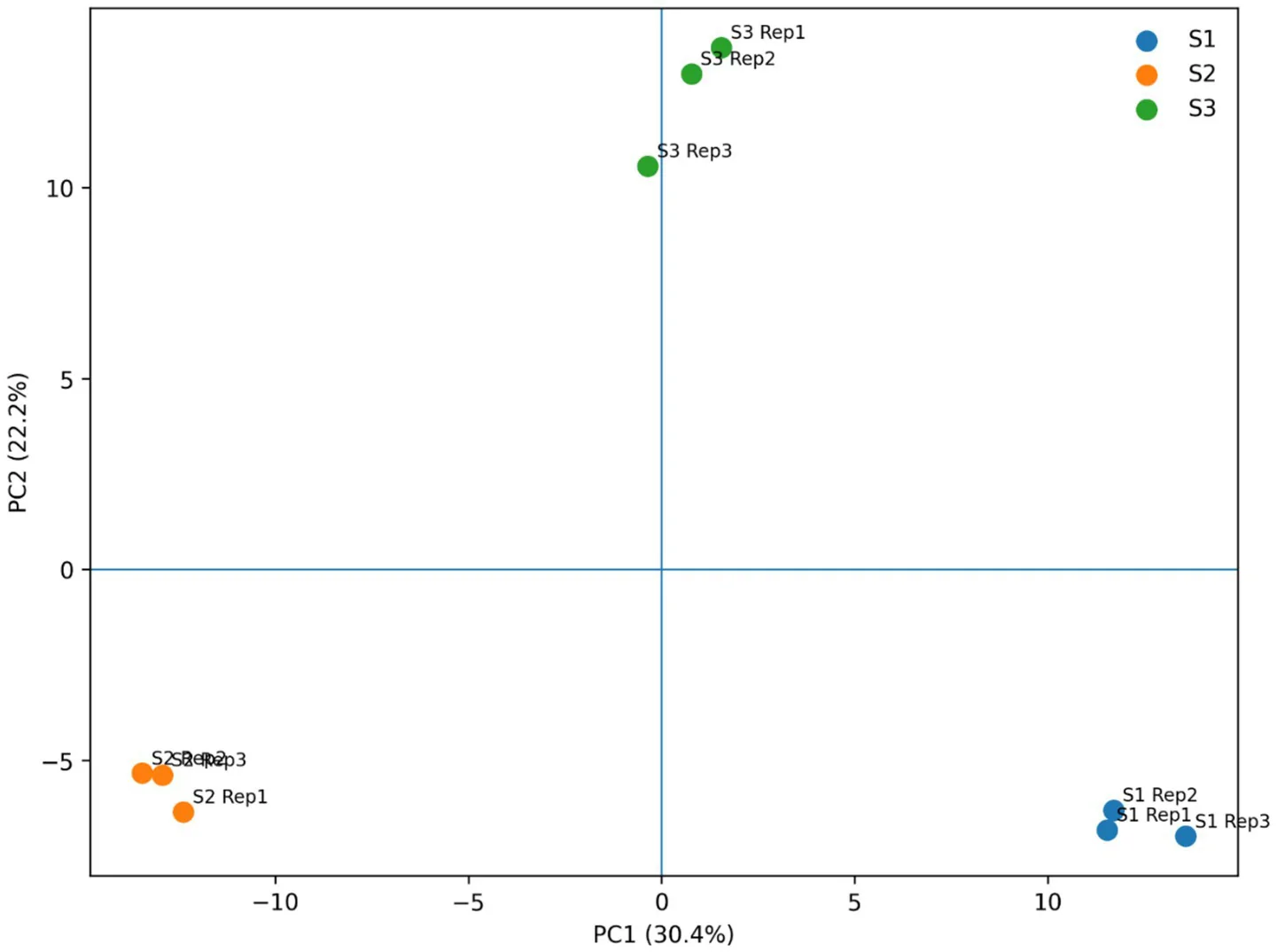
Principal component analysis (PCA) of the normalized and log-transformed curated parent-metabolite profiles of *Abutilon pannosum* across three seasonal collections (S1, S2, and S3). Each point represents one biological independent replicate (*n* = 3 per season). S1, S2, and S3 denote the late-summer (September 2024), autumn (November 2024), and winter (February 2025) collections, respectively.

After curation and consolidation of derivatized and redundant GC–MS features, the seasonal metabolomic dataset of *A. pannosum* was resolved into 350 parent metabolites for comparison among the three collection periods: S1, late summer (September 2024); S2, autumn (November 2024); and S3, winter (February 2025). The complete list of the 350 consolidated parent metabolites and their seasonal abundance profiles is provided in Supplementary Table S3. Principal component analysis (PCA) of the normalized and log-transformed parent-metabolite matrix showed clear separation among the three seasonal collections, indicating a pronounced seasonal effect on metabolite composition (Figure 1). The first two principal components explained 30.4 and 22.2% of the total variance, respectively, and biological replicates clustered closely within each season, supporting the consistency of the seasonal metabolomic profiles.

Seasonal differences were further resolved at the individual-metabolite level using the heatmap of significantly varying parent metabolites (Supplementary Figure S1). Sixteen parent metabolites differed significantly among collections after Benjamini–Hochberg false discovery rate correction (FDR < 0.05), indicating that the PCA separation was driven by coordinated shifts in a defined subset of compounds rather than random variation. These 16 metabolites constituted the statistically defined seasonal set and were distinct from the 14 literature-supported antimicrobial candidates selected independently from the complete 350-metabolite dataset for targeted bioactivity interpretation. Palmitic acid met both criteria. Autumn samples were characterized by enrichment of maltose relative to late summer (log2FC S2/S1 = 3.60; approximately 12.1-fold) and increased 1-linolenoylglycerol (log2FC S2/S1 = 2.16; approximately 4.5-fold), whereas propanedioic acid, ribonic acid, and 2-butenedioic acid were reduced in S2 relative to S1. Winter samples were distinguished by increased hydracrylic acid relative to late summer (log2FC S3/S1 = 2.05; approximately 4.1-fold) and higher D-arabinose relative to autumn (log2FC S3/S2 = 1.84; approximately 3.6-fold). Together, these results demonstrate marked seasonal restructuring of the *A. pannosum* metabolome at both global and individual-metabolite levels.

### 3.2 Seasonal enrichment of antimicrobial-relevant metabolites

**Figure 2:**
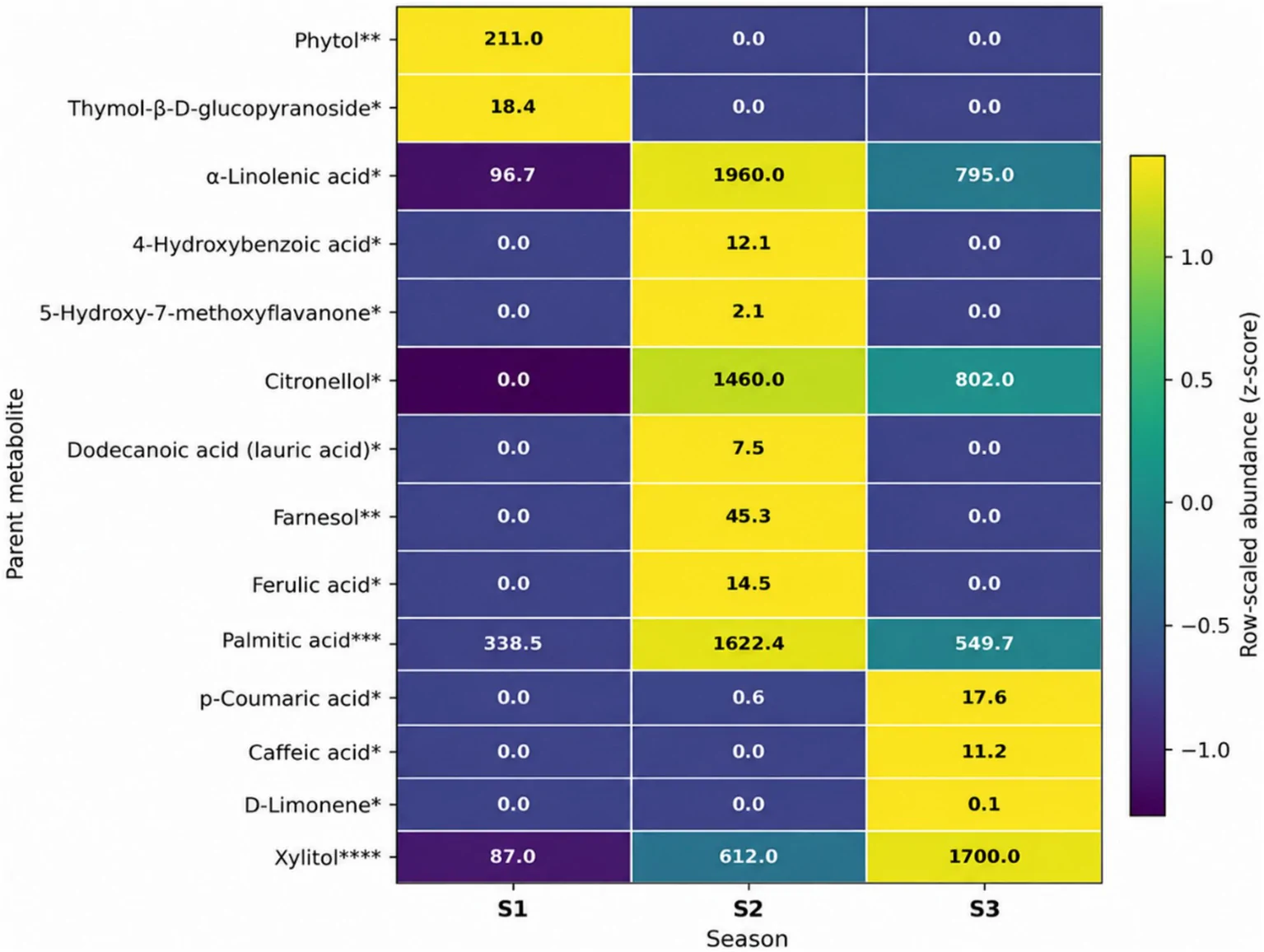
Heatmap of the 14 parent metabolites with literature-supported antimicrobial relevance in *Abutilon pannosum* across three seasonal collections (S1, S2 and S3). Values inside cells represent the integrated GC–MS peak area, expressed as ×10^6^ arbitrary units, from three biological replicates (*n* = 3 per season), and colors indicate row-scaled abundance (*z*-score). S1, S2 and S3 denote the late-summer (September 2024), autumn (November 2024), and winter (February 2025) collections, respectively. Metabolites were selected based on literature-supported antimicrobial evidence, independently of statistical significance. Asterisks associated with metabolite names correspond to the antimicrobial relevance categories defined in Table 1. *Antibacterial; **Antibacterial and antifungal; ***Limited or conditional antimicrobial evidence; ****Antifungal with weak direct evidence.

To assess the potential biological relevance of the seasonal metabolite shifts, 14 parent metabolites with literature-supported antimicrobial relevance were examined in relation to their seasonal abundance patterns (Figure 2; Table 1). These metabolites were selected from the complete set of 350 parent metabolites based on experimentally supported antimicrobial evidence, independently of metabolite abundance, seasonal fold change, or FDR significance. These metabolites showed clear season-dependent distribution, indicating that the antimicrobial potential of *A. pannosum* extracts may vary with the seasonal enrichment of specific bioactive candidates rather than with overall metabolite abundance alone.

**Table 1:** Literature-supported antimicrobial evidence for selected parent metabolites identified in *Abutilon pannosum*.

Parent metabolite	Tested organism(s)	Reported endpoint(s)	Representative experimental evidence	Selected supporting references
Phytol**	*Pseudomonas aeruginosa*; *Candida* spp.	MIC 20 μg/mL against *Pseudomonas aerugin*; antifungal activity reported against *Candida* spp.	Reported antibacterial activity against *Pseudomonas aerugin* and antifungal activity against *Candida* spp. in experimental systems.	Lee et al. (2016); Lima et al. (2020)
Thymol-*β*-D-glucopyranoside*	*Campylobacter jejuni; Salmonella enterica serovar Typhimurium; Escherichia coli K88*	1.10–2.32 log10 CFU/mL reduction at 1 mM after 24 h	Showed antibacterial activity in gut-content and in vitro models, particularly under microbiota-associated conditions.	Epps et al. (2015); Levent et al. (2016)
α-Linolenic acid*	*Bacillus cereus; Staphylococcus aureus*	MIC 20 ppm (*Bacillus cereus*); 50 ppm (*Staphylococcus aureus*)	Demonstrated antibacterial activity in broth microdilution assays; broader antibacterial effects of fatty acids also documented.	Lee et al. (2002); Casillas-Vargas et al. (2021)
4-Hydroxybenzoic acid*	Gram-negative bacteria: *Pseudomonas aeruginosa* KCTC 1628, *Salmonella typhimurium* ATCC 19430, *Escherichia coli* ATCC 10536; Gram-positive bacteria: *Staphylococcus aureus* ATCC 5838, *Staphylococcus epidermidis* ATCC 12228, *and Candida albicans* ATCC 10231	IC₅₀ values of 619, 688, 458, 926, 355, and 1,380 μg/mL, respectively.	Demonstrated antimicrobial activity against selected Gram-negative and Gram-positive bacteria and *Candida albicans*, with strain-dependent potency.	Cho et al. (1998)
5-Hydroxy-7-methoxyflavanone*	*Pseudomonas aeruginosa*	MIC 125 μg/mL	Reported antibacterial activity as a flavonoid antimicrobial candidate in experimental testing.	Ahmad et al. (2014)
Citronellol*	*Escherichia coli*; *Staphylococcus aureus*	MIC 5 μg/mL for *Escherichia coli* and 375 μg/mL for *Staphylococcus aureus*	Showed antibacterial activity with alterations in cell-surface properties, membrane integrity disruption, and K^+^ leakage.	Lopez-Romero et al. (2015)
Dodecanoic acid (lauric acid)*	Broad range of bacteria, especially Gram-positive bacteria; *Staphylococcus aureus*	MIC 156 μg/mL against *Staphylococcus aureus*	Demonstrated antibacterial activity including growth inhibition of *Staphylococcus aureus*, membrane disruption, ROS induction, and reduced viable counts.	Casillas-Vargas et al. (2021); Liu et al. (2024)
Farnesol**	*Staphylococcus aureus* MSSA/MRSA; *Candida albicans*; *Candida parapsilosis*; *Candida tropicalis*; *Paracoccidioides brasiliensis*	200 μM caused ~0.5-log biofilm reduction in *Staphylococcus aureus*; antifungal MICs 75–150, 75–300, 18.75–75, and 25 μM, respectively	Reported antibacterial biofilm-inhibitory activity against *Staphylococcus aureus* and antifungal activity against several pathogenic fungi.	Jabra-Rizk et al. (2006); Cordeiro et al. (2013); Derengowski et al. (2009)
Ferulic acid*	*Escherichia coli*; *Pseudomonas aeruginosa*; *Staphylococcus aureus*; *Listeria monocytogenes*	MIC 1500 μg/mL for *Escherichia coli*, 500 μg/mL for *Pseudomonas aeruginosa*, 1750 μg/mL for *Staphylococcus aureus*, and 2000 μg/mL for *Listeria monocytogenes*	Phenolic acid with experimentally supported antibacterial activity, though potency varies by species and assay conditions.	Borges et al. (2013)
p-Coumaric acid*	*Staphylococcus aureus*; *Streptococcus pneumoniae*; *Bacillus subtilis*; *Escherichia coli*; *Shigella dysenteriae*; *Salmonella typhimurium*	MIC values ranging from 10–80 μg/mL	Phenolic acid with experimentally supported antibacterial activity, although potency is method- and strain-dependent.	Lou et al. (2012)
Caffeic acid*	*Staphylococcus aureus and related strains*	MIC 256–1,024 μg/mL	Reported antibacterial activity, especially against Gram-positive bacteria, but with moderate potency.	Borges et al. (2013); Kępa et al. (2018)
D-Limonene*	*Listeria monocytogenes*	MIC 20 mL/L	Monoterpene with experimental antibacterial activity, particularly against foodborne bacteria.	Han et al. (2020)
Xylitol****	*Candida albicans*	MIC 20 × 10^4 μg/mL; MFC 40 × 10^4 μg/mL	Showed weak direct antifungal activity at high concentrations; interpreted as low-potency evidence.	Talattof et al. (2012)
Palmitic acid***	*Candida tropicalis*	MIC 1 mg/mL; inhibition of biofilm-associated virulence traits	Demonstrated antifungal and antibiofilm activity against *Candida tropicalis*, including effects on biofilm formation, cell-surface hydrophobicity, ergosterol biosynthesis, and virulence-associated enzymes.	Prasath et al. (2020)

*Antibacterial; **Antibacterial and antifungal; ***Limited or conditional antimicrobial evidence; ****Antifungal with weak direct evidence.

The autumn collection (S2) showed the broadest enrichment of metabolites with reported antibacterial and/or antifungal relevance. In particular, *α*-linolenic acid and citronellol were highly abundant in S2, while dodecanoic acid, farnesol, ferulic acid, 4-hydroxybenzoic acid, 5-hydroxy-7-methoxyflavanone, and palmitic acid were also associated with the autumn profile. This pattern suggests that S2 was enriched in metabolites with plausible membrane-active, growth-inhibitory, or antibiofilm relevance. In contrast, the late-summer collection (S1) was characterized mainly by phytol and thymol-*β*-D-glucopyranoside, whereas the winter collection (S3) was associated with caffeic acid, p-coumaric acid, D-limonene, and xylitol.

Although these seasonal patterns do not establish causality, they identify plausible candidate metabolites that may contribute to variation in extract-level antimicrobial activity among collections. Overall, the autumn metabolome contained the broadest set of literature-supported antimicrobial candidates, whereas the late-summer and winter collections were characterized by smaller but distinct sets of enriched metabolites. These findings provide the chemical basis for subsequent integration of metabolomic and antimicrobial datasets.

### Extract-only antibacterial activity of seasonal *Abutilon pannosum* extracts against selected bacterial isolates

**Figure 3:**
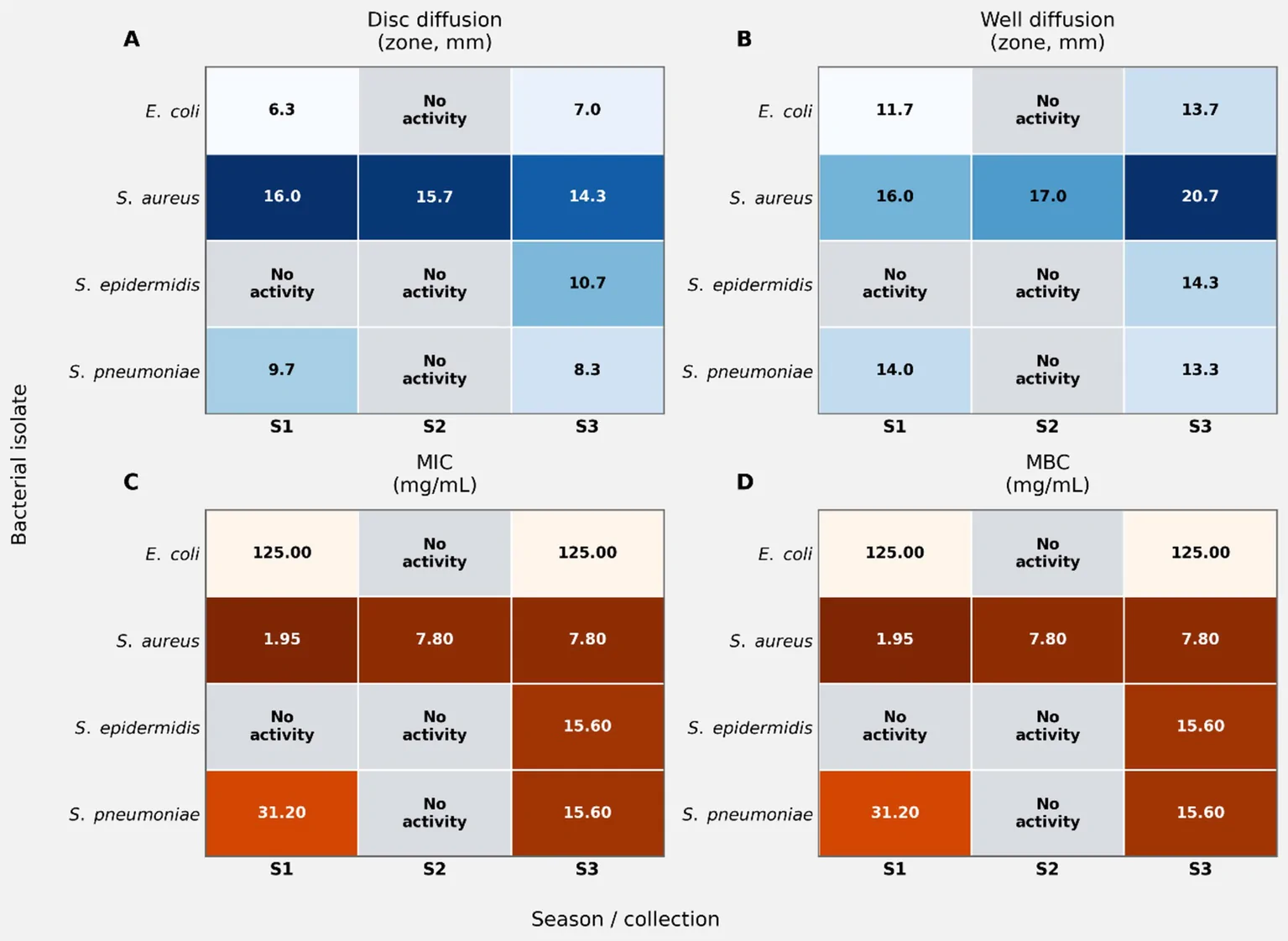
Antimicrobial activity of seasonal *Abutilon pannosum* extracts against selected bacterial isolates across S1–S3. In diffusion assays, larger inhibition zones indicate stronger activity, whereas in MIC and MBC assays, lower values indicate stronger inhibitory or bactericidal activity. “No activity” indicates no detectable effect under the tested conditions. Data are presented from three independent biological replicates (*n* = 3).

The antibacterial activity of seasonal *A. pannosum* extracts differed by bacterial isolate and collection period (Figure 3). Across the diffusion assays, *S. aureus* was the most consistently susceptible isolate, whereas *E. coli* showed weaker direct susceptibility. The response was also season-dependent but not uniform across bacteria. For example, the S2 extract showed no detectable diffusion-based activity against *E. coli*, *S. epidermidis*, or *S. pneumoniae*, whereas the S3 extract showed measurable activity against all tested isolates and was the only collection with detectable activity against *S. epidermidis*. This pattern indicates that seasonal changes in extract activity were isolate-specific rather than uniformly stronger or weaker across all bacteria.

Two-way ANOVA supported this interpretation, showing significant effects of bacterial isolate, season, and the isolate × season interaction in both disc diffusion and well diffusion assays (Table 2). The significant interaction term indicates that seasonal variation in antibacterial activity depended on the target bacterium. The well diffusion assay generally produced clearer activity patterns than disc diffusion, particularly for *S. aureus*, *E. coli*, and *S. epidermidis*, suggesting that the assay format influenced the apparent strength of the extract’s activity.

**Table 2:** Two-way ANOVA of disc diffusion and well diffusion responses of seasonal *Abutilon pannosum* extracts against tested bacterial isolates, showing the effects of isolate, season/collection, and their interaction.

Assay	Effect	df	F	*p*-value
Disc diffusion	Isolate	3, 24	353.58	<0.0001
	Season	2, 24	157.44	<0.0001
	Isolate × Season	6, 24	55.62	<0.0001
Well diffusion	Isolate	3, 24	312.87	<0.0001
	Season	2, 24	428.47	<0.0001
	Isolate × Season	6, 24	72.47	<0.0001

Inferential analysis was applied to disc diffusion and well diffusion only. Significant isolate × season interactions justified isolate-level post hoc comparisons.

These differences should be interpreted with caution because inhibition zone size is influenced not only by antibacterial potency but also by the diffusion capacity of extract constituents through agar. Therefore, weak or absent diffusion-based activity does not necessarily exclude antibacterial potential.

MIC and MBC profiles were broadly consistent with the diffusion-based screening but were interpreted descriptively because several isolate–season combinations showed no detectable activity within the tested concentration range (Table 3). *S. aureus* showed the greatest susceptibility, with the lowest MIC and MBC values observed for the S1 extract. In contrast, *E. coli* required much higher extract concentrations and showed no detectable activity in S2. *S. epidermidis* showed measurable MIC/MBC activity only in S3, whereas *S. pneumoniae* was active in S1 and S3, with lower MIC/MBC values in S3. Where MIC and MBC were measurable, the values were identical, indicating a bactericidal profile under the tested conditions.

**Table 3:** Descriptive summary of minimum inhibitory concentration (MIC) and minimum bactericidal concentration (MBC) values of seasonal *Abutilon pannosum* extracts against tested bacterial isolates.

Isolate	MIC S1	MIC S2	MIC S3	MBC S1	MBC S2	MBC S3
*Escherichia coli*	125.0 ± 0.00	125.0 ± 0.00	125.0 ± 0.00	125.0 ± 0.00	125.0 ± 0.00	125.0 ± 0.00
*Staphylococcus aureus*	1.95 ± 0.00	7.80 ± 0.00	7.80 ± 0.00	1.95 ± 0.00	7.80 ± 0.00	7.80 ± 0.00
*Staphylococcus epidermidis*	No activity	No activity	15.60 ± 0.00	No activity	No activity	15.60 ± 0.00
*Streptococcus pneumoniae*	31.20 ± 0.00	No activity	15.60 ± 0.00	31.20 ± 0.00	No activity	15.60 ± 0.00

Values are presented as mean ± SD (mg/mL). “No activity” indicates no detectable antibacterial effect under the tested conditions. MIC and MBC were interpreted descriptively because several isolate–season combinations were censored as no activity.

Together, these results show that the antibacterial activity of *A. pannosum* extracts was both season-dependent and isolate-dependent. The complete descriptive values for disc diffusion, well diffusion, MIC, and MBC are provided in Supplementary Table S1, whereas the main text focuses on the principal activity patterns and statistical effects.

### 3.4 Phage–extract compatibility

Prior to the phage–extract interaction experiments, CF01 compatibility with the seasonal *A. pannosum* extracts was evaluated. Following incubation with each extract (100 mg/mL) for 24 h at 37 °C, no detectable reduction in phage infectivity was observed compared with the phage-only control, indicating that the extracts did not adversely affect CF01 stability or plaque-forming ability under the experimental conditions.

### 3.5 Phage–extract interaction against *Escherichia coli* O157:H7

**Figure 4:**
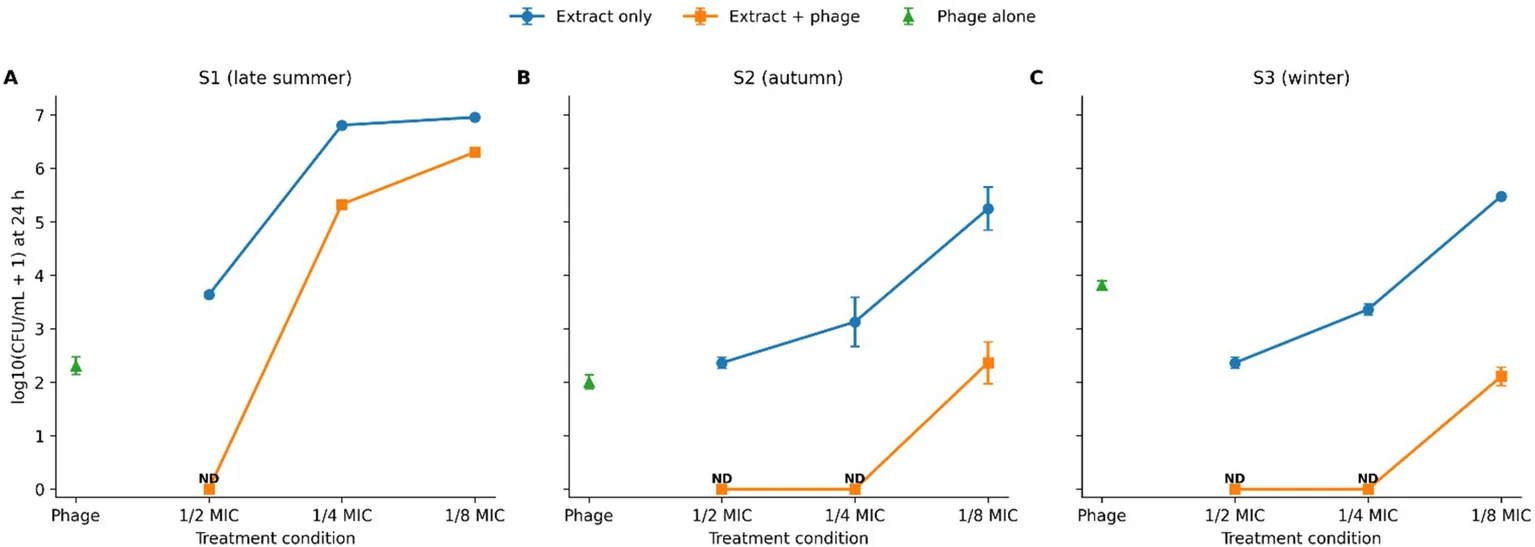
Interaction plots showing the effects of *Abutilon pannosum* extract dose and phage addition on *Escherichia coli* abundance after 24 h across three seasonal collections. Points represent mean log10 (CFU/mL + 1) ± SD, (*n* = 3). S1, late summer (September 2024); S2, autumn (November 2024); and S3, winter (February 2025). ND indicates no detectable colonies in the corresponding treatment group. For *Escherichia coli* O157:H7, the MIC values of S1, S2, and S3 extracts were 125 mg/mL; therefore, 1/2 MIC, 1/4 MIC, and 1/8 MIC corresponded to 62.5, 31.25, and 15.63 mg/mL, respectively.

To determine whether CF01 enhanced the antibacterial effect of seasonal *A. pannosum* extracts, *E. coli* O157:H7 was exposed to sub-MIC extract doses alone and in combination with the phage. Across all three seasonal collections, phage addition reduced bacterial abundance more strongly than the corresponding extract-only treatments, although the magnitude of enhancement depended on both season and extract dose (Figure 4). CF01 alone reduced bacterial abundance compared with untreated controls but did not eliminate detectable bacterial cells after 24 h under the tested conditions.

In S1, extract-only activity increased with dose, and the addition of CF01 further reduced bacterial counts at each tested sub-MIC concentration. The strongest effect was observed at 1/2 MIC + phage, where no recoverable colonies were detected after 24 h. Lower extract doses also benefited from phage addition, although the magnitude of enhancement decreased at 1/4 MIC and 1/8 MIC. In S2 and S3, the phage–extract interaction was more pronounced: combinations at 1/2 MIC and 1/4 MIC reduced *E. coli* to undetectable levels, while 1/8 MIC + phage still produced substantial additional reductions compared with extract alone. These results indicate that the autumn and winter extracts supported stronger CF01-associated bacterial suppression under the tested conditions.

Factorial ANOVA supported the visual patterns, showing significant effects of phage, extract dose, and the phage × dose interaction within each seasonal collection (Table 4). The significant interaction term indicates that the effect of CF01 depended on extract concentration rather than acting as a constant additive effect across all doses. Targeted *post hoc* comparisons further confirmed that, within each season, extract + phage treatments produced significantly greater reductions than the matched extract-only treatments at 1/2 MIC, 1/4 MIC, and 1/8 MIC (Supplementary Table S2).

**Table 4:** Factorial ANOVA of *Escherichia coli* responses to phage, extract dose, and their interaction within each seasonal collection. Response variable: Log10(CFU/mL + 1) at 24 h. Separate 2 × 4 factorial ANOVA models were run for S1, S2, and S3, with factors phage (No/Yes) and extract dose (0, 1/2 MIC, 1/4 MIC, 1/8 MIC).

Season	Effect	df	F	*p*-value	Partial η^2^
S1	Phage	1, 16	9780.05	<0.0001	0.998
S1	Extract dose	3, 16	6612.84	<0.0001	0.999
S1	Phage × Extract dose	3, 16	1249.67	<0.0001	0.996
S2	Phage	1, 16	964.64	<0.0001	0.984
S2	Extract dose	3, 16	231.86	<0.0001	0.978
S2	Phage × Extract dose	3, 16	28.44	<0.0001	0.842
S3	Phage	1, 16	8136.23	<0.0001	0.998
S3	Extract dose	3, 16	3307.26	<0.0001	0.998
S3	Phage × Extract dose	3, 16	49.67	<0.0001	0.903

Overall, these findings demonstrate a positive phage-extract interaction between CF01 and seasonal *A. pannosum* extracts against *E. coli* O157:H7. The most consistent response compatible with enhanced activity under the tested time kill conditions occurred at 1/2 MIC across all seasons, while 1/4 MIC combinations were especially effective in S2 and S3. Conditions in which extract alone already produced complete killing should be interpreted as cases affected by a floor-effect rather than definitive evidence of synergy. Thus, CF01 enhanced the antibacterial performance of *A. pannosum* extracts most clearly at sub-MIC concentrations where extract-only killing was incomplete.

### 3.6 Integrated metabolomics–bioactivity analysis identifies candidate metabolites associated with antibacterial and phage-enhanced activity

To relate seasonal chemical variation to biological performance, the metabolomic and antimicrobial datasets were integrated at two levels: first, by comparing season-level metabolite enrichment with extract-only and phage–extract activity, and second, by exploratory matched-replicate correlation analysis using selected antimicrobial-relevant metabolites. Because only three seasonal collections were available at the season-summary level, this integration was interpreted as descriptive and hypothesis-generating rather than causal. Therefore, citronellol, *α*-linolenic acid, and p-coumaric acid should be considered prioritized candidate metabolites rather than confirmed active principles.

**Figure 5:**
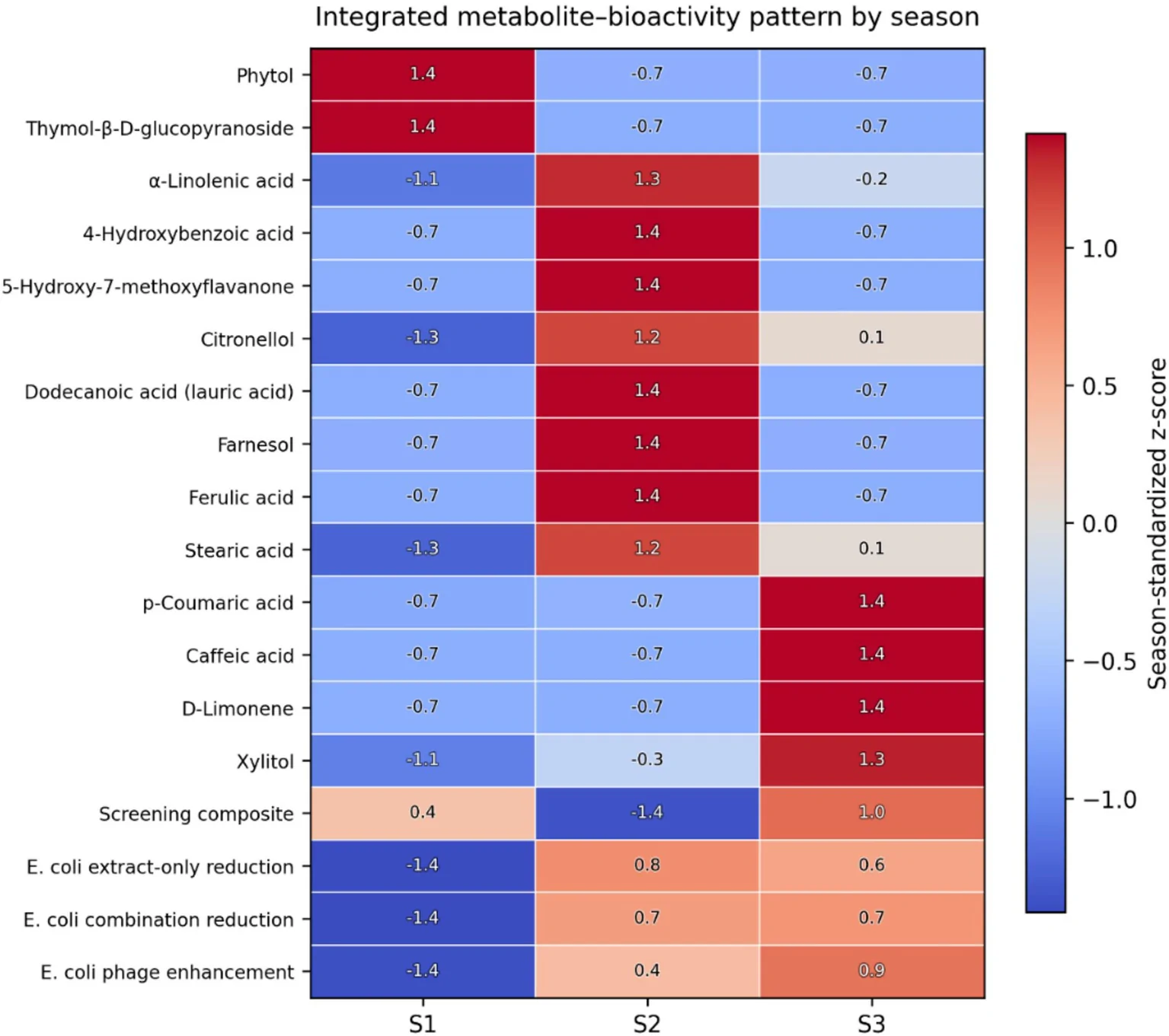
Integrated seasonal pattern of antimicrobial-relevant metabolites and bioactivity in *Abutilon pannosum* extracts. Heatmap values represent season-standardized z-scores for selected metabolites with reported antimicrobial relevance, as well as for aligned seasonal bioactivity metrics, including the screening composite, *Escherichia coli* extract-only reduction, *Escherichia coli* combination reduction, and phage-enhancement magnitude. Positive values indicate relative enrichment or stronger activity within a given season, whereas negative values indicate relative depletion or weaker activity. S1, late summer (September 2024); S2, autumn (November 2024); S3, winter (February 2025). Values represent the mean of three biological replicates (*n* = 3).

The integrated seasonal heatmap showed alignment between metabolite composition and antibacterial performance (Figure 5). The autumn collection (S2) contained the broadest set of literature-supported antibacterial candidates, including *α*-linolenic acid, citronellol, dodecanoic acid, farnesol, ferulic acid, and 4-hydroxybenzoic acid. This profile aligned most clearly with stronger *E. coli* extract-only reduction and combination-associated reduction, although the overall screening composite score was higher in the winter collection (S3). S3 was enriched in caffeic acid, p-coumaric acid, D-limonene, and xylitol and showed the strongest alignment with phage-enhancement patterns. In contrast, the late-summer collection (S1), characterized mainly by phytol and thymol-*β*-D-glucopyranoside among the highlighted metabolites, showed weaker alignment with the *E. coli* extract-only, combination, and phage-enhancement endpoints.

Exploratory matched-replicate correlations further supported this interpretation (Supplementary Figure S5). Citronellol and *α*-linolenic acid showed the strongest positive associations with *E. coli* extract-only reduction (Spearman’s *ρ* = 0.79 and 0.76, respectively), suggesting that these S2-enriched metabolites may be linked to intrinsic extract potency against *E. coli*. For phage-enhancement magnitude, the strongest positive trends were observed for p-coumaric acid, citronellol, and α-linolenic acid (*ρ* = 0.62, 0.61, and 0.59, respectively). In contrast, phytol showed negative associations with both *E. coli* extract-only reduction (*ρ* = −0.86) and phage enhancement (*ρ* = −0.80). These relationships should be interpreted as candidate-prioritization indicators rather than causal evidence. Accordingly, citronellol and α-linolenic acid were prioritized because they showed the strongest positive associations with extract-only *E. coli* reduction and also showed positive trends with phage enhancement, whereas p-coumaric acid showed the strongest positive trend with phage-enhancement magnitude.

The candidate-priority summary (Supplementary Table S4) integrated seasonal enrichment, literature-supported antimicrobial evidence, and descriptive alignment with extract-only and phage-enhanced *E. coli* responses. Overall, S2-enriched metabolites, particularly citronellol and α-linolenic acid, emerged as the strongest candidates associated with *E. coli* extract-only activity, while selected S3-associated metabolites, especially p-coumaric acid, appeared more closely aligned with phage-enhancement patterns. Although xylitol was highly abundant in S3 and was retained among the literature-supported metabolites, it was not classified as a leading candidate because the available evidence indicated weak direct antifungal activity at relatively high concentrations, and it did not rank among the strongest positive associations with the *E. coli* bioactivity endpoints. In addition, because Figure 2 uses row-scaled z-scores, its color intensity represents relative seasonal variation within each metabolite rather than comparative antimicrobial potency among metabolites. These findings support a model in which seasonal shifts in *A. pannosum* chemistry may influence both intrinsic antibacterial activity and the magnitude of the phage–extract interaction. However, because the analysis was based on crude extracts and exploratory associations, the highlighted metabolites should be considered candidate contributors for future validation rather than confirmed causal drivers of activity.

## 4 Discussion

This study shows that the antibacterial potential of *A. pannosum* varies among seasonal collections and is accompanied by clear restructuring of the GC–MS metabolome. The three seasonal extracts differed in their parent-metabolite profiles, extract-only antibacterial activity, and interactions with the lytic *E. coli* phage CF01. The autumn samples were enriched in the broadest set of literature-supported antibacterial candidates, whereas winter samples showed broader extract-only activity across selected screening endpoints and stronger alignment with phage-enhancement patterns. The phage–extract assays further showed that CF01 improved *E. coli* O157:H7 suppression at sub-MIC extract concentrations, especially where extract-only killing was incomplete. The mechanisms underlying the positive phage-extract interaction remain to be determined. One possibility is that sub-MIC extract exposure increases bacterial membrane permeability, alters stress-response pathways, affects phage receptor accessibility, or reduces bacterial recovery after phage infection. These explanations remain hypothetical and require validation through phage adsorption, burst-size, and bacterial physiology assays. Together, these findings support a model in which seasonal chemistry influences both intrinsic extract activity and compatibility with phage-assisted bacterial suppression, while the candidate metabolites identified here remain priorities for future validation rather than confirmed causal drivers.

### 4.1 Seasonal metabolome restructuring and antimicrobial-relevant chemistry

The seasonal restructuring observed in *A. pannosum* is biologically relevant because it altered the abundance of metabolites with reported antimicrobial activity, rather than merely changing the overall chemical profile. The autumn collection (S2) contained the broadest set of literature-supported antibacterial candidates, including α-linolenic acid, citronellol, dodecanoic acid, farnesol, ferulic acid, and 4-hydroxybenzoic acid. In contrast, the winter collection (S3) was characterized by caffeic acid, *p*-coumaric acid, D-limonene, and xylitol, whereas the late-summer collection (S1) was mainly associated with phytol and thymol-β-D-glucopyranoside among the highlighted antimicrobial-relevant metabolites. Such seasonal redistribution is consistent with previous evidence that medicinal-plant chemistry and biological activity can vary with season, water availability, and environmental conditions (Prinsloo and Nogemane, 2018; Ncube et al., 2011; Ferraz et al., 2018).

The S2-enriched profile is notable because fatty acids, terpenoid alcohols, and phenolic acids have all been linked to antibacterial activity through mechanisms such as membrane disruption, growth inhibition, or antibiofilm effects, although potency varies strongly with compound, strain, and assay format (Lee et al., 2002; Casillas-Vargas et al., 2021; Lopez-Romero et al., 2015; Jabra-Rizk et al., 2006; Borges et al., 2013; Kępa et al., 2018). However, these compounds should be considered candidate contributors rather than confirmed causal agents, as the present study used crude extracts and did not isolate or test individual metabolites. This distinction is important because S2 showed the broadest antimicrobial-candidate enrichment, whereas S3 aligned more strongly with selected screening and phage-enhancement endpoints, suggesting that different seasonal extracts may support different antibacterial functions.

### 4.2 Season-dependent antibacterial activity of the extracts

The antibacterial assays showed that seasonal changes in *A. pannosum* chemistry translated into an isolate-dependent antibacterial phenotype. Across the diffusion and dilution-based assays, *S. aureus* was the most susceptible bacterium, whereas *E. coli* showed weaker direct susceptibility in extract-only screening. This pattern is consistent with many plant-extract studies in which Gram-positive bacteria are often more responsive than Gram-negative bacteria, partly because the outer membrane of Gram-negative bacteria can restrict the penetration of hydrophobic or moderately polar phytochemicals (Jubair et al., 2021; Zouine et al., 2024). However, the weaker direct response of *E. coli* should be interpreted as relative rather than absolute, because measurable inhibition was observed in selected assays and stronger suppression occurred later in the phage–extract interaction experiment. It should also be noted that the MIC value of the crude *A. pannosum* extract against *E. coli* O157:H7 was relatively high (125 mg/mL), indicating weak intrinsic antibacterial activity when the extract was used alone. This finding is not unexpected for an unfractionated crude plant extract, in which individual bioactive compounds are present at relatively low concentrations and may act through additive or complementary effects rather than as highly potent single agents. Importantly, despite this relatively weak direct antibacterial activity, the extract markedly enhanced CF01-mediated bacterial suppression at sub-MIC concentrations, suggesting that its principal value may lie in supporting phage-assisted antibacterial activity rather than serving as a potent antibacterial agent on its own.

The enhanced antibacterial effect observed during the phage–extract interaction may be explained by several possible mechanisms. Exposure to sub-inhibitory concentrations of plant-derived metabolites could increase bacterial membrane permeability, modify physiological stress responses, alter the accessibility or expression of phage receptors, or reduce bacterial recovery following phage-mediated damage. Such changes may increase bacterial susceptibility to CF01 infection and contribute to the improved bacterial suppression observed with combined treatments. However, these mechanisms remain hypothetical, and further experiments assessing membrane integrity, receptor changes, phage adsorption, and replication dynamics are required to confirm the underlying basis of the extract–phage interaction.

The seasonal pattern was also not uniform across isolates. The S2 extract was chemically enriched in several antimicrobial-relevant metabolites but showed no detectable diffusion-based activity against some isolates, whereas the S3 extract showed broader measurable activity in the screening assays, including against *S. epidermidis*. This difference suggests that extract activity was not determined only by the number of literature-supported antibacterial metabolites present, but also by metabolite availability, diffusion behavior, target-organism susceptibility, and possible interactions among extract constituents. Similar season-dependent variation in antimicrobial activity has been reported in medicinal plants, where changes in phenolic, terpenoid, and lipid-associated constituents alter extract potency across collection periods (Ncube et al., 2011; Ferraz et al., 2018; Prinsloo and Nogemane, 2018). Therefore, the present data support the interpretation that *A. pannosum* has a season-dependent and isolate-dependent antibacterial profile, with collection timing influencing both chemical composition and measurable bioactivity.

### 4.3 Positive phage–extract interaction against *Escherichia coli* O157:H7

A distinctive finding of this study was that seasonal *A. pannosum* extracts remained compatible with CF01 and enhanced *suppression of E. coli O157:H7* when combined with the phage. This is microbiologically important because plant–phage combinations remain much less studied than phage–antibiotic combinations; published evidence shows that botanical extracts can enhance or interfere with phage performance depending on extract chemistry, bacterial host physiology, and the phage system used (Stachurska et al., 2023; Mirzaei et al., 2024), while phage–antibiotic studies further show that combination effects can range from synergistic to antagonistic depending on mechanism and stoichiometry (Liu et al., 2020; Qin et al., 2024; Kunz Coyne et al., 2024). In the present study, the strongest phage-enhanced responses occurred at sub-MIC extract concentrations, particularly at 1/2 MIC across all seasons and at 1/4 MIC in the autumn and winter collections. This pattern indicates that CF01 provided the greatest added benefit when extract-only killing was incomplete, which is the most informative condition for evaluating phage–extract compatibility.

The interaction should be interpreted as synergy-consistent rather than mechanistically proven synergy. Although the combination produced a significantly greater reduction in bacteria than extract-only treatments, the study did not directly test mechanisms such as phage adsorption, burst size, bacterial membrane permeability, or intracellular phage replication in the presence of the extract. Therefore, the most defensible interpretation is that selected seasonal extracts created conditions that favored stronger phage-assisted suppression, rather than proving a specific molecular mechanism. This conclusion is consistent with the broader phage-combination literature, in which enhanced killing may arise from complementary stress pathways, altered bacterial susceptibility, or changes in population dynamics rather than a single, universal synergy mechanism (Liu et al., 2020; Kunz Coyne et al., 2024). The use of CF01, a previously characterized lytic *E. coli* phage, further strengthens the biological basis of the assay, as the phage had already been characterized before being used as a standardized interaction partner (Shaaban et al., 2026).

### 4.4 Integrated metabolomics–bioactivity interpretation and candidate prioritization

The integrated analysis helped distinguish metabolites associated with direct extract activity from those more closely aligned with phage-enhanced suppression. Citronellol and *α*-linolenic acid showed the strongest positive associations with *E. coli* extract-only reduction, whereas *p*-coumaric acid, citronellol, and α-linolenic acid showed the strongest positive trends with phage-enhancement magnitude. This pattern suggests partial overlap between metabolites associated with intrinsic extract potency and those associated with improved phage-assisted activity. Such integration is valuable because crude plant extracts contain multiple interacting metabolites, and bioactivity-guided metabolomics can help prioritize candidate compounds for validation rather than relying only on inhibition-zone or MIC results (Porras et al., 2020; Adeosun and Prinsloo, 2025).

Among the prioritized candidates, citronellol and α-linolenic acid are particularly relevant because they were enriched in S2 and showed positive alignment with *E. coli*-related endpoints. Citronellol and related monoterpenoid alcohols have been reported to affect bacterial growth and membrane-associated responses, while α-linolenic acid and other unsaturated fatty acids have been linked to antibacterial effects through membrane disruption and related physiological stress (Lee et al., 2002; Lopez-Romero et al., 2015; Casillas-Vargas et al., 2021). The S3-associated *p*-coumaric acid may be more relevant to the phage-enhancement pattern, although its role remains inferential. Phenolic acids, including caffeic, ferulic, and *p*-coumaric acids, have been reported to exhibit antibacterial activity, but their effects vary with microbial strain, concentration, and assay conditions (Borges et al., 2013; Lou et al., 2012; Kępa et al., 2018). Therefore, these metabolites should be treated as candidate contributors rather than confirmed active principles. Direct validation using purified compounds, defined mixtures, and phage-performance assays will be needed to determine whether they enhance bacterial susceptibility, support phage action, or simply co-vary with more active chemical fractions.

### 4.5 Strengths, limitations, and future directions

A major strength of this study is its integrated design, which combined seasonal GC–MS profiling, extract-only antibacterial assays, phage–extract interaction testing, and metabolomics–bioactivity alignment within a single experimental framework. This allowed seasonal differences in *A. pannosum* chemistry to be interpreted alongside microbiological performance rather than as isolated phytochemical variation. The use of the previously characterized lytic phage CF01 also strengthened the phage-interaction component, as the biological agent was already defined by morphology, genomic features, and functional behavior before application in the present extract-combination assay (Shaaban et al., 2026). More broadly, this framework aligns with recent efforts to combine natural-product profiling with bioassays to prioritize candidate antimicrobial extracts and metabolites for further validation (Porras et al., 2020; El-Keblawy, Sallam et al., 2026). From an applied perspective, these findings suggest that the collection season may influence the reproducibility and antibacterial performance of desert-plant extracts. Seasonal selection of plant material may therefore be important for future antimicrobial screening and for the development of plant extract phage adjunct strategies.

An important limitation of the present study is the absence of a spring collection. Although *Abutilon pannosum* is a perennial species that flowers during spring, logistical constraints during the study period prevented spring sampling. Consequently, the seasonal comparison was restricted to late summer, autumn, and winter and does not represent a complete annual cycle. Future studies incorporating spring collections are needed to provide a more comprehensive assessment of seasonal phytochemical variation and its potential influence on antibacterial activity and phage–extract interactions. Although the present study demonstrated seasonal variation in the metabolomic profile and antibacterial activity of *A. pannosum*, the three seasonal collections were conducted within a single year and from a single geographical location. Therefore, seasonal effects may be partially confounded by year-specific environmental conditions and local ecological factors. In addition, environmental variables such as temperature, rainfall, soil characteristics, and plant developmental stage were not systematically recorded during sampling. These factors may also have contributed to the observed metabolomic differences and should be incorporated into future studies to better distinguish seasonal from environmental influences. Future studies involving multi-year sampling and multiple collection sites would help further clarify the relative contribution of seasonal and environmental factors to the phytochemical composition and biological activity of this species. First, the antimicrobial activity was evaluated using crude extracts, so the contribution of individual metabolites remains inferential. Second, metabolite identifications were based on GC–MS library matching and should be considered putative unless confirmed by authentic standards, retention indices, or targeted MS/MS approaches (Sumner et al., 2007). Third, although the phage–extract combinations showed enhanced antibacterial effects, the mechanisms underlying this interaction were not directly tested; therefore, the findings should be interpreted as phage-enhanced or synergy-consistent bacterial suppression rather than as mechanistically proven synergy. Future experiments should include extract–phage controls in the absence of bacteria to determine whether seasonal extracts directly affect CF01 stability or infectivity. Finally, the integration between metabolomics and bioactivity was exploratory because only three seasonal collections were available, and cytotoxicity, formulation stability, and *in vivo* or food-related model validation were beyond the scope of the present study. In addition, supervised multivariate analyses, such as partial least squares-discriminant analysis (PLS-DA), orthogonal partial least squares-discriminant analysis (OPLS-DA), and variable importance in projection (VIP) score analysis, were not performed. Future studies with larger datasets should incorporate these approaches to further validate season-specific biomarkers and improve metabolite discrimination. Accordingly, the highlighted metabolites should be interpreted as prioritized candidates for future validation rather than confirmed causal drivers of the observed antibacterial or phage-enhancing effects.

Although the mechanisms underlying the enhanced antibacterial activity of the phage–extract combinations were not investigated in the present study, seasonal differences in metabolite composition may have influenced bacterial susceptibility to phage-mediated killing. However, this remains a hypothesis and should be interpreted with caution until confirmed by dedicated mechanistic studies.

Future work should therefore focus on bioassay-guided fractionation and validation of the prioritized candidate metabolites, particularly citronellol, *α*-linolenic acid, and *p*-coumaric acid. The associations proposed in the present study are based on seasonal metabolomic patterns, exploratory correlation analysis, and previously reported antimicrobial properties, but were not supported by experimental validation using purified compounds. Testing purified compounds individually and in defined combinations with CF01 would help determine whether these metabolites directly contribute to antibacterial activity, enhance bacterial susceptibility to phage infection, improve phage performance, or simply serve as markers of more active seasonal extract profiles. Additional mechanistic studies should examine phage adsorption kinetics, burst size, bacterial membrane permeability, and resistance emergence to clarify the mechanisms underlying extract–phage interactions. Furthermore, cytotoxicity assessment, formulation stability studies, and validation in relevant food matrices and in vivo models will be essential to evaluate the practical applicability, safety, and therapeutic potential of seasonal *A. pannosum* extracts and the identified candidate bioactive metabolites as antibacterial agents or phage-adjunct candidates.

## 5 Conclusion

This study demonstrates that the antibacterial potential of *A. pannosum* is season-dependent and linked to clear restructuring of its GC–MS metabolome. Across the three seasonal collections, the autumn extract contained the broadest set of literature-supported antibacterial candidates, whereas the winter extract showed stronger alignment with selected antibacterial screening and phage-enhancement endpoints. Extract-only antibacterial activity varied by both season and bacterial isolate, with *S. aureus* showing the greatest overall susceptibility and *E. coli* showing weaker direct susceptibility in screening assays.

The phage–extract interaction assay showed that CF01 enhanced suppression of *E. coli* O157:H7 at sub-MIC extract concentrations, particularly where extract-only killing was incomplete. This supports the compatibility of selected seasonal *A. pannosum* extracts with lytic phage treatment and highlights the value of evaluating plant extracts not only as direct antimicrobials but also as potential phage-adjunct candidates. Integrated metabolomics–bioactivity analysis prioritized citronellol, *α*-linolenic acid, and *p*-coumaric acid as candidate metabolites associated with extract potency and/or phage-enhanced activity. However, these metabolites should be considered candidate contributors rather than confirmed causal agents until validated through purified-compound testing, defined mixtures, and mechanistic phage-performance assays.

Overall, the findings support a seasonally informed approach to antibacterial screening of desert medicinal plants and demonstrate that combining metabolomics, extract bioassays, and phage–extract interaction testing can provide a useful framework for prioritizing bioactive extracts and candidate metabolites for future antimicrobial validation.

## Data Availability

The nucleotide sequence of bacteriophage CF01 has been deposited in GenBank under accession number PX441839.1. The complete processed parent-metabolite dataset is provided in Supplementary Table S3, while the replicate-level retained-feature matrix and derivatized-feature-to-parent-metabolite mapping are provided in Supplementary material S1. The remaining data supporting the findings of this study are included in the article and its Supplementary material. Further inquiries may be directed to the corresponding authors.
