# Supplementary material for: Seasonal metabolomics and antibacterial activity of Abutilon pannosum reveal positive interaction with the lytic phage CF01 against Escherichia coli O157:H7 (strain 161–84)
Source: Front Microbiol. 2026 Aug 21;17:1916998. doi: 10.3389/fmicb.2026.1916998 (PMC13552351; doi:10.3389/fmicb.2026.1916998)
Supplement: Supplementary file 1 [file Data_Sheet_1.pdf]

Supplementary Table S1. Mean ± SD values used to construct Figure 3 for disc diffusion, well diffusion, MIC, and MBC assays across seasonal collections. For disc diffusion and well diffusion, different superscript letters within a row indicate significant differences among seasonal collections based on Tukey's post hoc test ($p < 0.05$). MIC and MBC values are presented descriptively without post hoc lettering.

Assay	Isolate	S1	S2	S3
Disc diffusion	*E. coli*	6.3 ± 1.5 a	0.0 ± 0.0 b	7.0 ± 1.0 a
	S. aureus	16.0 ± 1.0 a	15.7 ± 0.6 a	14.3 ± 0.6 a
	S. epidermidis	0.0 ± 0.0 b	0.0 ± 0.0 b	10.7 ± 1.2 a
	S. pneumoniae	9.7 ± 0.6 a	0.0 ± 0.0 b	8.3 ± 1.5 a
Well diffusion	*E. coli*	11.7 ± 1.5 a	0.0 ± 0.0 b	13.7 ± 1.5 a
	S. aureus	16.0 ± 1.0 b	17.0 ± 1.0 b	20.7 ± 1.2 a
	S. epidermidis	0.0 ± 0.0 b	0.0 ± 0.0 b	14.3 ± 0.6 a
	S. pneumoniae	14.0 ± 1.0 a	0.0 ± 0.0 b	13.3 ± 1.2 a
MIC	*E. coli*	125.00 ± 0.00	No activity	125.00 ± 0.00
	S. aureus	1.95 ± 0.00	7.80 ± 0.00	7.80 ± 0.00
	S. epidermidis	No activity	No activity	15.60 ± 0.00
	S. pneumoniae	31.20 ± 0.00	No activity	15.60 ± 0.00
MBC	*E. coli*	125.00 ± 0.00	No activity	125.00 ± 0.00
	S. aureus	1.95 ± 0.00	7.80 ± 0.00	7.80 ± 0.00
	S. epidermidis	No activity	No activity	15.60 ± 0.00
	S. pneumoniae	31.20 ± 0.00	No activity	15.60 ± 0.00

Note. Diffusion values are shown in mm; MIC and MBC values are shown in mg/mL.

Supplementary Table S2. Simple targeted comparisons of extract-only versus extract + phage treatments at each dose within each seasonal collection. Comparisons were performed on log10(CFU/mL + 1) at 24 h. Holm-adjusted p-values are reported for each season and each of the three tested doses.

Season	Dose	Extract only mean	Extract + phage mean	Mean difference	t	Holm-adjusted p	Significant
S1	1/2 MIC	3.634	0.000	3.634	112.54	<0.0001	Yes
S1	1/4 MIC	6.808	5.327	1.480	44.90	<0.0001	Yes
S1	1/8 MIC	6.954	6.301	0.653	38.2	<0.0001	Yes
S2	1/2 MIC	2.362	0.000	2.362	40.40	<0.0001	Yes
S2	1/4 MIC	3.127	0.000	3.127	11.72	<0.001	Yes
S2	1/8 MIC	5.244	2.362	2.882	8.89	<0.001	Yes
S3	1/2 MIC	2.362	0.000	2.362	40.40	<0.0001	Yes
S3	1/4 MIC	3.360	0.000	3.360	57.27	<0.0001	Yes
S3	1/8 MIC	5.477	2.104	3.373	33.86	<0.0001	Yes

Abbreviations: MIC, minimum inhibitory concentration; CFU, colony-forming units; S1, late summer (September 2024); S2, autumn (November 2024); S3, winter (February 2025). ND groups in the figure correspond to no detectable colonies.

Supplementary Table S3. Complete processed GC–MS parent-metabolite dataset of seasonal *Abutilon pannosum* leaf extracts.

Dataset scope. The table lists all 350 consolidated parent metabolites used for seasonal comparison. All compound names are putative library-based annotations; none was confirmed using an authentic standard.

Seasonal groups. S1 = late summer (September 2024), S2 = autumn (November 2024), and S3 = winter (February 2025).

Peak-intensity reporting. Values are raw integrated GC–MS peak areas in arbitrary units and are presented as mean ± SD for three biological replicates. Zeros were retained and indicate that no peak area was recorded in the supplied processed matrix.

Parent-metabolite consolidation. When multiple retained analytical features mapped to the same parent metabolite, their replicate-level peak areas were summed before the seasonal mean, SD, and detection frequency were calculated.

Display precision. Peak areas are rounded to three significant digits in this Word table. Exact replicate-level values, retained-feature records, and derivatized-feature mappings are provided in Supplementary Data S1 (Excel).

Interpretation. "Dominant season" identifies the collection with the highest mean peak area and is descriptive; it does not indicate statistical significance. No retained entry in the supplied processed export was labelled as unidentified.

Parent ID	Putative parent metabolite	Features (n)	Contributing retained feature form(s)	S1 mean ± SD (peak area)	S1 detected (n/3)	S2 mean ± SD (peak area)	S2 detected (n/3)	S3 mean ± SD (peak area)	S3 detected (n/3)	Dominant season
PM001	(3-Hydroxy-4-methoxyphenyl)ethylene glycol	1	(3-Hydroxy-4-methoxyphenyl)ethylene glycol	$3.61 \times 10^7 \pm 7.08 \times 10^5$	3/3	0	0/3	$3.66 \times 10^7 \pm 6.35 \times 10^7$	1/3	S3
PM002	(R*,S*)-2,3-Dihydroxybutanoic acid	1	(R*,S*)-2,3-Dihydroxybutanoic acid	0	0/3	$1.14 \times 10^6 \pm 9.92 \times 10^5$	2/3	$5.58 \times 10^5 \pm 9.66 \times 10^5$	1/3	S2
PM003	1,2,3-Butanetriol	1	1,2,3-Butanetriol	$1.6 \times 10^6 \pm 5.75 \times 10^5$	3/3	$4.23 \times 10^5 \pm 7.32 \times 10^5$	1/3	$3.86 \times 10^5 \pm 6.68 \times 10^5$	1/3	S1
PM004	1,2,4-Butanetriol	1	1,2,4-Butanetriol	$1.67 \times 10^6 \pm 2.63 \times 10^6$	2/3	$4.06 \times 10^6 \pm 7.03 \times 10^6$	1/3	$4.65 \times 10^6 \pm 1.39 \times 10^6$	3/3	S3
PM005	1,2,5,6-Hexanetetrol	1	1,2,5,6-Hexanetetrol	0	0/3	0	0/3	$9.16 \times 10^5 \pm 1.59 \times 10^6$	1/3	S3
PM006	1,2-Ethanediamine, N-propyl-	1	1,2-Ethanediamine, N-propyl-	$3.14 \times 10^7 \pm 2.14 \times 10^6$	3/3	0	0/3	$3.16 \times 10^7 \pm 7.56 \times 10^6$	3/3	S3
PM007	1,2-Ethenediol	1	1,2-Ethenediol	$1.91 \times 10^5 \pm 1.66 \times 10^5$	2/3	0	0/3	0	0/3	S1
PM008	1,6,10,14,18,22-Tetracosahexaen-3-ol, 2,6,10,15,19,23-hexamethyl-, (all-E)-(.+/-.)-	1	1,6,10,14,18,22-Tetracosahexaen-3-ol, 2,6,10,15,19,23-hexamethyl-, (all-E)-(.+/-.)-	0	0/3	$3.64 \times 10^8 \pm 1.64 \times 10^7$	3/3	0	0/3	S2
PM009	1-(2-Methoxy-3-methylphenyl)-N-methylpropan-2-amine	1	1-(2-Methoxy-3-methylphenyl)-N-methylpropan-2-amine	0	0/3	0	0/3	$7.48 \times 10^4 \pm 1.3 \times 10^5$	1/3	S3
PM010	1-Ethyl-2-dimethyl(octyl)oxycyclohexane	1	1-Ethyl-2-dimethyl(octyl)oxycyclohexane	$1.03 \times 10^6 \pm 1.79 \times 10^6$	1/3	$2.04 \times 10^7 \pm 4.14 \times 10^6$	3/3	0	0/3	S2
PM011	1-Heneicosanol	1	1-Heneicosanol	0	0/3	$3.87 \times 10^7 \pm 3.44 \times 10^6$	3/3	0	0/3	S2
PM012	1-Hexanol, 5-methyl-2-(1-methylethyl)-, acetate	1	1-Hexanol, 5-methyl-2-(1-methylethyl)-, acetate	$4.4 \times 10^5 \pm 7.63 \times 10^5$	1/3	0	0/3	0	0/3	S1
PM013	1-Linolenoylglycerol	1	1-Linolenoylglycerol	$3.74 \times 10^7 \pm 1.2 \times 10^7$	3/3	$2.55 \times 10^8 \pm 1.04 \times 10^7$	3/3	$7.92 \times 10^7 \pm 1.01 \times 10^7$	3/3	S2

Parent ID	Putative parent metabolite	Features (n)	Contributing retained feature form(s)	S1 mean ± SD (peak area)	S1 detected (n/3)	S2 mean ± SD (peak area)	S2 detected (n/3)	S3 mean ± SD (peak area)	S3 detected (n/3)	Dominant season
PM014	1-Methyl-2-pentamethyloxycyclohexane	1	1-Methyl-2-pentamethyloxycyclohexane	$4.33 \times 10^3 \pm 7.49 \times 10^3$	1/3	0	0/3	0	0/3	S1
PM015	1-Monolinolein	1	1-Monolinolein	0	0/3	$1.32 \times 10^8 \pm 1.08 \times 10^7$	3/3	$5.67 \times 10^7 \pm 8.45 \times 10^6$	3/3	S2
PM016	1-Monopalmitin	1	1-Monopalmitin	$2.93 \times 10^8 \pm 6.21 \times 10^6$	3/3	$3.95 \times 10^8 \pm 2.12 \times 10^7$	3/3	$1.55 \times 10^8 \pm 1.83 \times 10^7$	3/3	S2
PM017	1-O-trans-p-Coumaroylglycerol	1	1-O-trans-p-Coumaroylglycerol	$3.51 \times 10^7 \pm 4.13 \times 10^6$	3/3	0	0/3	$6.55 \times 10^6 \pm 7.09 \times 10^5$	3/3	S1
PM018	1-Octacosanol	1	1-Octacosanol	$1.1 \times 10^7 \pm 9.59 \times 10^6$	2/3	$2.17 \times 10^7 \pm 1.63 \times 10^6$	3/3	$2.07 \times 10^7 \pm 2.45 \times 10^6$	3/3	S2
PM019	1-Octanol, 2,2-dimethyl-	1	1-Octanol, 2,2-dimethyl-	0	0/3	$4.09 \times 10^5 \pm 7.09 \times 10^5$	1/3	$4.22 \times 10^5 \pm 7.3 \times 10^5$	1/3	S3
PM020	1-Pentamethyloxycyclohexane	1	1-Pentamethyloxycyclohexane	$5.2 \times 10^3 \pm 9.01 \times 10^3$	1/3	0	0/3	0	0/3	S1
PM021	1-Propene, 1-methoxy-2-methyl-1-[(dimethyltert-)oxy]-	1	1-Propene, 1-methoxy-2-methyl-1-[(dimethyltert-)oxy]-	$1.25 \times 10^5 \pm 2.16 \times 10^5$	1/3	$9.27 \times 10^5 \pm 8.18 \times 10^5$	2/3	0	0/3	S2
PM022	1-Propene-1-thiol	1	1-Propene-1-thiol	0	0/3	$4.72 \times 10^7 \pm 4.4 \times 10^7$	2/3	0	0/3	S2
PM023	1-Triacontanol	1	1-Triacontanol	0	0/3	0	0/3	$4.74 \times 10^5 \pm 1.24 \times 10^5$	3/3	S3
PM024	11-Methylpentacosane	1	11-Methylpentacosane	0	0/3	0	0/3	$3.91 \times 10^4 \pm 6.77 \times 10^4$	1/3	S3
PM025	12-Methyloctadec-11-enoic acid	1	12-Methyloctadec-11-enoic acid	0	0/3	$1.43 \times 10^6 \pm 2.48 \times 10^6$	1/3	0	0/3	S2
PM026	16-Methyl-heptadecane-1,2-diol	1	16-Methyl-heptadecane-1,2-diol	$1.62 \times 10^7 \pm 1.01 \times 10^6$	3/3	0	0/3	0	0/3	S1
PM027	17-Octadecynoic acid	1	17-Octadecynoic acid	0	0/3	$1.47 \times 10^6 \pm 2.55 \times 10^6$	1/3	$2.31 \times 10^6 \pm 2.02 \times 10^6$	2/3	S3
PM028	2,2-Dimethyl-3-oxobutyric acid	1	2,2-Dimethyl-3-oxobutyric acid	0	0/3	$2.29 \times 10^5 \pm 3.97 \times 10^5$	1/3	0	0/3	S2
PM029	2,3,4,5-Tetrahydroxypentanoic acid-1,4-lactone	1	2,3,4,5-Tetrahydroxypentanoic acid-1,4-lactone	0	0/3	$9.79 \times 10^5 \pm 1.69 \times 10^6$	1/3	0	0/3	S2
PM030	2,3,4-Trihydroxybutyric acid, (, (R*,R*)-)	1	2,3,4-Trihydroxybutyric acid, (, (R*,R*)-)	$6.08 \times 10^7 \pm 3.72 \times 10^7$	3/3	$6.28 \times 10^7 \pm 2.97 \times 10^7$	3/3	$1.05 \times 10^7 \pm 3.11 \times 10^6$	3/3	S2
PM031	2,3,4-Trimethyl-3-hydroxyglutaric acid	1	2,3,4-Trimethyl-3-hydroxyglutaric acid	0	0/3	$9 \times 10^5 \pm 8.05 \times 10^5$	2/3	0	0/3	S2
PM032	2,3-Bis(oxy)propyl docosanoate	1	2,3-Bis(oxy)propyl docosanoate	0	0/3	$1.74 \times 10^6 \pm 1.51 \times 10^6$	2/3	$2.18 \times 10^6 \pm 3.09 \times 10^5$	3/3	S3
PM033	2,3-Butanediol	1	2,3-Butanediol	$4.83 \times 10^5 \pm 4.41 \times 10^5$	2/3	0	0/3	0	0/3	S1

Parent ID	Putative parent metabolite	Features (n)	Contributing retained feature form(s)	S1 mean ± SD (peak area)	S1 detected (n/3)	S2 mean ± SD (peak area)	S2 detected (n/3)	S3 mean ± SD (peak area)	S3 detected (n/3)	Dominant season
PM034	2,3-Dihydroxy-2-methylpropanoic acid	1	2,3-Dihydroxy-2-methylpropanoic acid	$4.49 \times 10^{7} \pm 7.77 \times 10^{7}$	1/3	0	0/3	0	0/3	S1
PM035	2,5-Monomethylene-l-rhamnitol	1	2,5-Monomethylene-l-rhamnitol	$2.73 \times 10^{5} \pm 4.73 \times 10^{5}$	1/3	0	0/3	0	0/3	S1
PM036	2-[oxy]-2-{4-[oxy]phenyl}ethanamine	1	2-[oxy]-2-{4-[oxy]phenyl}ethanamine	0	0/3	$6.73 \times 10^{6} \pm 1.17 \times 10^{7}$	1/3	0	0/3	S2
PM037	2-[oxy]tetradecanoic acid	1	2-[oxy]tetradecanoic acid	0	0/3	$5.85 \times 10^{6} \pm 1.01 \times 10^{7}$	1/3	0	0/3	S2
PM038	2-alpha-Mannobiose	1	2-.alpha.-Mannobiose, methyloxime (isomer 2)	0	0/3	$5.67 \times 10^{6} \pm 6.33 \times 10^{6}$	2/3	$2.65 \times 10^{7} \pm 4.59 \times 10^{7}$	1/3	S3
PM039	2-alpha-Mannobiose, (isomer 1)	1	2-.alpha.-Mannobiose, (isomer 1)	$1.42 \times 10^{7} \pm 7.47 \times 10^{6}$	3/3	$4.33 \times 10^{6} \pm 7.49 \times 10^{6}$	1/3	$5.89 \times 10^{7} \pm 3.01 \times 10^{6}$	3/3	S3
PM040	2-alpha-Mannobiose, (isomer 2)	1	2-.alpha.-Mannobiose, (isomer 2)	0	0/3	0	0/3	$2.5 \times 10^{5} \pm 4.33 \times 10^{5}$	1/3	S3
PM041	2-Aminobutanoic acid	1	2-Aminobutanoic acid	$7.39 \times 10^{6} \pm 1.28 \times 10^{7}$	1/3	$8.41 \times 10^{5} \pm 1.46 \times 10^{6}$	1/3	0	0/3	S1
PM042	2-Butene-1,4-diol	1	2-Butene-1,4-diol	$3.65 \times 10^{6} \pm 6.32 \times 10^{6}$	1/3	$2.19 \times 10^{7} \pm 3.76 \times 10^{7}$	2/3	0	0/3	S2
PM043	2-Butene-1,4-diol, (Z)-	1	2-Butene-1,4-diol, (Z)-	$2.44 \times 10^{5} \pm 4.22 \times 10^{5}$	1/3	$2.76 \times 10^{7} \pm 4.78 \times 10^{7}$	1/3	$4.87 \times 10^{7} \pm 8.43 \times 10^{7}$	1/3	S3
PM044	2-Butenedioic acid, (E)-	1	2-Butenedioic acid, (E)-	$2.82 \times 10^{8} \pm 2.99 \times 10^{7}$	3/3	$5.4 \times 10^{7} \pm 1.45 \times 10^{7}$	3/3	$9.45 \times 10^{7} \pm 1.93 \times 10^{7}$	3/3	S1
PM045	2-Butenedioic acid, (Z)-	1	2-Butenedioic acid, (Z)-	$1.3 \times 10^{7} \pm 2.22 \times 10^{7}$	2/3	$1.25 \times 10^{6} \pm 2.17 \times 10^{6}$	1/3	0	0/3	S1
PM046	2-Butenoic acid, 3-methyl-	1	2-Butenoic acid, 3-methyl-	$9.73 \times 10^{6} \pm 8.59 \times 10^{6}$	2/3	$5.85 \times 10^{5} \pm 1.01 \times 10^{6}$	1/3	$1.92 \times 10^{6} \pm 1.79 \times 10^{6}$	2/3	S1
PM047	2-Cyclopropylethenol	1	2-Cyclopropylethenol	0	0/3	$3.7 \times 10^{5} \pm 6.41 \times 10^{5}$	1/3	0	0/3	S2
PM048	2-Deoxy-D-ribose, (isomer 1)	1	2-Deoxy-D-ribose, (isomer 1)	0	0/3	$1.04 \times 10^{7} \pm 2.38 \times 10^{6}$	3/3	0	0/3	S2
PM049	2-Deoxypentofuranose	1	2-Deoxypentofuranose	$4.86 \times 10^{5} \pm 8.42 \times 10^{5}$	1/3	0	0/3	0	0/3	S1
PM050	2-Hexen-1-ol, (Z)-	1	2-Hexen-1-ol, (Z)-	$1.68 \times 10^{6} \pm 1.47 \times 10^{6}$	2/3	0	0/3	0	0/3	S1
PM051	2-Hexenoic acid, (E)-	1	2-Hexenoic acid, (E)-	$2.46 \times 10^{6} \pm 1.62 \times 10^{6}$	3/3	$1.74 \times 10^{6} \pm 8.2 \times 10^{5}$	3/3	$1.45 \times 10^{7} \pm 5.86 \times 10^{6}$	3/3	S3
PM052	2-Hexenoic acid, 5-(1-ethoxyethoxy)-	1	2-Hexenoic acid, 5-(1-ethoxyethoxy)-	0	0/3	$3.34 \times 10^{5} \pm 5.78 \times 10^{5}$	1/3	$3.74 \times 10^{5} \pm 6.48 \times 10^{5}$	1/3	S3
PM053	2-Hydroxyoctanoic acid	1	2-Hydroxyoctanoic acid	0	0/3	$4.9 \times 10^{5} \pm 8.49 \times 10^{5}$	1/3	0	0/3	S2

Parent ID	Putative parent metabolite	Features (n)	Contributing retained feature form(s)	S1 mean ± SD (peak area)	S1 detected (n/3)	S2 mean ± SD (peak area)	S2 detected (n/3)	S3 mean ± SD (peak area)	S3 detected (n/3)	Dominant season
PM054	2-Isopropylmalic acid	1	2-Isopropylmalic acid	0	0/3	$9.48 \times 10^5 \pm 1.64 \times 10^6$	1/3	0	0/3	S2
PM055	2-Isopropyloxypropane	1	2-Isopropyloxypropane	$1.94 \times 10^4 \pm 3.37 \times 10^4$	1/3	0	0/3	0	0/3	S1
PM056	2-Ketoisocaproic acid	1	2-Ketoisocaproic acid oxime	0	0/3	0	0/3	$1.08 \times 10^5 \pm 1.86 \times 10^5$	1/3	S3
PM057	2-linoleoylglycerol	1	2-linoleoylglycerol	$3.03 \times 10^6 \pm 2.62 \times 10^6$	2/3	$1.12 \times 10^7 \pm 4.14 \times 10^5$	3/3	0	0/3	S2
PM058	2-Methyl-butyrate	1	2-Methyl-butyrate	0	0/3	$2.91 \times 10^6 \pm 5.04 \times 10^6$	1/3	0	0/3	S2
PM059	2-Monolinolenoylglycerol	1	2-Monolinolenoylglycerol	$3.4 \times 10^7 \pm 2.73 \times 10^7$	3/3	$2.1 \times 10^7 \pm 6.78 \times 10^5$	3/3	$1.3 \times 10^7 \pm 1.68 \times 10^6$	3/3	S1
PM060	2-Monostearin	1	2-Monostearin	0	0/3	$4.25 \times 10^6 \pm 8.04 \times 10^5$	3/3	0	0/3	S2
PM061	2-Palmitoylglycerol	1	2-Palmitoylglycerol	0	0/3	$9.09 \times 10^6 \pm 6.37 \times 10^5$	3/3	0	0/3	S2
PM062	2-Pentenoic acid	1	2-Pentenoic acid	$1.53 \times 10^7 \pm 2.64 \times 10^7$	1/3	0	0/3	0	0/3	S1
PM063	2-Pentenoic acid, (E)-	1	2-Pentenoic acid, (E)-	0	0/3	0	0/3	$5.28 \times 10^5 \pm 4.87 \times 10^4$	3/3	S3
PM064	2-Propenoic acid	1	2-Propenoic acid	$1.99 \times 10^7 \pm 1.82 \times 10^7$	2/3	$2.39 \times 10^5 \pm 2.12 \times 10^5$	2/3	$1.04 \times 10^7 \pm 1.88 \times 10^6$	3/3	S1
PM065	3,4,5-Trihydroxypentanoic acid	1	3,4,5-Trihydroxypentanoic acid	$2.34 \times 10^6 \pm 4.05 \times 10^6$	1/3	$1.6 \times 10^6 \pm 2.01 \times 10^6$	2/3	0	0/3	S1
PM066	3,4-Dihydroxy-5-methyl-dihydrofuran-2-one, (D)-	1	3,4-Dihydroxy-5-methyl-dihydrofuran-2-one, (D)-	0	0/3	$9.66 \times 10^5 \pm 1.67 \times 10^6$	1/3	0	0/3	S2
PM067	3,4-Methylenedioxymandelic acid	1	3,4-Methylenedioxymandelic acid	0	0/3	0	0/3	$2.34 \times 10^6 \pm 1.56 \times 10^5$	3/3	S3
PM068	3,6,9,12-Tetraoxatridecanoic acid	1	3,6,9,12-Tetraoxatridecanoic acid	$2.21 \times 10^8 \pm 6.4 \times 10^6$	3/3	0	0/3	$8.41 \times 10^7 \pm 1.46 \times 10^8$	1/3	S1
PM069	3-(Benzyloxy)-1,2,4-butanetriol	1	3-(Benzyloxy)-1,2,4-butanetriol	0	0/3	$3.3 \times 10^7 \pm 2.12 \times 10^6$	3/3	$1.37 \times 10^7 \pm 2.37 \times 10^7$	1/3	S2
PM070	3-alpha-Mannobiose	1	3-.alpha.-Mannobiose, methyloxime (isomer 1)	0	0/3	0	0/3	$6.34 \times 10^5 \pm 1.1 \times 10^6$	1/3	S3
PM071	3-alpha-Mannobiose, (isomer 1)	1	3-.alpha.-Mannobiose, (isomer 1)	$4.52 \times 10^7 \pm 5.16 \times 10^7$	3/3	$7.83 \times 10^5 \pm 1.36 \times 10^6$	1/3	$4.22 \times 10^7 \pm 3.59 \times 10^7$	3/3	S1
PM072	3-alpha-Mannobiose, (isomer 2)	1	3-.alpha.-Mannobiose, (isomer 2)	$1.61 \times 10^8 \pm 5.71 \times 10^7$	3/3	$2.2 \times 10^7 \pm 2.8 \times 10^7$	3/3	$1.96 \times 10^6 \pm 9.11 \times 10^5$	3/3	S1
PM073	3-Aminoisobutyric acid	1	3-Aminoisobutyric acid	0	0/3	$2.59 \times 10^6 \pm 2.71 \times 10^6$	2/3	$9.87 \times 10^6 \pm 9.02 \times 10^6$	2/3	S3

Parent ID	Putative parent metabolite	Features (n)	Contributing retained feature form(s)	S1 mean ± SD (peak area)	S1 detected (n/3)	S2 mean ± SD (peak area)	S2 detected (n/3)	S3 mean ± SD (peak area)	S3 detected (n/3)	Dominant season
PM074	3-Butenoic acid	1	3-Butenoic acid	0	0/3	$2.88 \times 10^5 \pm 4.98 \times 10^5$	1/3	0	0/3	S2
PM075	3-Dimethyloxytridecane	1	3-Dimethyloxytridecane	$9.79 \times 10^4 \pm 1.7 \times 10^5$	1/3	0	0/3	0	0/3	S1
PM076	3-Hydroxy-3-(4'-hydroxy-3'-methoxyphenyl)propionic acid	1	3-Hydroxy-3-(4'-hydroxy-3'-methoxyphenyl)propionic acid	0	0/3	0	0/3	$3.64 \times 10^7 \pm 6.31 \times 10^7$	1/3	S3
PM077	3-Hydroxy-4-methoxybenzyl alcohol	1	3-Hydroxy-4-methoxybenzyl alcohol	$2.91 \times 10^5 \pm 2.77 \times 10^5$	2/3	0	0/3	0	0/3	S1
PM078	3-Hydroxydodecanedioic acid	1	3-Hydroxydodecanedioic acid	$8.42 \times 10^7 \pm 5.17 \times 10^6$	3/3	$2.73 \times 10^7 \pm 9.94 \times 10^6$	3/3	$4.67 \times 10^7 \pm 4.43 \times 10^6$	3/3	S1
PM079	3-Ketovaleric acid	1	3-Ketovaleric acid	$2.86 \times 10^6 \pm 4.96 \times 10^6$	1/3	0	0/3	0	0/3	S1
PM080	3-Methyl-2-ketobutyric acid	1	3-Methyl-2-ketobutyric acid	$3.04 \times 10^6 \pm 5.27 \times 10^6$	1/3	$6.82 \times 10^5 \pm 1.18 \times 10^6$	1/3	0	0/3	S1
PM081	3-Methylene-1,4-butane	1	3-Methylene-1,4-butane	0	0/3	$7.91 \times 10^5 \pm 1.37 \times 10^6$	1/3	0	0/3	S2
PM082	3-O-alpha-d-mannopyranosyl-4-O-beta-d-glucopyranosyl-d-glucitol	1	3-O-.alpha.-d-mannopyranosyl-4-O-.beta.-d-glucopyranosyl-d-glucitol	$2.87 \times 10^6 \pm 4.98 \times 10^6$	1/3	$1.43 \times 10^6 \pm 2.48 \times 10^6$	1/3	0	0/3	S1
PM083	3-Quinolinecarbonitrile	1	3-Quinolinecarbonitrile	0	0/3	0	0/3	$6.27 \times 10^5 \pm 1.09 \times 10^6$	1/3	S3
PM084	4,6-Dimethyl-1-oxa-4,6-diazacyclooctane-5-thione	1	4,6-Dimethyl-1-oxa-4,6-diazacyclooctane-5-thione	$3.27 \times 10^6 \pm 5.67 \times 10^6$	1/3	0	0/3	$3.02 \times 10^6 \pm 7.65 \times 10^5$	3/3	S1
PM085	4-(2-Acetylamino-1-(ethyl)phenol	1	4-(2-Acetylamino-1-(ethyl)phenol	$8.9 \times 10^6 \pm 1.54 \times 10^7$	1/3	0	0/3	$1.37 \times 10^7 \pm 1.2 \times 10^7$	2/3	S3
PM086	4-Aminobutanoic acid	1	4-Aminobutanoic acid	0	0/3	$2.06 \times 10^7 \pm 9.34 \times 10^5$	3/3	$3.38 \times 10^8 \pm 4.71 \times 10^7$	3/3	S3
PM087	4-Hydroxy-3-methoxyphenylglycol	1	4-Hydroxy-3-methoxyphenylglycol	$1.13 \times 10^7 \pm 1.02 \times 10^6$	3/3	0	0/3	$3.81 \times 10^7 \pm 5.66 \times 10^7$	2/3	S3
PM088	4-Hydroxybenzeneacetic acid	1	4-Hydroxybenzeneacetic acid	0	0/3	0	0/3	$2.11 \times 10^5 \pm 1.88 \times 10^5$	2/3	S3
PM089	4-Hydroxybenzoic acid	1	4-Hydroxybenzoic acid	0	0/3	$1.21 \times 10^7 \pm 4.93 \times 10^5$	3/3	0	0/3	S2
PM090	4-Hydroxybutanoic acid	1	4-Hydroxybutanoic acid	$3.29 \times 10^5 \pm 2.97 \times 10^5$	2/3	$3.23 \times 10^5 \pm 5.59 \times 10^5$	1/3	$8.65 \times 10^5 \pm 2.82 \times 10^5$	3/3	S3
PM091	4-Ketoglucose	1	4-Ketoglucose, bis(O-methyloxime)	$2.65 \times 10^6 \pm 4.59 \times 10^6$	1/3	0	0/3	$6.71 \times 10^6 \pm 3.57 \times 10^6$	3/3	S3
PM092	4-Methoxybutanoic acid	1	4-Methoxybutanoic acid	0	0/3	0	0/3	$1.16 \times 10^5 \pm 2.01 \times 10^5$	1/3	S3
PM093	4-Nitrophenyl-beta-D-galacturonide	1	4-Nitrophenyl-.beta.-D-galacturonide	0	0/3	0	0/3	$3.58 \times 10^6 \pm 3.11 \times 10^6$	2/3	S3

Parent ID	Putative parent metabolite	Features (n)	Contributing retained feature form(s)	S1 mean ± SD (peak area)	S1 detected (n/3)	S2 mean ± SD (peak area)	S2 detected (n/3)	S3 mean ± SD (peak area)	S3 detected (n/3)	Dominant season
PM094	5,5-Diethylheptadecane	1	5,5-Diethylheptadecane	$2.06 \times 10^5 \pm 3.56 \times 10^5$	1/3	$5.44 \times 10^4 \pm 9.43 \times 10^4$	1/3	0	0/3	S1
PM095	5,5-Diethylpentadecane	1	5,5-Diethylpentadecane	0	0/3	$7.47 \times 10^4 \pm 1.29 \times 10^5$	1/3	$1.25 \times 10^5 \pm 2.17 \times 10^5$	1/3	S3
PM096	5-Methyluridine	1	5-Methyluridine	$4.8 \times 10^6 \pm 7.44 \times 10^4$	3/3	$3.95 \times 10^7 \pm 3.09 \times 10^6$	3/3	$7.45 \times 10^6 \pm 1.29 \times 10^7$	1/3	S2
PM097	6,10,14-Trimethyl-2-pentadecanol	1	6,10,14-Trimethyl-2-pentadecanol	0	0/3	$1.04 \times 10^6 \pm 1.97 \times 10^5$	3/3	0	0/3	S2
PM098	6-(Dimethylamino)-4-oxohexanoic acid	1	6-(Dimethylamino)-4-oxohexanoic acid	0	0/3	$6.74 \times 10^5 \pm 1.17 \times 10^6$	1/3	0	0/3	S2
PM099	6-Cyanoquinoline	1	6-Cyanoquinoline	0	0/3	0	0/3	$3.13 \times 10^5 \pm 5.41 \times 10^5$	1/3	S3
PM100	6-Dimethyloxytetradecane	1	6-Dimethyloxytetradecane	0	0/3	0	0/3	$7.64 \times 10^4 \pm 1.32 \times 10^5$	1/3	S3
PM101	6-hydroxy-2-aminohexanoic acid	1	6-hydroxy-2-aminohexanoic acid	0	0/3	0	0/3	$3.23 \times 10^5 \pm 5.6 \times 10^5$	1/3	S3
PM102	6-Hydroxyflavone-beta-D-glucoside	1	6-Hydroxyflavone-.beta.-D-glucoside	0	0/3	0	0/3	$2.18 \times 10^6 \pm 3.77 \times 10^6$	1/3	S3
PM103	6-O-Methyl-2,4-methylene-beta-sedoheptitol	1	6-O-Methyl-2,4-methylene-.beta.-sedoheptitol	0	0/3	$4.35 \times 10^8 \pm 2.84 \times 10^7$	3/3	0	0/3	S2
PM104	7-oxy-7-methyloctanoic acid	1	7-oxy-7-methyloctanoic acid	0	0/3	$7.02 \times 10^5 \pm 1.22 \times 10^6$	1/3	0	0/3	S2
PM105	9,12-Octadecadienoic acid (Z,Z)-	1	9,12-Octadecadienoic acid (Z,Z)-	$4.67 \times 10^6 \pm 8.1 \times 10^6$	1/3	0	0/3	0	0/3	S1
PM106	9-Tetradecenoic acid, (E)-	1	9-Tetradecenoic acid, (E)-	0	0/3	$1.29 \times 10^6 \pm 2.23 \times 10^6$	1/3	$1.27 \times 10^6 \pm 2.2 \times 10^6$	1/3	S2
PM107	[1-(3,3-Dimethyloxiran-2-ylmethyl)-3,7-dimethylocta	1	[1-(3,3-Dimethyloxiran-2-ylmethyl)-3,7-dimethylocta	0	0/3	$3.59 \times 10^7 \pm 6.21 \times 10^7$	1/3	$2.84 \times 10^5 \pm 4.92 \times 10^5$	1/3	S2
PM108	[2-(2-Methoxyethoxy)ethoxy]acetic acid	1	[2-(2-Methoxyethoxy)ethoxy]acetic acid	0	0/3	0	0/3	$1.77 \times 10^8 \pm 1.54 \times 10^8$	2/3	S3
PM109	Acenaphthylene	1	Acenaphthylene	0	0/3	0	0/3	$1.11 \times 10^7 \pm 1.01 \times 10^7$	2/3	S3
PM110	Acetic acid, (methoxyimino)-	1	Acetic acid, (methoxyimino)-	0	0/3	$3.1 \times 10^5 \pm 5.37 \times 10^5$	1/3	0	0/3	S2
PM111	Acetin	1	Acetin	0	0/3	0	0/3	$3.51 \times 10^5 \pm 6.08 \times 10^5$	1/3	S3
PM112	Acetoacetic acid	1	Acetoacetic acid	$1.73 \times 10^5 \pm 3 \times 10^5$	1/3	0	0/3	0	0/3	S1
PM113	Acryl glycine	1	Acryl glycine	0	0/3	$3.72 \times 10^5 \pm 6.45 \times 10^5$	1/3	0	0/3	S2

Parent ID	Putative parent metabolite	Features (n)	Contributing retained feature form(s)	S1 mean ± SD (peak area)	S1 detected (n/3)	S2 mean ± SD (peak area)	S2 detected (n/3)	S3 mean ± SD (peak area)	S3 detected (n/3)	Dominant season
PM114	Acrylic acid, 2,3-bis[oxy]-	1	Acrylic acid, 2,3-bis[oxy]-	0	0/3	0	0/3	$3.2 \times 10^5 \pm 5.55 \times 10^5$	1/3	S3
PM115	Adonitol	1	Adonitol	$9.76 \times 10^7 \pm 1.69 \times 10^8$	1/3	0	0/3	0	0/3	S1
PM116	Alanine	1	Alanine	0	0/3	$1.05 \times 10^6 \pm 1.43 \times 10^6$	2/3	0	0/3	S2
PM117	Allonic acid, gamma-lactone	1	Allonic acid, .gamma.-lactone	$8.35 \times 10^6 \pm 7.25 \times 10^6$	2/3	$4.86 \times 10^6 \pm 4.22 \times 10^6$	2/3	$8.02 \times 10^6 \pm 1.8 \times 10^6$	3/3	S1
PM118	alpha-D-(+)-Talopyranose	1	.alpha.-D-(+)-Talopyranose	0	0/3	0	0/3	$1.87 \times 10^6 \pm 3.24 \times 10^6$	1/3	S3
PM119	alpha-D-Allopyranose	1	.alpha.-D-Allopyranose	$8.78 \times 10^6 \pm 1.52 \times 10^7$	1/3	0	0/3	0	0/3	S1
PM120	alpha-D-Glucopyranose	1	.alpha.-D-Glucopyranose	$8.54 \times 10^6 \pm 1.48 \times 10^7$	1/3	0	0/3	0	0/3	S1
PM121	alpha-d-Glucopyranoside, methyl, hexadecanoate	1	.alpha.-d-Glucopyranoside, methyl, hexadecanoate	$1.32 \times 10^6 \pm 2.96 \times 10^5$	3/3	$2.06 \times 10^6 \pm 2.71 \times 10^5$	3/3	$5.39 \times 10^6 \pm 9.34 \times 10^6$	1/3	S3
PM122	alpha-D-Glucopyranoside, methyl-6-dodecanoyl-	1	.alpha.-D-Glucopyranoside, methyl-6-dodecanoyl-	0	0/3	0	0/3	$8.26 \times 10^6 \pm 7.27 \times 10^6$	2/3	S3
PM123	alpha-D-Mannopyranose	1	.alpha.-D-Mannopyranose	0	0/3	0	0/3	$2.27 \times 10^6 \pm 3.93 \times 10^6$	1/3	S3
PM124	alpha-D-Mannopyranoside, methyl, cyclic butylboronate	1	.alpha.-D-Mannopyranoside, methyl, cyclic butylboronate	0	0/3	0	0/3	$8.08 \times 10^6 \pm 1.02 \times 10^7$	2/3	S3
PM125	Alpha-linolenic acid	1	.alpha.-Linolenic acid	$9.67 \times 10^7 \pm 6.29 \times 10^6$	3/3	$1.96 \times 10^9 \pm 8.95 \times 10^7$	3/3	$7.95 \times 10^8 \pm 7.9 \times 10^7$	3/3	S2
PM126	alpha-Tocopherol	1	.alpha.-Tocopherol	$3.54 \times 10^6 \pm 3.08 \times 10^6$	2/3	0	0/3	0	0/3	S1
PM127	Arabinitol	1	Arabinitol	$7.76 \times 10^7 \pm 6.75 \times 10^7$	2/3	$2.11 \times 10^6 \pm 1.27 \times 10^6$	3/3	$5.52 \times 10^7 \pm 4.78 \times 10^7$	2/3	S1
PM128	Arachidic acid	1	Arachidic acid	0	0/3	$4.16 \times 10^7 \pm 3.43 \times 10^6$	3/3	0	0/3	S2
PM129	Ascorbic acid	1	Ascorbic acid	$5.29 \times 10^6 \pm 6.41 \times 10^5$	3/3	0	0/3	$2.36 \times 10^6 \pm 4.31 \times 10^5$	3/3	S1
PM130	Azelaic acid	1	Azelaic acid	0	0/3	$2.68 \times 10^7 \pm 4.65 \times 10^7$	1/3	0	0/3	S2
PM131	beta-Alanine	1	.beta.-Alanine	0	0/3	0	0/3	$8.45 \times 10^6 \pm 5.89 \times 10^6$	3/3	S3
PM132	beta-D-(+)-Talopyranose	1	.beta.-D-(+)-Talopyranose	0	0/3	0	0/3	$2.57 \times 10^7 \pm 4.45 \times 10^7$	1/3	S3
PM133	beta-D-(+)-Xylopyranose	1	.beta.-D-(+)-Xylopyranose	$1.1 \times 10^6 \pm 1.91 \times 10^6$	1/3	0	0/3	$1.18 \times 10^6 \pm 2.04 \times 10^6$	1/3	S3

Parent ID	Putative parent metabolite	Features (n)	Contributing retained feature form(s)	S1 mean ± SD (peak area)	S1 detected (n/3)	S2 mean ± SD (peak area)	S2 detected (n/3)	S3 mean ± SD (peak area)	S3 detected (n/3)	Dominant season
PM134	beta-D-Lactose	1	.beta.-D-Lactose	0	0/3	0	0/3	$7.57 \times 10^5 \pm 1.31 \times 10^6$	1/3	S3
PM135	beta-D-Xylopyranose	1	.beta.-D-Xylopyranose	$4.01 \times 10^8 \pm 2.04 \times 10^8$	3/3	$2.61 \times 10^8 \pm 1.1 \times 10^7$	3/3	$2.1 \times 10^8 \pm 1.82 \times 10^8$	2/3	S1
PM136	beta-Gentiobiose	1	.beta.-Gentiobiose, methyloxime	$1.24 \times 10^7 \pm 7.9 \times 10^6$	3/3	0	0/3	0	0/3	S1
PM137	beta-Lyxopyranose	1	.beta.-Lyxopyranose	$2.67 \times 10^8 \pm 2.32 \times 10^8$	2/3	0	0/3	$1.34 \times 10^8 \pm 2.33 \times 10^8$	1/3	S1
PM138	Boric acid	1	Boric acid	$3.06 \times 10^5 \pm 3.55 \times 10^5$	2/3	0	0/3	0	0/3	S1
PM139	Butanal, 2,3,4-tris[oxy]-3-[[oxy]methyl]-, (S)-	1	Butanal, 2,3,4-tris[oxy]-3-[[oxy]methyl]-, O-methyloxime, (S)-	$3.06 \times 10^7 \pm 7.95 \times 10^6$	3/3	0	0/3	0	0/3	S1
PM140	Butanal, 3-(1-ethoxyethoxy)-2-methyl-	1	Butanal, 3-(1-ethoxyethoxy)-2-methyl-	$4.67 \times 10^5 \pm 8.08 \times 10^5$	1/3	0	0/3	0	0/3	S1
PM141	Butanedioic acid	1	Butanedioic acid	$6.31 \times 10^7 \pm 1.12 \times 10^7$	3/3	$4.34 \times 10^7 \pm 9.44 \times 10^6$	3/3	$5.41 \times 10^7 \pm 2 \times 10^7$	3/3	S1
PM142	Butanoic acid, 3,4-bis[oxy]-	1	Butanoic acid, 3,4-bis[oxy]-	0	0/3	$1.48 \times 10^6 \pm 1.04 \times 10^6$	3/3	0	0/3	S2
PM143	Caffeic acid	1	Caffeic acid	0	0/3	0	0/3	$1.12 \times 10^7 \pm 4.28 \times 10^5$	3/3	S3
PM144	Campesterol	1	Campesterol	$1.05 \times 10^6 \pm 1.82 \times 10^6$	1/3	$6.7 \times 10^7 \pm 3.98 \times 10^6$	3/3	$1.5 \times 10^7 \pm 2.33 \times 10^6$	3/3	S2
PM145	Canavanine	1	Canavanine	$2.39 \times 10^7 \pm 3.63 \times 10^6$	3/3	0	0/3	0	0/3	S1
PM146	Cholest-7-en-3-ol, (3.beta.,5.alpha.)-	1	Cholest-7-en-3-ol, (3.beta.,5.alpha.)-	0	0/3	$6.05 \times 10^7 \pm 6.28 \times 10^6$	3/3	0	0/3	S2
PM147	Cholesterol	1	Cholesterol	0	0/3	$1.77 \times 10^7 \pm 1.68 \times 10^6$	3/3	0	0/3	S2
PM148	Citric acid	1	Citric acid	0	0/3	$1.57 \times 10^8 \pm 1.61 \times 10^7$	3/3	$2.08 \times 10^8 \pm 4.86 \times 10^7$	3/3	S3
PM149	Citronellol	1	Citronellol	0	0/3	$1.46 \times 10^9 \pm 6.44 \times 10^7$	3/3	$8.02 \times 10^8 \pm 6.79 \times 10^7$	3/3	S2
PM150	cyclobutene, 3-methyl-1,2-bis[oxy]-	1	cyclobutene, 3-methyl-1,2-bis[oxy]-	$1.47 \times 10^6 \pm 2.55 \times 10^6$	1/3	0	0/3	0	0/3	S1
PM151	D-(+)-Arabitol	1	D-(+)-Arabitol	$1.93 \times 10^8 \pm 1.68 \times 10^8$	2/3	0	0/3	0	0/3	S1
PM152	D-(+)-Cellobiose	1	D-(+)-Cellobiose, methyloxime (isomer 2)	$3.12 \times 10^7 \pm 3.12 \times 10^6$	3/3	$1.61 \times 10^7 \pm 4.82 \times 10^6$	3/3	$4.12 \times 10^7 \pm 2.72 \times 10^7$	3/3	S3
PM153	D-(+)-Cellobiose, (isomer 1)	1	D-(+)-Cellobiose, (isomer 1)	$4.87 \times 10^6 \pm 8.43 \times 10^6$	1/3	$5.17 \times 10^5 \pm 8.96 \times 10^5$	1/3	$1.35 \times 10^6 \pm 2.34 \times 10^6$	1/3	S1

Parent ID	Putative parent metabolite	Features (n)	Contributing retained feature form(s)	S1 mean ± SD (peak area)	S1 detected (n/3)	S2 mean ± SD (peak area)	S2 detected (n/3)	S3 mean ± SD (peak area)	S3 detected (n/3)	Dominant season
PM154	D-(+)-Galactopyranose, (isomer 2)	1	D-(+)-Galactopyranose, (isomer 2)	$8.59 \times 10^6 \pm 1.49 \times 10^7$	1/3	0	0/3	0	0/3	S1
PM155	D-(+)-Galactose	3	D-(+)-Galactose oxime (isomer 1); D-(+)-Galactose oxime (isomer 2); D-(+)-Galactose, ethyloxime	$4.11 \times 10^7 \pm 1.35 \times 10^7$	3/3	$3.47 \times 10^7 \pm 1.47 \times 10^7$	3/3	$1.08 \times 10^7 \pm 2.04 \times 10^6$	3/3	S1
PM156	D-(+)-Galacturonic acid	1	D-(+)-Galacturonic acid	$3.88 \times 10^7 \pm 1.07 \times 10^7$	3/3	0	0/3	$1.91 \times 10^7 \pm 3.3 \times 10^7$	1/3	S1
PM157	D-(+)-Glucose	1	D-(+)-Glucose oxime	0	0/3	$3.15 \times 10^7 \pm 4.04 \times 10^6$	3/3	0	0/3	S2
PM158	D-(+)-Glucuronic acid gamma-lactone	1	D-(+)-Glucuronic acid .gamma.-lactone, methyloxime (anti)	$1.68 \times 10^6 \pm 2.91 \times 10^6$	1/3	0	0/3	0	0/3	S1
PM159	D-(+)-Ribono-1,4-lactone	1	D-(+)-Ribono-1,4-lactone	$6.76 \times 10^7 \pm 1.07 \times 10^7$	3/3	$1.94 \times 10^6 \pm 3.37 \times 10^6$	1/3	$1.23 \times 10^7 \pm 1.65 \times 10^7$	2/3	S1
PM160	D-(+)-Ribono-1,4-lactone (R,S,R)-	1	D-(+)-Ribono-1,4-lactone (R,S,R)-	0	0/3	$5.49 \times 10^5 \pm 9.51 \times 10^5$	1/3	$4.72 \times 10^5 \pm 4.43 \times 10^5$	2/3	S2
PM161	D-(+)-Talofuranose	1	D-(+)-Talofuranose	$8.72 \times 10^5 \pm 1.51 \times 10^6$	1/3	0	0/3	$7 \times 10^7 \pm 1.21 \times 10^8$	1/3	S3
PM162	D-(+)-Talose	1	D-(+)-Talose, methyloxime (syn)	0	0/3	0	0/3	$1.99 \times 10^8 \pm 3.46 \times 10^8$	1/3	S3
PM163	D-(+)-Trehalose	1	D-(+)-Trehalose	$4.23 \times 10^7 \pm 4.26 \times 10^6$	3/3	0	0/3	$4.03 \times 10^7 \pm 4.58 \times 10^6$	3/3	S1
PM164	D-(+)-Turanose	2	D-(+)-Turanose; D-(+)-Turanose, methyloxime	$1.09 \times 10^8 \pm 8.25 \times 10^7$	3/3	$1.03 \times 10^8 \pm 3.4 \times 10^7$	3/3	$1.26 \times 10^7 \pm 1.29 \times 10^7$	3/3	S1
PM165	D-(+)-Xylose	1	D-(+)-Xylose, methyloxime (anti)	$3.68 \times 10^7 \pm 6.38 \times 10^7$	1/3	0	0/3	$8.55 \times 10^5 \pm 7.41 \times 10^5$	2/3	S1
PM166	D-(-)- Erythrofuranose, (isomer 1)	1	D-(-)- Erythrofuranose, (isomer 1)	$1.7 \times 10^5 \pm 2.95 \times 10^5$	1/3	0	0/3	0	0/3	S1
PM167	D-(-)-Erythrose	1	D-(-)-Erythrose, ethyloxime (isomer 1)	0	0/3	0	0/3	$4.39 \times 10^6 \pm 7.61 \times 10^6$	1/3	S3
PM168	D-(-)-Fructofuranose, (isomer 2)	1	D-(-)-Fructofuranose, (isomer 2)	$8.28 \times 10^6 \pm 1.43 \times 10^7$	1/3	$3.73 \times 10^8 \pm 3.24 \times 10^8$	2/3	$2.02 \times 10^8 \pm 3.43 \times 10^8$	2/3	S2
PM169	D-(-)-Fructopyranose, (isomer 2)	1	D-(-)-Fructopyranose, (isomer 2)	0	0/3	0	0/3	$2.72 \times 10^7 \pm 2.37 \times 10^7$	2/3	S3
PM170	D-(-)-Rhamnose	2	D-(-)-Rhamnose, methyloxime (anti); D-(-)-Rhamnose, methyloxime (syn)	0	0/3	0	0/3	$3.79 \times 10^7 \pm 4.22 \times 10^6$	3/3	S3
PM171	D-(-)-Ribofuranose, (isomer 1)	1	D-(-)-Ribofuranose, (isomer 1)	$1.11 \times 10^7 \pm 9.67 \times 10^6$	2/3	$9.35 \times 10^6 \pm 2.98 \times 10^6$	3/3	$1.36 \times 10^7 \pm 3.98 \times 10^6$	3/3	S3
PM172	D-(-)-Ribofuranose, (isomer 2)	1	D-(-)-Ribofuranose, (isomer 2)	$7.45 \times 10^7 \pm 1.08 \times 10^8$	3/3	0	0/3	0	0/3	S1
PM173	D-(-)-Tagatofuranose, (isomer 2)	1	D-(-)-Tagatofuranose, (isomer 2)	$3.1 \times 10^8 \pm 5.14 \times 10^8$	2/3	$3.3 \times 10^8 \pm 2.99 \times 10^8$	2/3	$6.09 \times 10^7 \pm 1.05 \times 10^8$	1/3	S2

Parent ID	Putative parent metabolite	Features (n)	Contributing retained feature form(s)	S1 mean ± SD (peak area)	S1 detected (n/3)	S2 mean ± SD (peak area)	S2 detected (n/3)	S3 mean ± SD (peak area)	S3 detected (n/3)	Dominant season
PM174	D-(-)-Tagatose, (isomer 1)	1	D-(-)-Tagatose, (isomer 1)	0	0/3	0	0/3	$5.05 \times 10^4 \pm 8.75 \times 10^4$	1/3	S3
PM175	D-2-Deoxyribose	1	D-2-Deoxyribose	$1.33 \times 10^6 \pm 2.3 \times 10^6$	1/3	$7.91 \times 10^5 \pm 1.37 \times 10^6$	1/3	$1.71 \times 10^6 \pm 1.52 \times 10^6$	2/3	S3
PM176	D-Allose	2	D-Allose, oxime; D-Allose, ethyloxime	$8.3 \times 10^7 \pm 1.13 \times 10^7$	3/3	$3.52 \times 10^7 \pm 4.98 \times 10^6$	3/3	$6.73 \times 10^7 \pm 2.43 \times 10^7$	3/3	S1
PM177	D-Altrose	2	D-Altrose, ethyloxime; D-Altrose, pentafluorobenzyloxime	$3.61 \times 10^5 \pm 6.25 \times 10^5$	1/3	0	0/3	$2.03 \times 10^8 \pm 3.33 \times 10^8$	2/3	S3
PM178	D-Arabinopyranose	1	D-Arabinopyranose	$1.23 \times 10^6 \pm 2.13 \times 10^6$	1/3	0	0/3	0	0/3	S1
PM179	D-Arabinose	2	D-Arabinose, ethyloxime; D-Arabinose, methyloxime (syn)	$9.52 \times 10^7 \pm 7.9 \times 10^6$	3/3	$3.12 \times 10^7 \pm 1.92 \times 10^6$	3/3	$9.89 \times 10^7 \pm 1.73 \times 10^7$	3/3	S3
PM180	D-Erythro-Pentitol, 2-deoxy-	1	D-Erythro-Pentitol, 2-deoxy-	$3 \times 10^7 \pm 5.68 \times 10^6$	3/3	$1.22 \times 10^6 \pm 2.11 \times 10^6$	1/3	$4.16 \times 10^6 \pm 3.03 \times 10^6$	3/3	S1
PM181	D-Fructose	2	D-Fructose, O-methyloxime; D-Fructose	$1.56 \times 10^9 \pm 1.18 \times 10^8$	3/3	$7.3 \times 10^8 \pm 3.34 \times 10^8$	3/3	$1.64 \times 10^9 \pm 4.17 \times 10^8$	3/3	S3
PM182	D-Fructose, 3-O-[alpha-D-glucopyranosyl]	1	D-Fructose, 3-O-[.alpha.-D-glucopyranosyl]	$7.27 \times 10^6 \pm 1.26 \times 10^7$	1/3	0	0/3	$5.49 \times 10^5 \pm 9.5 \times 10^5$	1/3	S1
PM183	D-Fucitol	1	D-Fucitol	$9.97 \times 10^7 \pm 8.7 \times 10^6$	3/3	$9.4 \times 10^7 \pm 8.15 \times 10^7$	2/3	0	0/3	S1
PM184	D-Galactose	1	D-Galactose	$3.3 \times 10^7 \pm 2.86 \times 10^7$	2/3	0	0/3	0	0/3	S1
PM185	D-Glucitol	1	D-Glucitol	$1.97 \times 10^8 \pm 4.19 \times 10^6$	3/3	$7.62 \times 10^8 \pm 2.04 \times 10^7$	3/3	$1.83 \times 10^8 \pm 2.12 \times 10^7$	3/3	S2
PM186	D-Gluconic acid	1	D-Gluconic acid	$2.49 \times 10^5 \pm 4.32 \times 10^5$	1/3	$1.65 \times 10^7 \pm 1.02 \times 10^6$	3/3	$5.37 \times 10^6 \pm 7.1 \times 10^6$	2/3	S2
PM187	D-Glucose	2	D-Glucose, O-methyloxime; D-Glucose	0	0/3	$5.63 \times 10^8 \pm 2.88 \times 10^6$	3/3	$1.34 \times 10^7 \pm 1.18 \times 10^7$	2/3	S2
PM188	d-Glucose, o-methyloxyme, (1Z)-	1	d-Glucose, o-methyloxyme, (1Z)-	0	0/3	0	0/3	$1.27 \times 10^6 \pm 2.19 \times 10^6$	1/3	S3
PM189	D-Glycero-D-gulo-Heptose	1	D-Glycero-D-gulo-Heptose, O-methyloxime	$2.35 \times 10^8 \pm 4.63 \times 10^6$	3/3	$2.69 \times 10^8 \pm 1.28 \times 10^7$	3/3	$2.09 \times 10^8 \pm 2 \times 10^7$	3/3	S2
PM190	D-Lactose	2	D-Lactose; D-Lactose, methyloxime	$1.72 \times 10^6 \pm 2.98 \times 10^6$	1/3	$2.25 \times 10^5 \pm 3.9 \times 10^5$	1/3	$1.69 \times 10^7 \pm 2.92 \times 10^7$	1/3	S3
PM191	D-limonene	1	D-Limonene	0	0/3	0	0/3	$7.66 \times 10^4 \pm 1.33 \times 10^5$	1/3	S3
PM192	D-Mannitol	1	D-Mannitol	$9.32 \times 10^6 \pm 1.26 \times 10^6$	3/3	0	0/3	$8.36 \times 10^6 \pm 1.45 \times 10^7$	1/3	S1
PM193	D-Mannopyranose	1	D-Mannopyranose	$3.87 \times 10^5 \pm 6.71 \times 10^5$	1/3	0	0/3	$2.44 \times 10^6 \pm 4.23 \times 10^6$	1/3	S3

Parent ID	Putative parent metabolite	Features (n)	Contributing retained feature form(s)	S1 mean ± SD (peak area)	S1 detected (n/3)	S2 mean ± SD (peak area)	S2 detected (n/3)	S3 mean ± SD (peak area)	S3 detected (n/3)	Dominant season
PM194	d-Mannose, o-methyloxyme, (1Z)-	1	d-Mannose, o-methyloxyme, (1Z)-	0	0/3	$5.64 \times 10^6 \pm 5.54 \times 10^6$	2/3	0	0/3	S2
PM195	D-Psicofuranose	1	D-Psicofuranose	$8.6 \times 10^6 \pm 3.91 \times 10^5$	3/3	$2.36 \times 10^7 \pm 1.12 \times 10^7$	3/3	$2.45 \times 10^7 \pm 3.89 \times 10^7$	3/3	S3
PM196	D-Psicose	1	D-Psicose, methyloxime (syn)	$2.36 \times 10^7 \pm 3.06 \times 10^5$	3/3	0	0/3	0	0/3	S1
PM197	D-Ribopyranose	1	D-Ribopyranose	0	0/3	$6.6 \times 10^8 \pm 2.63 \times 10^7$	3/3	$1.07 \times 10^9 \pm 7.94 \times 10^7$	3/3	S3
PM198	D-Trehalose	1	D-Trehalose	$1.79 \times 10^8 \pm 1.44 \times 10^7$	3/3	$5.03 \times 10^7 \pm 2.35 \times 10^7$	3/3	$1.4 \times 10^8 \pm 3.28 \times 10^7$	3/3	S1
PM199	D-Xylopyranose	1	D-Xylopyranose	$5.46 \times 10^6 \pm 7.9 \times 10^5$	3/3	$8.04 \times 10^6 \pm 6.98 \times 10^6$	2/3	0	0/3	S2
PM200	Deoxyfructosazine	1	Deoxyfructosazine	0	0/3	$1.44 \times 10^7 \pm 1.2 \times 10^6$	3/3	0	0/3	S2
PM201	Deoxyglucose	1	Deoxyglucose	$5.6 \times 10^5 \pm 9.69 \times 10^5$	1/3	0	0/3	$2.25 \times 10^6 \pm 3.27 \times 10^5$	3/3	S3
PM202	Diethylmalonic acid	1	Diethylmalonic acid	0	0/3	$5.56 \times 10^5 \pm 9.62 \times 10^5$	1/3	0	0/3	S2
PM203	DL-Arabinose	1	DL-Arabinose, methyloxime	$7.18 \times 10^7 \pm 6.26 \times 10^7$	2/3	0	0/3	0	0/3	S1
PM204	DL-Phenylalanine	1	DL-Phenylalanine	0	0/3	$6.52 \times 10^7 \pm 3.37 \times 10^7$	3/3	$4.11 \times 10^7 \pm 3.67 \times 10^7$	2/3	S2
PM205	Dodecane, 2,6,11-trimethyl-	1	Dodecane, 2,6,11-trimethyl-	0	0/3	0	0/3	$2.1 \times 10^6 \pm 3.64 \times 10^6$	1/3	S3
PM206	Dodecanoic acid	1	Dodecanoic acid	0	0/3	$7.52 \times 10^6 \pm 2.5 \times 10^6$	3/3	0	0/3	S2
PM207	Dodecyl nonyl ether	1	Dodecyl nonyl ether	$2.78 \times 10^5 \pm 4.82 \times 10^5$	1/3	0	0/3	0	0/3	S1
PM208	Eicosane	1	Eicosane	$7.15 \times 10^6 \pm 6.73 \times 10^5$	3/3	$3.69 \times 10^6 \pm 4.79 \times 10^6$	2/3	$1.58 \times 10^6 \pm 1.52 \times 10^5$	3/3	S1
PM209	Ephedrine	1	Ephedrine	$5.89 \times 10^7 \pm 5.11 \times 10^7$	2/3	0	0/3	0	0/3	S1
PM210	Erythritol	1	Erythritol	0	0/3	$1.54 \times 10^8 \pm 8.85 \times 10^7$	3/3	$3.28 \times 10^7 \pm 1.52 \times 10^7$	3/3	S2
PM211	Erythro-Pentonic acid	1	Erythro-Pentonic acid	0	0/3	0	0/3	$3.84 \times 10^6 \pm 3.42 \times 10^6$	2/3	S3
PM212	Erythrono-1,4-lactone	1	Erythrono-1,4-lactone	0	0/3	$4.18 \times 10^5 \pm 7.25 \times 10^5$	1/3	$1.58 \times 10^6 \pm 3.9 \times 10^5$	3/3	S3
PM213	Erythrono-1,4-lactone, (Z)-	1	Erythrono-1,4-lactone, (Z)-	$4.01 \times 10^6 \pm 4.45 \times 10^6$	2/3	0	0/3	$5.35 \times 10^5 \pm 9.27 \times 10^5$	1/3	S1

Parent ID	Putative parent metabolite	Features (n)	Contributing retained feature form(s)	S1 mean ± SD (peak area)	S1 detected (n/3)	S2 mean ± SD (peak area)	S2 detected (n/3)	S3 mean ± SD (peak area)	S3 detected (n/3)	Dominant season
PM214	Ethanamine, N-ethyl-2-methoxy-N-(2-methoxyethyl)-	1	Ethanamine, N-ethyl-2-methoxy-N-(2-methoxyethyl)-	$3.57 \times 10^5 \pm 6.19 \times 10^5$	1/3	0	0/3	0	0/3	S1
PM215	Ethanol, 2-(9,12-octadecadienyloxy)-, (Z,Z)-	1	Ethanol, 2-(9,12-octadecadienyloxy)-, (Z,Z)-	$1.17 \times 10^6 \pm 2.02 \times 10^6$	1/3	0	0/3	0	0/3	S1
PM216	Ethanolamine	1	Ethanolamine	$2.92 \times 10^7 \pm 5.06 \times 10^7$	1/3	0	0/3	$2.4 \times 10^8 \pm 2.65 \times 10^7$	3/3	S3
PM217	Ethyl alpha-D-glucopyranoside	1	Ethyl .alpha.-D-glucopyranoside	$2.65 \times 10^6 \pm 1.22 \times 10^6$	3/3	$1.39 \times 10^8 \pm 1.2 \times 10^8$	2/3	$4.02 \times 10^7 \pm 6.39 \times 10^6$	3/3	S2
PM218	Ethyl cis-2-cyclopropane-1-carboxylate	1	Ethyl cis-2-cyclopropane-1-carboxylate	0	0/3	$2.63 \times 10^5 \pm 4.56 \times 10^5$	1/3	0	0/3	S2
PM219	Ethyl glycolate	1	Ethyl glycolate	$6.07 \times 10^5 \pm 2.03 \times 10^5$	3/3	0	0/3	$7.25 \times 10^5 \pm 6.29 \times 10^5$	2/3	S3
PM220	Ethyl(2-hydroxyethyl)carbamic acid	1	Ethyl(2-hydroxyethyl)carbamic acid	0	0/3	0	0/3	$1.68 \times 10^6 \pm 2.92 \times 10^6$	1/3	S3
PM221	Ethylene glycol	1	Ethylene glycol	$1.27 \times 10^5 \pm 2.19 \times 10^5$	1/3	0	0/3	$3.1 \times 10^6 \pm 7.05 \times 10^5$	3/3	S3
PM222	Ethylphosphonic acid	1	Ethylphosphonic acid	$1.13 \times 10^8 \pm 8.45 \times 10^6$	3/3	0	0/3	$7.11 \times 10^6 \pm 1.95 \times 10^6$	3/3	S1
PM223	Farnesol	1	Farnesol	0	0/3	$4.53 \times 10^7 \pm 2.51 \times 10^6$	3/3	0	0/3	S2
PM224	Ferulic acid	1	Ferulic acid	0	0/3	$1.45 \times 10^7 \pm 1.27 \times 10^7$	2/3	0	0/3	S2
PM225	Galactaric acid	1	Galactaric acid	$2.45 \times 10^7 \pm 2.13 \times 10^7$	2/3	0	0/3	$1.77 \times 10^7 \pm 1.92 \times 10^6$	3/3	S1
PM226	Galactaric acid, (R,S,R,S)-	1	Galactaric acid, (R,S,R,S)-	0	0/3	0	0/3	$9.83 \times 10^6 \pm 8.55 \times 10^6$	2/3	S3
PM227	Galacturonic acid	1	Galacturonic acid	$9.08 \times 10^7 \pm 9.31 \times 10^7$	3/3	$9.93 \times 10^6 \pm 3.28 \times 10^5$	3/3	$3.31 \times 10^7 \pm 4.92 \times 10^6$	3/3	S1
PM228	Glucaric acid, gamma-lactone	1	Glucaric acid, .gamma.-lactone	$4.65 \times 10^6 \pm 1.81 \times 10^6$	3/3	0	0/3	0	0/3	S1
PM229	Glucopyranose	1	Glucopyranose	$2.79 \times 10^7 \pm 1.87 \times 10^6$	3/3	$8.26 \times 10^6 \pm 1.43 \times 10^7$	1/3	$2.58 \times 10^7 \pm 2.49 \times 10^7$	2/3	S1
PM230	Glucose	1	Glucose	$6.1 \times 10^5 \pm 1.06 \times 10^6$	1/3	0	0/3	0	0/3	S1
PM231	Glyceric acid	1	Glyceric acid	$4.68 \times 10^7 \pm 6.48 \times 10^6$	3/3	$3.24 \times 10^7 \pm 4.81 \times 10^6$	3/3	$3.18 \times 10^7 \pm 1.67 \times 10^7$	3/3	S1
PM232	Glycerol	1	Glycerol	$1.28 \times 10^9 \pm 7.29 \times 10^7$	3/3	$1.23 \times 10^9 \pm 2.35 \times 10^8$	3/3	$6.9 \times 10^8 \pm 1.89 \times 10^8$	3/3	S1
PM233	Glycerol monostearate	1	Glycerol monostearate	$7.56 \times 10^7 \pm 8.35 \times 10^5$	3/3	$1.16 \times 10^8 \pm 6.78 \times 10^6$	3/3	$7.01 \times 10^7 \pm 8.36 \times 10^6$	3/3	S2

Parent ID	Putative parent metabolite	Features (n)	Contributing retained feature form(s)	S1 mean ± SD (peak area)	S1 detected (n/3)	S2 mean ± SD (peak area)	S2 detected (n/3)	S3 mean ± SD (peak area)	S3 detected (n/3)	Dominant season
PM234	Glyceryl-glycoside	1	Glyceryl-glycoside	$5.76 \times 10^7 \pm 1.38 \times 10^6$	3/3	$1.28 \times 10^8 \pm 9.83 \times 10^6$	3/3	$1.42 \times 10^8 \pm 1.69 \times 10^7$	3/3	S3
PM235	Glycine	1	Glycine	$4.3 \times 10^5 \pm 7.44 \times 10^5$	1/3	0	0/3	$7.81 \times 10^6 \pm 1.35 \times 10^7$	1/3	S3
PM236	Glycolic acid	1	Glycolic acid	$3.4 \times 10^7 \pm 1.41 \times 10^7$	3/3	$6.47 \times 10^7 \pm 4.99 \times 10^6$	3/3	$6.71 \times 10^7 \pm 1.25 \times 10^7$	3/3	S3
PM237	Guaicol-beta-d-glucopyranoside	1	Guaicol-.beta.-d-glucopyranoside	0	0/3	$4.46 \times 10^6 \pm 3.87 \times 10^6$	2/3	0	0/3	S2
PM238	Gulonic acid, gamma-lactone	1	Gulonic acid, .gamma.-lactone	0	0/3	0	0/3	$1.96 \times 10^7 \pm 3.4 \times 10^7$	1/3	S3
PM239	Heneicosanoic acid	1	Heneicosanoic acid	0	0/3	$1.62 \times 10^7 \pm 1.62 \times 10^6$	3/3	0	0/3	S2
PM240	Hentriacontane	1	Hentriacontane	0	0/3	$4.39 \times 10^5 \pm 7.6 \times 10^5$	1/3	0	0/3	S2
PM241	Heptadecanoic acid	1	Heptadecanoic acid	$1.93 \times 10^6 \pm 1.87 \times 10^6$	2/3	$1.16 \times 10^7 \pm 1.29 \times 10^6$	3/3	0	0/3	S2
PM242	Hexacosanoic acid	1	Hexacosanoic acid	0	0/3	$1.15 \times 10^7 \pm 6.89 \times 10^5$	3/3	0	0/3	S2
PM243	Hexadecane, 2,6,10,14-tetramethyl-	1	Hexadecane, 2,6,10,14-tetramethyl-	0	0/3	0	0/3	$2.66 \times 10^6 \pm 4.61 \times 10^6$	1/3	S3
PM244	Hexadecyl isopropyl ether	1	Hexadecyl isopropyl ether	$1.93 \times 10^7 \pm 3.35 \times 10^7$	1/3	$1.66 \times 10^6 \pm 2.87 \times 10^6$	1/3	0	0/3	S1
PM245	Hexanedioic acid	1	Hexanedioic acid	$7.7 \times 10^6 \pm 1.33 \times 10^7$	1/3	$6.91 \times 10^5 \pm 1.61 \times 10^5$	3/3	$3.6 \times 10^6 \pm 4.88 \times 10^6$	3/3	S1
PM246	Hexanoic acid	1	Hexanoic acid	0	0/3	$9.43 \times 10^5 \pm 1.63 \times 10^6$	1/3	0	0/3	S2
PM247	Hydracrylic acid	1	Hydracrylic acid	$6.51 \times 10^6 \pm 1.46 \times 10^6$	3/3	$3.18 \times 10^7 \pm 4.02 \times 10^6$	3/3	$3.63 \times 10^7 \pm 4.16 \times 10^6$	3/3	S3
PM248	Inositol, (Z)-	1	Inositol, (Z)-	0	0/3	0	0/3	$3.17 \times 10^5 \pm 5.49 \times 10^5$	1/3	S3
PM249	Isopropyl octadecyl ether	1	Isopropyl octadecyl ether	0	0/3	$1.75 \times 10^6 \pm 3.03 \times 10^6$	1/3	0	0/3	S2
PM250	Isopropyl tetradecyl ether	1	Isopropyl tetradecyl ether	$8.47 \times 10^6 \pm 1.47 \times 10^7$	1/3	0	0/3	$9.04 \times 10^6 \pm 4.04 \times 10^6$	3/3	S3
PM251	L-(+)-Erythrulose	1	L-(+)-Erythrulose, ethyloxime	$1.73 \times 10^6 \pm 3 \times 10^6$	1/3	0	0/3	$3.79 \times 10^6 \pm 5.33 \times 10^6$	2/3	S3
PM252	L-(+)-Rhamnopyranose	1	L-(+)-Rhamnopyranose	$2.14 \times 10^7 \pm 1.93 \times 10^7$	2/3	0	0/3	0	0/3	S1
PM253	L-(+)-Tartaric acid	1	L-(+)-Tartaric acid	$1.18 \times 10^7 \pm 2.04 \times 10^7$	1/3	0	0/3	$4.34 \times 10^7 \pm 8.28 \times 10^6$	3/3	S3

Parent ID	Putative parent metabolite	Features (n)	Contributing retained feature form(s)	S1 mean ± SD (peak area)	S1 detected (n/3)	S2 mean ± SD (peak area)	S2 detected (n/3)	S3 mean ± SD (peak area)	S3 detected (n/3)	Dominant season
PM254	L-(+)-Threose	2	L-(+)-Threose; L-(+)-Threose, ethyloxime	$2.47 \times 10^5 \pm 2.39 \times 10^5$	2/3	0	0/3	$4.84 \times 10^6 \pm 8.38 \times 10^6$	1/3	S3
PM255	L-(-)-Arabitol	1	L-(-)-Arabitol	$4.19 \times 10^7 \pm 7.26 \times 10^7$	1/3	$8.77 \times 10^5 \pm 1.52 \times 10^6$	1/3	$8.3 \times 10^7 \pm 1.44 \times 10^8$	1/3	S3
PM256	L-(-)-Sorbose	1	L-(-)-Sorbose	$1.82 \times 10^6 \pm 3.16 \times 10^6$	1/3	0	0/3	0	0/3	S1
PM257	L-5-Oxoproline	1	L-5-Oxoproline	0	0/3	$1.15 \times 10^7 \pm 2.24 \times 10^6$	3/3	$1.28 \times 10^8 \pm 1.78 \times 10^7$	3/3	S3
PM258	L-Alanine	1	L-Alanine	$1.04 \times 10^8 \pm 1.27 \times 10^7$	3/3	$2.55 \times 10^7 \pm 3.24 \times 10^6$	3/3	$1.02 \times 10^8 \pm 3.83 \times 10^7$	3/3	S1
PM259	L-Alanine, N-formyl-	1	L-Alanine, N-formyl-	0	0/3	$2.38 \times 10^6 \pm 2.12 \times 10^6$	2/3	0	0/3	S2
PM260	L-Aspartic acid	1	L-Aspartic acid	0	0/3	$3.6 \times 10^7 \pm 5.6 \times 10^6$	3/3	$1.23 \times 10^7 \pm 2.52 \times 10^6$	3/3	S2
PM261	L-Citrulline	1	L-Citrulline	0	0/3	$1.5 \times 10^6 \pm 2.6 \times 10^6$	1/3	0	0/3	S2
PM262	L-Fucitol	1	L-Fucitol	0	0/3	0	0/3	$3.85 \times 10^7 \pm 6.67 \times 10^7$	1/3	S3
PM263	L-Homoserine	1	L-Homoserine	0	0/3	$1.98 \times 10^6 \pm 5.66 \times 10^5$	3/3	0	0/3	S2
PM264	L-Isoleucine	1	L-Isoleucine	0	0/3	$2.3 \times 10^5 \pm 3.99 \times 10^5$	1/3	$4.95 \times 10^8 \pm 8.1 \times 10^7$	3/3	S3
PM265	L-Leucine	1	L-Leucine	$1.65 \times 10^7 \pm 1.66 \times 10^6$	3/3	0	0/3	$4.17 \times 10^6 \pm 9.75 \times 10^5$	3/3	S1
PM266	L-Norvaline	1	L-Norvaline	0	0/3	$2.73 \times 10^6 \pm 2.48 \times 10^6$	2/3	$9.16 \times 10^7 \pm 1.59 \times 10^8$	1/3	S3
PM267	L-Phenylalanine	1	L-Phenylalanine	0	0/3	$2.96 \times 10^5 \pm 5.12 \times 10^5$	1/3	0	0/3	S2
PM268	L-Proline	1	L-Proline	$3.66 \times 10^7 \pm 2.11 \times 10^7$	3/3	$3.82 \times 10^7 \pm 1.17 \times 10^7$	3/3	$1.65 \times 10^8 \pm 1.29 \times 10^8$	3/3	S3
PM269	L-Rhamnose	1	L-Rhamnose	$1.13 \times 10^7 \pm 1.96 \times 10^7$	1/3	$3.73 \times 10^5 \pm 6.46 \times 10^5$	1/3	$4.19 \times 10^5 \pm 7.25 \times 10^5$	1/3	S1
PM270	L-Serine	1	L-Serine	0	0/3	$7.02 \times 10^6 \pm 2.09 \times 10^6$	3/3	$3.69 \times 10^6 \pm 9.27 \times 10^5$	3/3	S2
PM271	L-Threonic acid	1	L-Threonic acid	$4.56 \times 10^7 \pm 4 \times 10^7$	2/3	0	0/3	$8.16 \times 10^7 \pm 2.31 \times 10^7$	3/3	S3
PM272	L-Threonine	1	L-Threonine	0	0/3	$2.31 \times 10^7 \pm 2.2 \times 10^7$	3/3	$1.38 \times 10^7 \pm 1.1 \times 10^7$	3/3	S2
PM273	L-Tryptophan	1	L-Tryptophan	0	0/3	0	0/3	$5.24 \times 10^7 \pm 4.14 \times 10^6$	3/3	S3

Parent ID	Putative parent metabolite	Features (n)	Contributing retained feature form(s)	S1 mean ± SD (peak area)	S1 detected (n/3)	S2 mean ± SD (peak area)	S2 detected (n/3)	S3 mean ± SD (peak area)	S3 detected (n/3)	Dominant season
PM274	L-Valine	1	L-Valine	0	0/3	$1.46 \times 10^7 \pm 2.16 \times 10^6$	3/3	$1.5 \times 10^8 \pm 1.05 \times 10^8$	3/3	S3
PM275	Lactic Acid	1	Lactic Acid	$1.36 \times 10^7 \pm 3.29 \times 10^6$	3/3	$2.32 \times 10^7 \pm 1.14 \times 10^6$	3/3	$1.35 \times 10^7 \pm 3.16 \times 10^6$	3/3	S2
PM276	Lactulose	2	Lactulose; Lactulose, methyloxime	$2.1 \times 10^6 \pm 3.63 \times 10^6$	1/3	$7.51 \times 10^5 \pm 6.53 \times 10^5$	2/3	$2.2 \times 10^6 \pm 5.7 \times 10^4$	3/3	S3
PM277	Levoglucosan	1	Levoglucosan	$4.72 \times 10^7 \pm 6.1 \times 10^6$	3/3	0	0/3	$1.28 \times 10^7 \pm 1.12 \times 10^7$	2/3	S1
PM278	Lignoceric acid	1	Lignoceric acid	0	0/3	$7.67 \times 10^7 \pm 2.19 \times 10^6$	3/3	$8 \times 10^6 \pm 1.16 \times 10^6$	3/3	S2
PM279	Malic acid	1	Malic acid	$1.11 \times 10^8 \pm 6.49 \times 10^6$	3/3	$1.26 \times 10^8 \pm 2.33 \times 10^7$	3/3	$1.14 \times 10^8 \pm 3.14 \times 10^7$	3/3	S2
PM280	Malonic acid	1	Malonic acid	$1.16 \times 10^5 \pm 2.01 \times 10^5$	1/3	0	0/3	0	0/3	S1
PM281	Maltitol	1	Maltitol	$1.19 \times 10^7 \pm 2.06 \times 10^7$	1/3	0	0/3	$3.44 \times 10^5 \pm 5.96 \times 10^5$	1/3	S1
PM282	Maltose	2	Maltose; Maltose, methyloxime	$2.36 \times 10^7 \pm 9.71 \times 10^6$	3/3	$4.37 \times 10^8 \pm 1.24 \times 10^7$	3/3	$1.13 \times 10^8 \pm 2.39 \times 10^7$	3/3	S2
PM283	Mannonic acid, lactone -	1	Mannonic acid, lactone -	0	0/3	0	0/3	$7.41 \times 10^5 \pm 1.28 \times 10^6$	1/3	S3
PM284	Melibiose	1	Melibiose	0	0/3	0	0/3	$1.05 \times 10^6 \pm 1.82 \times 10^6$	1/3	S3
PM285	meso-Erythritol	1	meso-Erythritol	$7.76 \times 10^7 \pm 1.28 \times 10^7$	3/3	$5.69 \times 10^7 \pm 9.86 \times 10^7$	1/3	$1.73 \times 10^6 \pm 3 \times 10^6$	1/3	S1
PM286	Methcathinone	1	Methcathinone	0	0/3	0	0/3	$3.24 \times 10^4 \pm 5.62 \times 10^4$	1/3	S3
PM287	Methoxyacetic acid	1	Methoxyacetic acid	0	0/3	$3.24 \times 10^4 \pm 5.61 \times 10^4$	1/3	0	0/3	S2
PM288	Methyl 19-methyl-eicosanoate	1	Methyl 19-methyl-eicosanoate	0	0/3	$3.76 \times 10^8 \pm 3.26 \times 10^8$	2/3	0	0/3	S2
PM289	Methyl 2,6-dihydroxybenzoate	1	Methyl 2,6-dihydroxybenzoate	0	0/3	$1.41 \times 10^5 \pm 2.44 \times 10^5$	1/3	0	0/3	S2
PM290	Methyl galactoside	1	Methyl galactoside	$2.61 \times 10^7 \pm 2.35 \times 10^6$	3/3	$7.37 \times 10^6 \pm 1.28 \times 10^7$	1/3	$3.94 \times 10^7 \pm 3.95 \times 10^7$	3/3	S3
PM291	Methyl galactoside (1S,2S,3S,4R,5R)-	1	Methyl galactoside (1S,2S,3S,4R,5R)-	$6.37 \times 10^5 \pm 1.1 \times 10^6$	1/3	0	0/3	0	0/3	S1
PM292	Methyl xylopyranoside	1	Methyl xylopyranoside	$8.78 \times 10^4 \pm 1.52 \times 10^5$	1/3	0	0/3	0	0/3	S1
PM293	Methylsuccinic acid	1	Methylsuccinic acid	0	0/3	$2.3 \times 10^5 \pm 3.98 \times 10^5$	1/3	0	0/3	S2

Parent ID	Putative parent metabolite	Features (n)	Contributing retained feature form(s)	S1 mean ± SD (peak area)	S1 detected (n/3)	S2 mean ± SD (peak area)	S2 detected (n/3)	S3 mean ± SD (peak area)	S3 detected (n/3)	Dominant season
PM294	mono-Ethyl malonate	1	mono-Ethyl malonate	$1.86 \times 10^{7} \pm 7.93 \times 10^{6}$	3/3	$1.65 \times 10^{5} \pm 2.87 \times 10^{5}$	1/3	$7.71 \times 10^{6} \pm 2.57 \times 10^{6}$	3/3	S1
PM295	Monoamidomalonic acid	1	Monoamidomalonic acid	$1.21 \times 10^{6} \pm 2.1 \times 10^{6}$	1/3	$8.6 \times 10^{5} \pm 1.49 \times 10^{6}$	1/3	0	0/3	S1
PM296	Monoethyl methylmalonate	1	Monoethyl methylmalonate	0	0/3	0	0/3	$7.45 \times 10^{5} \pm 1.29 \times 10^{6}$	1/3	S3
PM297	Monoethyl succinate	1	Monoethyl succinate	$9.84 \times 10^{5} \pm 3.38 \times 10^{5}$	3/3	$1.52 \times 10^{7} \pm 4.56 \times 10^{6}$	3/3	$1.03 \times 10^{7} \pm 1.9 \times 10^{6}$	3/3	S2
PM298	Myo-Inositol	1	Myo-Inositol	$1.55 \times 10^{9} \pm 4.55 \times 10^{7}$	3/3	$1.02 \times 10^{9} \pm 3.79 \times 10^{7}$	3/3	$1.6 \times 10^{9} \pm 1.17 \times 10^{8}$	3/3	S3
PM299	Myo-Inositol, phosphate	1	Myo-Inositol, phosphate	$3.3 \times 10^{5} \pm 5.72 \times 10^{5}$	1/3	$3.12 \times 10^{6} \pm 5.41 \times 10^{6}$	1/3	0	0/3	S2
PM300	Myristic acid	1	Myristic acid	0	0/3	$1.79 \times 10^{8} \pm 1.66 \times 10^{7}$	3/3	$3.1 \times 10^{7} \pm 6.62 \times 10^{6}$	3/3	S2
PM301	N,N-Dimethyl-2-aminoethanol	1	N,N-Dimethyl-2-aminoethanol	0	0/3	$1.71 \times 10^{6} \pm 1.15 \times 10^{6}$	3/3	0	0/3	S2
PM302	N-Acetyl-D-glucosamine	1	N-Acetyl-D-glucosamine, methyloxime (syn)	0	0/3	$1.82 \times 10^{6} \pm 1.89 \times 10^{6}$	2/3	$4.62 \times 10^{5} \pm 8 \times 10^{5}$	1/3	S2
PM303	N-Formylglycine	1	N-Formylglycine	0	0/3	0	0/3	$3.14 \times 10^{5} \pm 5.43 \times 10^{5}$	1/3	S3
PM304	Neophytadiene	1	Neophytadiene	0	0/3	$1.74 \times 10^{7} \pm 4.57 \times 10^{5}$	3/3	$2.71 \times 10^{6} \pm 4.69 \times 10^{6}$	1/3	S2
PM305	Octacosane	1	Octacosane	0	0/3	$5.52 \times 10^{5} \pm 9.56 \times 10^{5}$	1/3	0	0/3	S2
PM306	Octacosanoate	1	Octacosanoate	0	0/3	0	0/3	$3.15 \times 10^{6} \pm 3.94 \times 10^{5}$	3/3	S3
PM307	Oxalic acid	1	Oxalic acid	$5.4 \times 10^{6} \pm 7.41 \times 10^{6}$	2/3	0	0/3	$7.92 \times 10^{6} \pm 9 \times 10^{6}$	2/3	S3
PM308	p-Coumaric acid	1	4-Coumaric acid	0	0/3	$6.17 \times 10^{5} \pm 1.07 \times 10^{6}$	1/3	$1.76 \times 10^{7} \pm 7.58 \times 10^{6}$	3/3	S3
PM309	Palatinose	1	Palatinose, methyloxime	$3.12 \times 10^{7} \pm 1.42 \times 10^{7}$	3/3	$1.25 \times 10^{6} \pm 1.12 \times 10^{6}$	2/3	$1.88 \times 10^{7} \pm 1.42 \times 10^{7}$	3/3	S1
PM310	Palmitic acid	1	Palmitic Acid	$3.38 \times 10^{8} \pm 1.15 \times 10^{7}$	3/3	$1.62 \times 10^{9} \pm 1.85 \times 10^{7}$	3/3	$5.5 \times 10^{8} \pm 5.44 \times 10^{7}$	3/3	S2
PM311	Pentadecanoic acid	1	Pentadecanoic acid	0	0/3	$3.42 \times 10^{6} \pm 6.48 \times 10^{5}$	3/3	0	0/3	S2
PM312	Pentanedioic acid	1	Pentanedioic acid	$6.11 \times 10^{6} \pm 6.62 \times 10^{6}$	3/3	$6.12 \times 10^{6} \pm 3.97 \times 10^{6}$	3/3	$4.42 \times 10^{6} \pm 6.95 \times 10^{6}$	2/3	S2
PM313	Pentenoic acid	1	Pentenoic acid	0	0/3	0	0/3	$4.72 \times 10^{7} \pm 8.17 \times 10^{7}$	1/3	S3

Parent ID	Putative parent metabolite	Features (n)	Contributing retained feature form(s)	S1 mean ± SD (peak area)	S1 detected (n/3)	S2 mean ± SD (peak area)	S2 detected (n/3)	S3 mean ± SD (peak area)	S3 detected (n/3)	Dominant season
PM314	Phenylalanine	1	Phenylalanine	0	0/3	0	0/3	$2.85 \times 10^6 \pm 1.86 \times 10^6$	3/3	S3
PM315	Phytol	1	Phytol	$2.11 \times 10^8 \pm 1.08 \times 10^7$	3/3	0	0/3	0	0/3	S1
PM316	Phytyl, 2-methylbutanoate	1	Phytyl, 2-methylbutanoate	0	0/3	0	0/3	$1.07 \times 10^5 \pm 1.85 \times 10^5$	1/3	S3
PM317	Pinacol	1	Pinacol	0	0/3	$2.02 \times 10^6 \pm 1.96 \times 10^6$	2/3	0	0/3	S2
PM318	Pinostrobin (5-hydroxy-7-methoxyflavanone)	1	5-hydroxy-7-methoxyflavanone	0	0/3	$2.05 \times 10^6 \pm 3.55 \times 10^6$	1/3	0	0/3	S2
PM319	Pregabalin	1	Pregabalin	$8.17 \times 10^5 \pm 1.41 \times 10^6$	1/3	0	0/3	0	0/3	S1
PM320	Propanedioic acid	1	Propanedioic acid	$5.26 \times 10^7 \pm 4.5 \times 10^6$	3/3	$7.29 \times 10^6 \pm 1.72 \times 10^6$	3/3	$7.13 \times 10^6 \pm 1.76 \times 10^6$	3/3	S1
PM321	Propanetriol, 2-methyl-	1	Propanetriol, 2-methyl-	$1.02 \times 10^8 \pm 8.87 \times 10^7$	2/3	$2.44 \times 10^7 \pm 2.12 \times 10^7$	2/3	$4.49 \times 10^7 \pm 1.26 \times 10^7$	3/3	S1
PM322	Propanoic acid, 2-(methoxyimino)-	1	Propanoic acid, 2-(methoxyimino)-	0	0/3	$2.45 \times 10^5 \pm 4.24 \times 10^5$	1/3	0	0/3	S2
PM323	Propanoic acid, 2-methyl-	1	Propanoic acid, 2-methyl-	$8.04 \times 10^4 \pm 1.39 \times 10^5$	1/3	$6.72 \times 10^6 \pm 1.31 \times 10^6$	3/3	$6.09 \times 10^6 \pm 1.22 \times 10^6$	3/3	S2
PM324	Propylene glycol	1	Propylene glycol	$1.55 \times 10^5 \pm 1.4 \times 10^5$	2/3	0	0/3	0	0/3	S1
PM325	Pyroglutamic acid	1	Pyroglutamic acid	$7.46 \times 10^5 \pm 1.29 \times 10^6$	1/3	0	0/3	0	0/3	S1
PM326	Pyruvic acid	1	Pyruvic acid	$4.96 \times 10^5 \pm 8.59 \times 10^5$	1/3	0	0/3	$9.23 \times 10^5 \pm 1.6 \times 10^6$	1/3	S3
PM327	Rhamnitol, 1-O-nonyl-	1	Rhamnitol, 1-O-nonyl-	$2.93 \times 10^5 \pm 5.08 \times 10^5$	1/3	0	0/3	0	0/3	S1
PM328	Ribitol	1	Ribitol	0	0/3	$1.03 \times 10^8 \pm 5.96 \times 10^6$	3/3	$5.66 \times 10^7 \pm 9.27 \times 10^6$	3/3	S2
PM329	Ribonic acid	1	Ribonic acid	$5 \times 10^7 \pm 7.55 \times 10^6$	3/3	$9.09 \times 10^6 \pm 3.81 \times 10^6$	3/3	$9.05 \times 10^6 \pm 1.56 \times 10^6$	3/3	S1
PM330	Scyllo-Inositol	1	Scyllo-Inositol	$1.22 \times 10^7 \pm 1.2 \times 10^6$	3/3	$2.97 \times 10^6 \pm 5.14 \times 10^6$	1/3	$1.14 \times 10^8 \pm 1.03 \times 10^7$	3/3	S3
PM331	Sebacic acid	1	Sebacic acid	0	0/3	0	0/3	$2.1 \times 10^6 \pm 2.36 \times 10^5$	3/3	S3
PM332	Serine	1	Serine	0	0/3	0	0/3	$4.47 \times 10^7 \pm 1.14 \times 10^7$	3/3	S3
PM333	Squalene	1	Squalene	$4.24 \times 10^7 \pm 3.44 \times 10^5$	3/3	0	0/3	$7.87 \times 10^7 \pm 9.59 \times 10^6$	3/3	S3

Parent ID	Putative parent metabolite	Features (n)	Contributing retained feature form(s)	S1 mean ± SD (peak area)	S1 detected (n/3)	S2 mean ± SD (peak area)	S2 detected (n/3)	S3 mean ± SD (peak area)	S3 detected (n/3)	Dominant season
PM334	Stearic acid	1	Stearic acid	$1.29 \times 10^8 \pm 1.06 \times 10^7$	3/3	$2.85 \times 10^8 \pm 1.65 \times 10^7$	3/3	$2.13 \times 10^8 \pm 2.66 \times 10^7$	3/3	S2
PM335	Stigmast-5-ene, 3.beta., (24S)-	1	Stigmast-5-ene, 3.beta., (24S)-	0	0/3	$2.36 \times 10^8 \pm 1.26 \times 10^7$	3/3	$5.79 \times 10^7 \pm 7.9 \times 10^6$	3/3	S2
PM336	Stigmastanol	1	Stigmastanol	0	0/3	$5.75 \times 10^6 \pm 6.02 \times 10^5$	3/3	0	0/3	S2
PM337	Stigmasterol	1	Stigmasterol	$5.33 \times 10^7 \pm 1.44 \times 10^6$	3/3	$2.67 \times 10^8 \pm 1.35 \times 10^7$	3/3	$6.5 \times 10^7 \pm 8.59 \times 10^6$	3/3	S2
PM338	Sucrose	1	Sucrose	$6.54 \times 10^8 \pm 4.67 \times 10^7$	3/3	$2.49 \times 10^8 \pm 4.27 \times 10^7$	3/3	$4.12 \times 10^8 \pm 7.22 \times 10^7$	3/3	S1
PM339	Tetraethylene glycol diethyl ether	1	Tetraethylene glycol diethyl ether	0	0/3	$8.82 \times 10^5 \pm 1.53 \times 10^6$	1/3	0	0/3	S2
PM340	Tetrapentacontane	1	Tetrapentacontane	$1.35 \times 10^6 \pm 2.33 \times 10^6$	1/3	0	0/3	0	0/3	S1
PM341	Thymol-beta-D-glucopyranoside	1	Thymol-.beta.-d-glucopyranoside	$1.84 \times 10^7 \pm 3.19 \times 10^7$	1/3	0	0/3	0	0/3	S1
PM342	Traumatic acid, (E)-	1	Traumatic acid, (E)-	0	0/3	0	0/3	$1.85 \times 10^7 \pm 2.66 \times 10^6$	3/3	S3
PM343	Tripropylene glycol monomethyl ether	1	Tripropylene glycol monomethyl ether	0	0/3	$6.04 \times 10^4 \pm 1.05 \times 10^5$	1/3	0	0/3	S2
PM344	Uracil	1	Uracil	0	0/3	$7.93 \times 10^6 \pm 7.82 \times 10^6$	2/3	$7.86 \times 10^6 \pm 2.08 \times 10^4$	3/3	S2
PM345	Uridine	1	Uridine	$9.07 \times 10^6 \pm 1.57 \times 10^7$	1/3	$1.61 \times 10^7 \pm 5.52 \times 10^5$	3/3	$1.89 \times 10^7 \pm 2.8 \times 10^6$	3/3	S3
PM346	Vanillylmandelic acid	1	Vanillylmandelic acid	$7.3 \times 10^6 \pm 1.27 \times 10^7$	1/3	$3.08 \times 10^6 \pm 5.34 \times 10^6$	1/3	0	0/3	S1
PM347	Venlafaxine	1	Venlafaxine	$4.27 \times 10^5 \pm 7.4 \times 10^5$	1/3	$8.49 \times 10^4 \pm 1.47 \times 10^5$	1/3	0	0/3	S1
PM348	Vinclozolin M2	1	Vinclozolin M2	$2.68 \times 10^6 \pm 4.64 \times 10^6$	1/3	0	0/3	0	0/3	S1
PM349	Xylitol	1	Xylitol	$8.7 \times 10^7 \pm 1.51 \times 10^8$	1/3	$6.12 \times 10^8 \pm 4.14 \times 10^7$	3/3	$1.7 \times 10^9 \pm 2.94 \times 10^9$	1/3	S3
PM350	Xylose	1	Xylose	$1.34 \times 10^7 \pm 4.14 \times 10^6$	3/3	0	0/3	0	0/3	S1

Dataset scope. The table lists all 350 consolidated parent metabolites used for seasonal comparison. All compound names are putative library-based annotations; none was confirmed using an authentic standard.

Seasonal groups. S1 = late summer (September 2024), S2 = autumn (November 2024), and S3 = winter (February 2025).

Peak-intensity reporting. Values are raw integrated GC–MS peak areas in arbitrary units and are presented as mean ± SD for three biological replicates. Zeros were retained and indicate that no peak area was recorded in the supplied processed matrix.

Parent-metabolite consolidation. When multiple retained analytical features mapped to the same parent metabolite, their replicate-level peak areas were summed before the seasonal mean, SD, and detection frequency were calculated.

Display precision. Peak areas are rounded to three significant digits in this Word table. Exact replicate-level values, retained-feature records, and derivatized-feature mappings are provided in Supplementary Data S1 (Excel).

Interpretation. "Dominant season" identifies the collection with the highest mean peak area and is descriptive; it does not indicate statistical significance. No retained entry in the supplied processed export was labelled as unidentified.

Supplementary Table S4. Season-specific two-way ANOVA of time (0 h vs 24 h), treatment, and their interaction on *E. coli* abundance in the phage–*Abutilon pannosum* synergy experiment.

Season	Effect	df	F	p-value	Partial η²
S1	Time	1, 32	171.30	<0.0001	0.843
	Treatment	7, 32	554.18	<0.0001	0.992
	Time × Treatment	7, 32	787.96	<0.0001	0.994
S2	Time	1, 32	1679.26	<0.0001	0.981
	Treatment	7, 32	194.35	<0.0001	0.977
	Time × Treatment	7, 32	221.59	<0.0001	0.980
S3	Time	1, 32	3026.13	<0.0001	0.990
	Treatment	7, 32	500.55	<0.0001	0.991
	Time × Treatment	7, 32	446.85	<0.0001	0.990

Supplementary Table S5. Priority antimicrobial-relevant metabolites identified from seasonal GC-MS profiling of Abutilon pannosum extracts and their descriptive alignment with antimicrobial screening and phage–extract activity. S1, late summer (September 2024); S2, autumn (November 2024); S3, winter (February 2025). Priority ranking is descriptive and intended for candidate prioritization rather than causal inference.

Metabolite	Enriched season	Evidence class	Reported antimicrobial targets	Phage-enhancement alignment	Integration interpretation	Priority
4-Hydroxybenzoic acid	S2	antibacterial	Gram-positive and selected Gram-negative bacteria	High	Most relevant to S. aureus and other Gram-positive screening endpoints.	High
5-Hydroxy-7-methoxyflavanone	S2	antibacterial	*Pseudomonas aeruginosa*	High	Indirect alignment; seasonality is informative but organism-level matching is less specific.	High
Citronellol	S2	antibacterial	*Escherichia coli*	High	Directly relevant to the E. coli screening/synergy endpoints.	High

Dodecanoic acid (lauric acid)	S2	antibacterial	*Staphylococcus aureus*	High	Most relevant to S. aureus and other Gram-positive screening endpoints.	High
Farnesol	S2	antibacterial + antifungal	*Staphylococcus aureus; Candida albicans*	High	Most relevant to S. aureus and other Gram-positive screening endpoints.	High
Ferulic acid	S2	antibacterial	Pathogenic bacteria including *Staphylococcus aureus*	High	Most relevant to S. aureus and other Gram-positive screening endpoints.	High
α-Linolenic acid	S2	antibacterial	*Bacillus cereus; Staphylococcus aureus*	High	Most relevant to S. aureus and other Gram-positive screening endpoints.	High
Phytol	S1	antibacterial + antifungal	*Pseudomonas aeruginosa; Candida spp.*	Moderate	Antifungal evidence; useful as a broader seasonal bioactivity marker.	Moderat e
Thymol-β-D-glucopyranoside	S1	antibacterial	*Campylobacter jejuni; Salmonella Typhimurium; Escherichia coli* K88	Moderate	Directly relevant to the E. coli screening/synergy endpoints.	Moderat e
Caffeic acid	S3	antibacterial	*Staphylococcus aureus* and related strains	High	Most relevant to S. aureus and other Gram-positive screening endpoints.	Moderat e
D-Limonene	S3	antibacterial	*Listeria monocytogenes*	High	Indirect alignment; seasonality is informative but organism-level matching is less specific.	Moderat e
p-Coumaric acid	S3	antibacterial	*Staphylococcus aureus* and other bacteria	High	Most relevant to S. aureus and other Gram-positive screening endpoints.	Moderat e
Stearic acid	S2	limited/condi tional	Selected bacteria or fungi in lipid-based assays	High	Indirect alignment; seasonality is informative but organism-level matching is less specific.	Low
Xylitol	S3	weak antifungal	*Candida albicans*	High	Antifungal evidence; useful as a broader seasonal bioactivity marker.	Low

Abbreviations: GC-MS, gas chromatography–mass spectrometry. Phage-enhancement alignment summarizes the descriptive correspondence between metabolite seasonal enrichment and the magnitude of phage-enhanced *E. coli* reduction; it does not indicate compound-level proof of synergy.

Supplementary Table S6. Mapping of retained derivatized GC–MS entries to parent metabolites used for biological interpretation. This table summarizes the retained derivatized GC–MS entries that were preserved in the analytical dataset but merged under parent metabolites for biological interpretation. Detection frequency is reported as the number of replicates (out of 3) in which each derivatized entry was observed for S1 (September 2024), S2 (November 2024), and S3 (February 2025).

Derivatized GC–MS entry	Parent metabolite used for interpretation	Derivatization / isomer note	Detection frequency across seasons (n/3 replicates)	Confidence / note
2-Ketoisocaproic acid oxime	2-Ketoisocaproic acid	oxime	S1 0/3; S2 0/3; S3 1/3	—
2-.alpha.-Mannobiose, methyloxime (isomer 2)	2-α-Mannobiose	methyloxime; isomer 2	S1 0/3; S2 2/3; S3 1/3	—
3-.alpha.-Mannobiose, methyloxime (isomer 1)	3-α-Mannobiose	methyloxime; isomer 1	S1 0/3; S2 0/3; S3 1/3	—
4-Ketoglucose, bis(O-methyloxime)	4-Ketoglucose	bis(O-methyloxime)	S1 1/3; S2 0/3; S3 3/3	—
D-(+)-Cellobiose, methyloxime (isomer 2)	D-(+)-Cellobiose	methyloxime; isomer 2	S1 3/3; S2 3/3; S3 3/3	—
D-(+)-Galactose oxime (isomer 1)	D-(+)-Galactose	oxime; isomer 1	S1 2/3; S2 0/3; S3 0/3	—
D-(+)-Galactose oxime (isomer 2)	D-(+)-Galactose	oxime; isomer 2	S1 3/3; S2 3/3; S3 3/3	—
D-(+)-Galactose, ethyloxime	D-(+)-Galactose	ethyloxime	S1 0/3; S2 1/3; S3 0/3	—
D-(+)-Glucose oxime	D-(+)-Glucose	oxime	S1 0/3; S2 3/3; S3 0/3	—
D-(+)-Glucuronic acid .gamma.-lactone, methyloxime (anti)	D-(+)-Glucuronic acid γ-lactone	methyloxime; anti	S1 1/3; S2 0/3; S3 0/3	—
D-(+)-Talose, methyloxime (syn)	D-(+)-Talose	methyloxime; syn	S1 0/3; S2 0/3; S3 1/3	—
D-(+)-Turanose, methyloxime	D-(+)-Turanose	methyloxime	S1 0/3; S2 2/3; S3 0/3	—
D-(+)-Xylose, methyloxime (anti)	D-(+)-Xylose	methyloxime; anti	S1 1/3; S2 0/3; S3 2/3	—
D-(-)-Erythrose, ethyloxime (isomer 1)	D-(-)-Erythrose	ethyloxime; isomer 1	S1 0/3; S2 0/3; S3 1/3	—
D-(-)-Rhamnose, methyloxime (anti)	D-(-)-Rhamnose	methyloxime; anti	S1 0/3; S2 0/3; S3 2/3	—
D-(-)-Rhamnose, methyloxime (syn)	D-(-)-Rhamnose	methyloxime; syn	S1 0/3; S2 0/3; S3 1/3	—
D-Allose, ethyloxime	D-Allose	ethyloxime	S1 0/3; S2 3/3; S3 3/3	—

Derivatized GC–MS entry	Parent metabolite used for interpretation	Derivatization / isomer note	Detection frequency across seasons (n/3 replicates)	Confidence / note
D-Allose, oxime	D-Allose	oxime	S1 3/3; S2 1/3; S3 3/3	—
D-Altrose, ethyloxime	D-Altrose	ethyloxime	S1 1/3; S2 0/3; S3 1/3	—
D-Altrose, pentafluorobenzyloxime	D-Altrose	pentafluorobenzyloxime	S1 0/3; S2 0/3; S3 1/3	—
D-Arabinose, ethyloxime	D-Arabinose	ethyloxime	S1 3/3; S2 3/3; S3 3/3	—
D-Arabinose, methyloxime (syn)	D-Arabinose	methyloxime; syn	S1 0/3; S2 0/3; S3 1/3	—
D-Fructose, O-methyloxime	D-Fructose	O-methyloxime	S1 0/3; S2 0/3; S3 1/3	—
D-Glucose, O-methyloxime	D-Glucose	O-methyloxime	S1 0/3; S2 3/3; S3 0/3	—
D-Glycero-D-gulo-Heptose, O-methyloxime	D-Glycero-D-gulo-Heptose	O-methyloxime	S1 3/3; S2 3/3; S3 3/3	—
D-Lactose, methyloxime	D-Lactose	methyloxime	S1 1/3; S2 1/3; S3 1/3	—
D-Psicose, methyloxime (syn)	D-Psicose	methyloxime; syn	S1 3/3; S2 0/3; S3 0/3	—
DL-Arabinose, methyloxime	DL-Arabinose	methyloxime	S1 2/3; S2 0/3; S3 0/3	—
L-(+)-Erythrulose, ethyloxime	L-(+)-Erythrulose	ethyloxime	S1 1/3; S2 0/3; S3 2/3	—
L-(+)-Threose, ethyloxime	L-(+)-Threose	ethyloxime	S1 0/3; S2 0/3; S3 1/3	—
Lactulose, methyloxime	Lactulose	methyloxime	S1 1/3; S2 2/3; S3 3/3	—
Maltose, methyloxime	Maltose	methyloxime	S1 3/3; S2 3/3; S3 3/3	—
N-Acetyl-D-glucosamine, methyloxime (syn)	N-Acetyl-D-glucosamine	methyloxime; syn	S1 0/3; S2 2/3; S3 1/3	—
Palatinose, methyloxime	Palatinose	methyloxime	S1 3/3; S2 2/3; S3 3/3	—

Derivatized GC–MS entry	Parent metabolite used for interpretation	Derivatization / isomer note	Detection frequency across seasons (n/3 replicates)	Confidence / note
Butanal, 2,3,4-tris[oxy]-3-[[oxy]methyl]-, O-methyloxime, (S)-	Polyhydroxy butanal derivative (tentative)	O-methyloxime; S	S1 3/3; S2 0/3; S3 0/3	Tentative parent assignment; interpreted cautiously.
.beta.-Gentiobiose, methyloxime	β-Gentiobiose	methyloxime	S1 3/3; S2 0/3; S3 0/3	—

Abbreviations: S1, September 2024; S2, November 2024; S3, February 2025.
Note: Unless otherwise indicated, all listed derivatized entries were merged under parent metabolites for downstream biological interpretation.

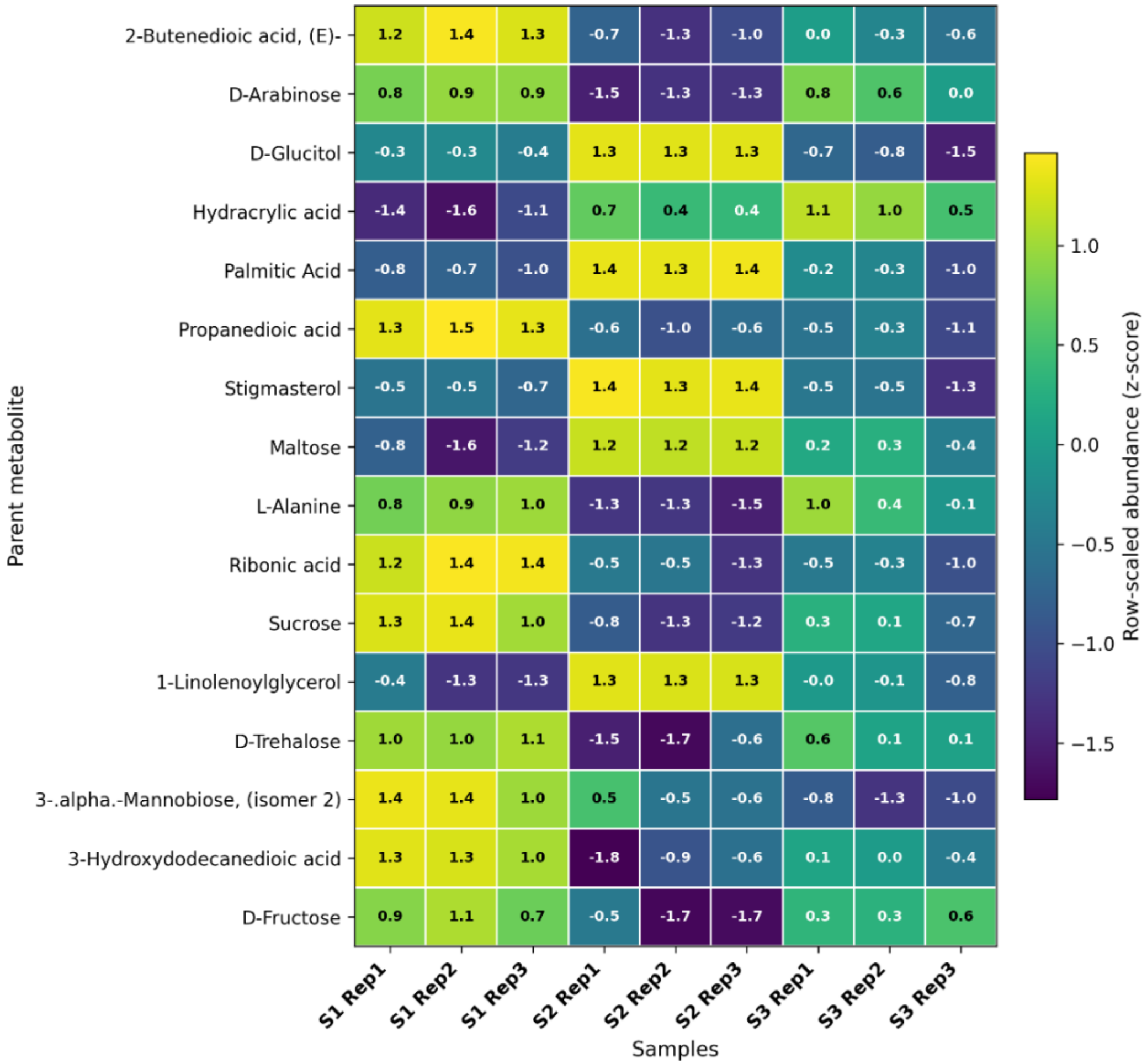

Supplementary Figure S1. Heatmap of the 16 significantly seasonal parent metabolites in *Abutilon pannosum* across individual samples from three seasonal collections (S1–S3). Values inside cells represent row-scaled abundance (z-score). S1, S2, and S3 denote the late-summer (September 2024), autumn (November 2024), and winter (February 2025) collections, respectively.

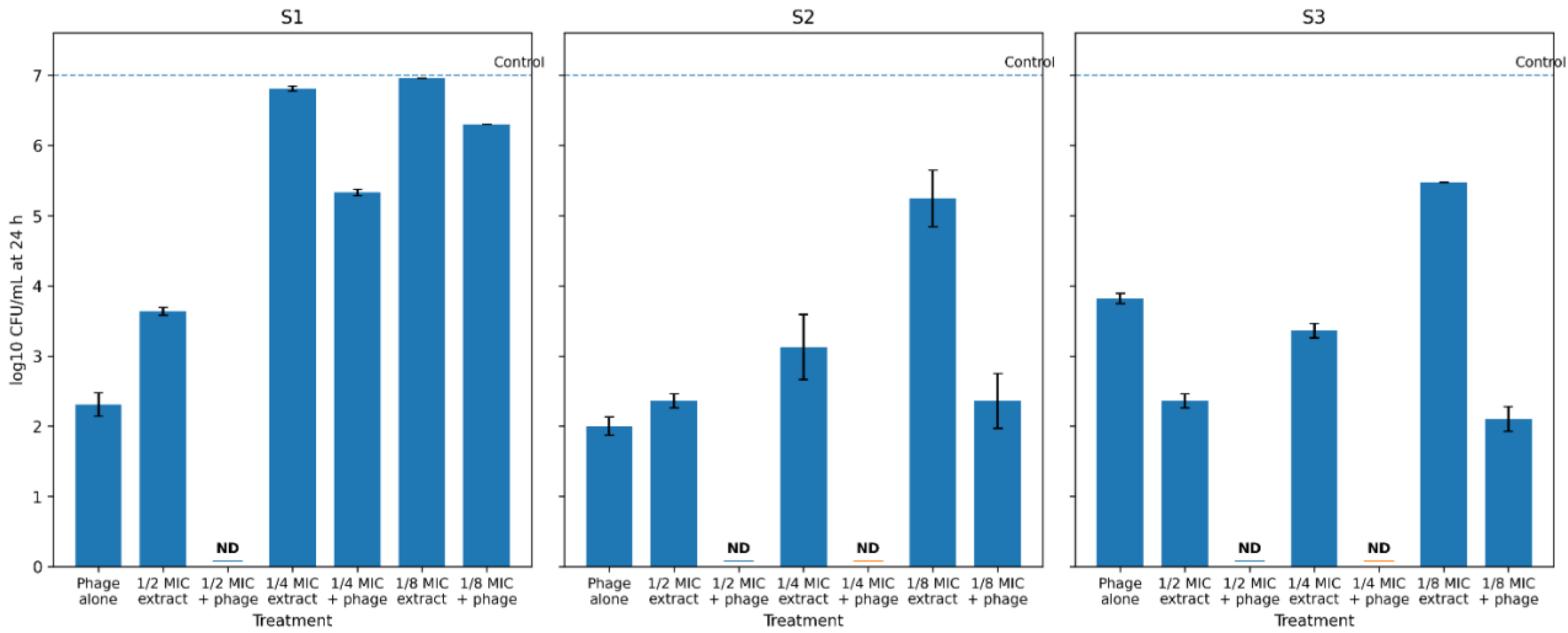

Supplementary Figure S2. Effect of phage and *Abutilon pannosum* extracts, alone and in combination, on *Escherichia coli* O157:H7 across three seasonal collections (S1–S3). Bars show mean log10 CFU/mL at 24 h ± SD (n = 3) for phage alone, sub-MIC extract treatments, and the corresponding extract–phage combinations. S1, S2, and S3 represent the late-summer, autumn, and winter collections, respectively.

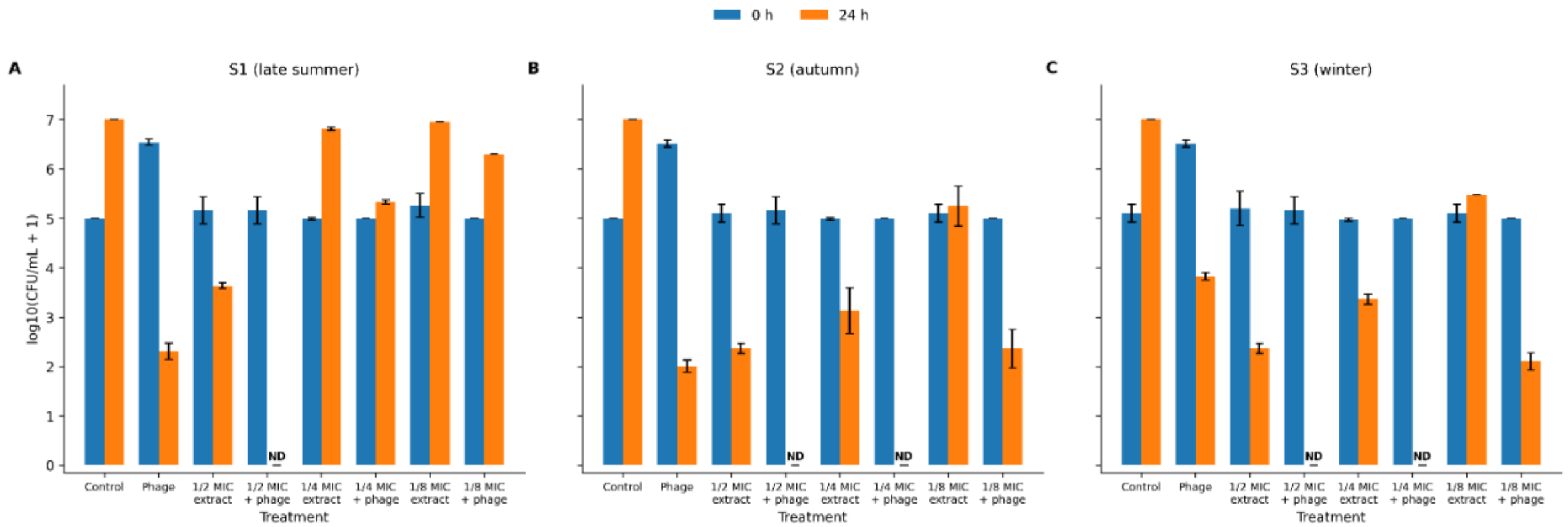

Supplementary Figure S3. Changes in *E. coli* abundance from 0 h to 24 h under phage and seasonal *Abutilon pannosum* extract treatments. Bars represent mean log10(CFU/mL + 1) ± SD for the control, phage alone, extract alone, and extract–phage combination treatments across three seasonal collections: S1, late summer (September 2024); S2, autumn (November 2024); and S3, winter (February 2025). ND indicates no detectable colonies in the corresponding 24 h treatment group.

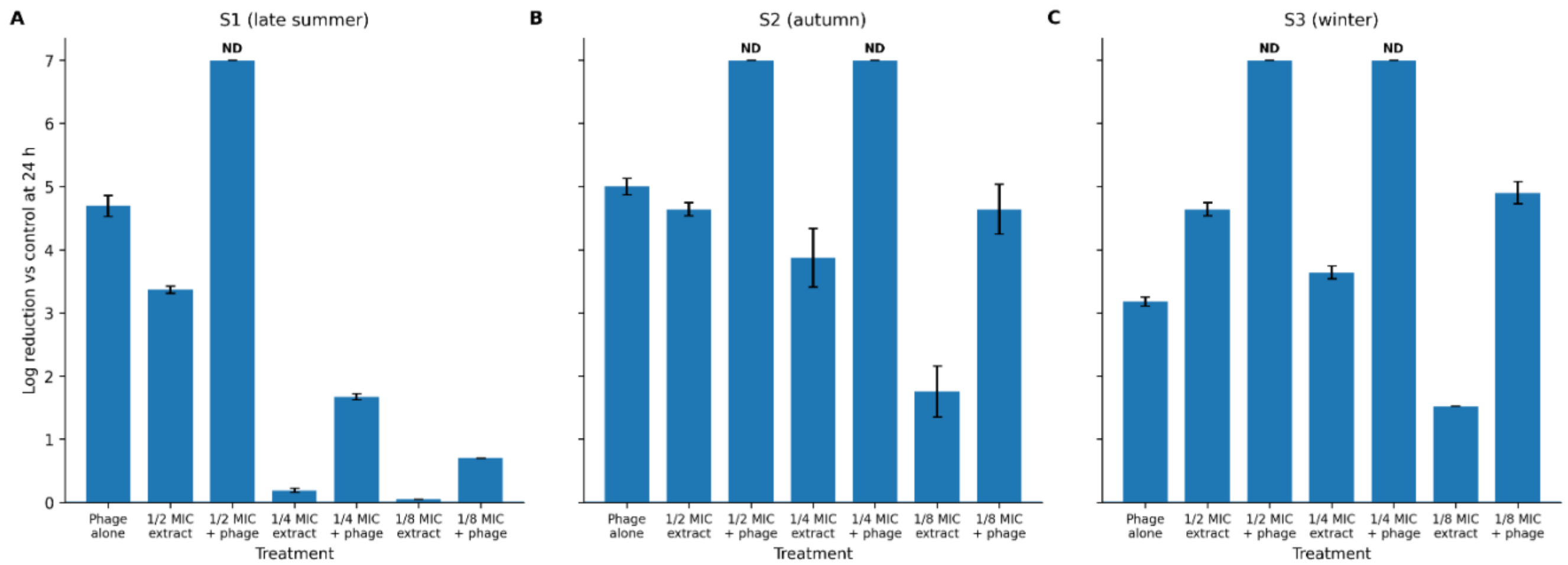

Supplementary Figure S4. Log reduction in *E. coli* relative to the untreated control after 24 h under phage and seasonal *Abutilon pannosum* extract treatments. Bars represent mean log reduction ± SD across three seasonal collections: S1, late summer (September 2024); S2, autumn (November 2024); and S3, winter (February 2025). ND indicates no detectable colonies in the corresponding treatment group.

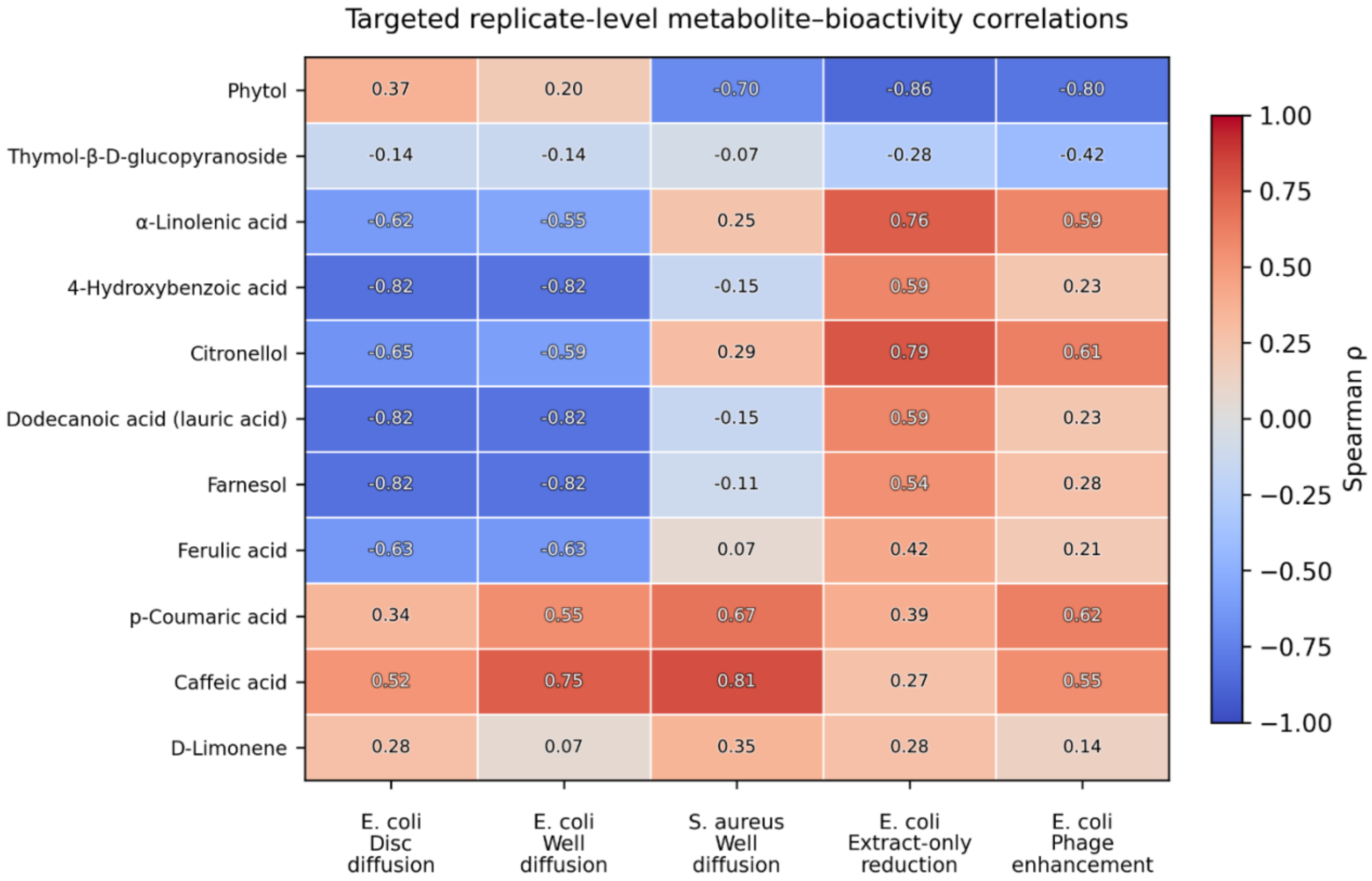

Supplementary Figure S5. Targeted replicate-level correlations between selected antimicrobial-relevant metabolites and bioactivity endpoints of seasonal *Abutilon pannosum* extracts. Heatmap values represent Spearman's ρ based on matched extract replicates across S1–S3. Positive values indicate higher metabolite abundance associated with stronger activity, whereas negative values indicate the oppo
